# Circulating tumour DNA in patients with stage III colon cancer: multicentre prospective PROVENC3 study

**DOI:** 10.1093/bjs/znaf281

**Published:** 2026-01-09

**Authors:** Carmen Rubio-Alarcón, Andrew Georgiadis, Ingrid A Franken, Haoyue Wang, Sietske C M W van Nassau, Suzanna J Schraa, Dave E W van der Kruijssen, Karlijn van Rooijen, Theodora C Linders, Pien Delis-van Diemen, Maartje Alkemade, Anne Bolijn, Marianne Tijssen, Margriet Lemmens, Lana Meiqari, Steven L C Ketelaars, Adria Closa-Mosquera, Miranda M W van Dongen, Mirthe Lanfermeijer, Birgit I Lissenberg-Witte, Linda J W Bosch, Teunise Bisschop-Snetselaar, Bregje C Adriaans, Amy Greer, David Riley, James R White, Christopher Greco, Liam Cox, Jesse Fox, Kaitlin Victor, Catherine Leech, Samuel V Angiuoli, Niels F M Kok, Cornelis J A Punt, Daan van den Broek, Miriam Koopman, Gerrit A Meijer, Victor E Velculescu, Jeanine M L Roodhart, Veerle M H Coupé, Mark Sausen, Geraldine R Vink, Remond J A Fijneman, Other Members of the PLCRC-MEDOCC Group, M Los, J M van Dodewaard, M L R Tjin-A-Ton, A H W Schiphorst, M Koopman, P J Tanis, K Talsma, H B A C Stockmann, D D E Zimmerman, W J Vles, L B J Valkenburg, R C Rietbroek, E J Th Belt, J W T Dekker, I H J T de Hingh, R W M Schrauwen, F J F Jeurissen, R Hoekstra, M P Hendriks, J Jansen, J Konsten, P Nieboer, N A J B Peters, J F M Pruijt, L Simkens, A I de Vos, A Schouten van der Velden, A W Haringhuizen, M A Davidis, J Janssen

**Affiliations:** Department of Pathology, The Netherlands Cancer Institute, Amsterdam, The Netherlands; Labcorp, Baltimore, Maryland, USA; Department of Medical Oncology, University Medical Centre Utrecht, Utrecht University, Utrecht, The Netherlands; Department of Epidemiology and Data Science, Amsterdam University Medical Centres, Location VU Medical Centre, Amsterdam, The Netherlands; Department of Medical Oncology, University Medical Centre Utrecht, Utrecht University, Utrecht, The Netherlands; Department of Medical Oncology, University Medical Centre Utrecht, Utrecht University, Utrecht, The Netherlands; Department of Medical Oncology, University Medical Centre Utrecht, Utrecht University, Utrecht, The Netherlands; Department of Medical Oncology, University Medical Centre Utrecht, Utrecht University, Utrecht, The Netherlands; Department of Laboratory Medicine, The Netherlands Cancer Institute, Amsterdam, The Netherlands; Department of Pathology, The Netherlands Cancer Institute, Amsterdam, The Netherlands; Core Facility Molecular Pathology and Biobanking (CFMPB), The Netherlands Cancer Institute, Amsterdam, The Netherlands; Department of Pathology, The Netherlands Cancer Institute, Amsterdam, The Netherlands; Department of Pathology, The Netherlands Cancer Institute, Amsterdam, The Netherlands; Department of Pathology, The Netherlands Cancer Institute, Amsterdam, The Netherlands; Department of Pathology, The Netherlands Cancer Institute, Amsterdam, The Netherlands; Department of Pathology, The Netherlands Cancer Institute, Amsterdam, The Netherlands; Department of Pathology, The Netherlands Cancer Institute, Amsterdam, The Netherlands; Department of Pathology, The Netherlands Cancer Institute, Amsterdam, The Netherlands; Department of Laboratory Medicine, The Netherlands Cancer Institute, Amsterdam, The Netherlands; Department of Epidemiology and Data Science, Amsterdam University Medical Centres, Location VU Medical Centre, Amsterdam, The Netherlands; Department of Pathology, The Netherlands Cancer Institute, Amsterdam, The Netherlands; Department of Medical Oncology, University Medical Centre Utrecht, Utrecht University, Utrecht, The Netherlands; Department of Medical Oncology, University Medical Centre Utrecht, Utrecht University, Utrecht, The Netherlands; Labcorp, Baltimore, Maryland, USA; Labcorp, Baltimore, Maryland, USA; Labcorp, Baltimore, Maryland, USA; Labcorp, Baltimore, Maryland, USA; Labcorp, Baltimore, Maryland, USA; Labcorp, Baltimore, Maryland, USA; Labcorp, Baltimore, Maryland, USA; Labcorp, Baltimore, Maryland, USA; Labcorp, Baltimore, Maryland, USA; Department of Surgical Oncology, The Netherlands Cancer Institute, Amsterdam, The Netherlands; Department of Epidemiology, Julius Centre, University Medical Centre Utrecht, Utrecht University, Utrecht, The Netherlands; Department of Laboratory Medicine, The Netherlands Cancer Institute, Amsterdam, The Netherlands; Department of Medical Oncology, University Medical Centre Utrecht, Utrecht University, Utrecht, The Netherlands; Department of Pathology, The Netherlands Cancer Institute, Amsterdam, The Netherlands; The Sidney Kimmel Comprehensive Cancer Center, Johns Hopkins University School of Medicine, Baltimore, Maryland, USA; Department of Medical Oncology, University Medical Centre Utrecht, Utrecht University, Utrecht, The Netherlands; Department of Epidemiology and Data Science, Amsterdam University Medical Centres, Location VU Medical Centre, Amsterdam, The Netherlands; Labcorp, Baltimore, Maryland, USA; Department of Medical Oncology, University Medical Centre Utrecht, Utrecht University, Utrecht, The Netherlands; Department of Research and Development, Netherlands Comprehensive Cancer Organization, Utrecht, The Netherlands; Department of Pathology, The Netherlands Cancer Institute, Amsterdam, The Netherlands

## Abstract

**Background:**

Circulating tumour DNA (ctDNA) is a promising biomarker to guide clinical decision-making. The aim of this study was to investigate the prognostic value of postoperative ctDNA in patients with stage III colon cancer who received adjuvant chemotherapy (ACT).

**Methods:**

PROVENC3 was a multicentre prospective study of patients who underwent resection of pathological stage III colon cancer. Blood samples were collected at a median of 13 (interquartile range 4–20) days after resection. The presence of minimal residual disease was determined using Labcorp^®^ Plasma Detect™, a novel tumour-informed whole genome sequencing (WGS) ctDNA test. The primary endpoint was 3-year time to recurrence (TTR). ctDNA status was further combined with pathological risk status to investigate the combined prognostic value.

**Results:**

The median follow-up of the 209 patients who were included was 40 months. In total, 28 patients (13%) had detectable ctDNA after surgery. Postoperative ctDNA-positive patients had a worse TTR compared with ctDNA-negative patients (HR 6.2 (95% c.i. 3.4 to 11.2); *P* < 0.001). Of all ctDNA-positive patients, 36% did not develop recurrences during 3-year follow-up. Detectable ctDNA after ACT was associated with worse TTR (HR 7.9 (95% c.i. 3.9 to 15.9); *P* < 0.001). ctDNA status combined with pathological risk classification resulted in a 3-year recurrence risk that varied from 82% for pathological high-risk (pT4/N2) ctDNA-positive patients to 7% for pathological low-risk (pT1–3 N1) ctDNA-negative patients (HR 28.5 (95% c.i. 10.5 to 77.2); *P* < 0.001).

**Conclusion:**

Postoperative ctDNA detection using a tumour-informed WGS test improves prognosis stratification in stage III colon cancer and may help to personalize adjuvant treatment.

## Teaser

The prognostic value of circulating tumour DNA (ctDNA) was investigated in a prospective multicentre cohort study of patients with stage III colon cancer treated with adjuvant chemotherapy, using a novel biomarker assay for detecting minimal residual disease. Post-surgery ctDNA detection combined with pathological low-risk (pT1–3 N1) and high-risk (pT4/N2) classification improved current risk stratification, where the recurrence risk at 3 years ranged from 7% for low-risk ctDNA-negative patients to 82% for high-risk ctDNA-positive patients. These findings will lead to ctDNA-guided interventional (de-)escalation trials that aim to improve personalized disease management of patients with stage III colon cancer.

## Introduction

Surgery followed by adjuvant chemotherapy (ACT) is the standard of care for non-metastatic patients with node-positive (stage III) colon cancer^1^. Approximately 55% of patients are, however, cured by surgery alone and 30% experience a recurrence despite ACT^2,3^. As a consequence, only 15–20% of patients benefit from ACT, whilst all patients are exposed to the risks of treatment. Therefore, there is an urgent unmet need to identify those patients who are truly at risk of recurrence after surgery and could benefit from ACT and those who are at a low risk of developing recurrence and could avoid ACT.

Detection of circulating tumour DNA (ctDNA) in minimally invasive blood liquid biopsies is a clinically applicable technology to retrieve information about solid tumours, even when tissue biopsies cannot be obtained. One putative clinical application is the post-surgery detection of ctDNA in cell-free plasma as a biomarker for minimal residual disease (MRD)^4^. Recent studies have demonstrated that post-surgery ctDNA positivity is a strong prognostic biomarker for disease recurrence in stage II and III colon cancer. This could enable identification of patients at high risk of developing recurrence and guide clinical decisions^5–9^ . However, MRD detection after resection of the primary tumour is technically demanding due to extremely low levels of ctDNA^10^. These challenges have been primarily mitigated through tumour-informed, personalized ctDNA approaches to maximize sensitivity and specificity, which often require patient-specific bespoke panels to be developed. This introduces several operational and technical complexities into the assay workflow. This prolongs turnaround times for the post-surgery landmark test result and challenges incorporation of ctDNA-based MRD testing into specific clinical settings. Recent developments in the field have shown that tumour-informed whole genome sequencing (WGS) approaches hold promise for MRD testing, given the ability to track thousands of tumour-specific mutations without the need for personalized assay design, manufacture, and quality-control testing for each patient. These can result in landmark testing turnaround times of <14 days *versus* 4–6 weeks for bespoke, panel-based approaches based on a more limited number of alterations^11–14^. The first study evaluating WGS-based ctDNA detection was published recently^15^, yet the clinical performance remains to be fully established in independent, prospective clinical cohorts.

The ‘Prospective Dutch ColoRectal Cancer’ (PLCRC) cohort is a real-world, nationwide cohort study of patients with colorectal cancer (CRC) in the Netherlands. PROVENC3 was a prospective observational substudy of PLCRC, consisting of a large unselected group of patients treated according to the standard of care^16^. The aim of this study was to determine the prognostic value of post-surgery ctDNA status in patients with stage III colon cancer treated with ACT.

## Methods

### Patients

This was a multicentre prospective study of patients with stage III colon cancer who underwent ACT. Patients who were diagnosed with CRC, 18 years or older, and mentally competent were prospectively recruited in the Netherlands for participation in the ongoing PLCRC study (NCT02070146). Informed consent for the collection of long-term clinical and survival data was mandatory for participation in PLCRC; subsequently, patients were given the option to consent to: filling out questionnaires on health-related quality of life, functional outcomes, and workability; biobanking of tumour and normal tissue; collection of blood samples; and be included in studies conducted within the infrastructure of the cohort. Clinical data were collected by the Netherlands Comprehensive Cancer Organization (IKNL) in the Netherlands Cancer Registry (NKR). Treatment-naïve non-metastatic CRC patients who gave informed consent for PLCRC and for additional blood sampling were included in the observational PLCRC substudy MEDOCC (‘Molecular Early Detection Of Colon Cancer’). Patients with pathological stage III colon cancer who started ACT after surgery and for whom post-surgery blood was available were included in the PLCRC-MEDOCC substudy PROVENC3 (listed under PLCRC, NCT02070146) in 26 hospitals from 2016 to 2021. As a substudy within a real-world cohort, post-surgery blood collection was performed as part of routinely scheduled blood withdrawals during standard-of-care visits. Patients with blood collected less than 3 days post-surgery were excluded from the study due to a potential increased risk of primarily false-negative results due to increased cell-free DNA levels due to surgically induced trauma. After curation of clinical data, two patients were included whose blood had been collected 2 days after resection of the primary tumour. Clinical data were collected via the NKR and through site visits by the PLCRC study team.

The PLCRC study was performed in accordance with the Declaration of Helsinki and approved by the Medical Ethical Committee Utrecht (NL47888.041.14). All patients signed written informed consent for study participation and collection of blood and tissue samples for translational research. The PLCRC substudy PROVENC3 was approved by the Institutional Review Board (IRB) of the Netherlands Cancer Institute, Amsterdam, The Netherlands (protocol CFMPB472).

### Sample collection and processing

Formalin-fixed paraffin-embedded (FFPE) tumour blocks were requested through ‘PALGA: Dutch Nationwide Pathology Databank’^17^, the network and registry of histopathology and cytopathology in the Netherlands. Haematoxylin and eosin (H&E)-stained slides were evaluated by a study-specific pathologist, the ‘tumour area’ was outlined on the slide for macrodissection, and DNA was isolated from that region (see the *supplementary methods*).

Two blood tubes (10 ml each) were collected pre-surgery (single time point), post-surgery (single time point before the start of ACT), after completion of ACT, and at 12, 18, 24, and 36 months. Blood was collected using a cell-stabilizing BCT tube (Streck, La Vista, NE, USA) in the participating hospitals and shipped to the Netherlands Cancer Institute. DNA was isolated from white blood cells (WBCs) and from cell-free plasma (see the *supplementary methods*). Samples were deidentified and blinded, then shipped to Personal Genome Diagnostics (Labcorp, Baltimore, MD, USA) for sample testing and analysis. Post-surgery ctDNA was evaluated for all patients in the cohort.

### WGS-based tumour-informed plasma ctDNA detection

Labcorp^®^ Plasma Detect™ is a novel tumour-informed WGS-based plasma ctDNA test for detection of MRD after a curative-intent intervention, including surgery or ACT. In brief, WGS data of tumour DNA (targeting 80× coverage) were compared with WGS data of WBC-derived germline DNA (targeting 40× coverage) to detect thousands of tumour-specific mutations that were unique to each patient. Next, WGS data obtained from 10 ng plasma-derived cell-free DNA (targeting 30× coverage) were used to search for the presence of tumour-specific mutations in blood draws, using a proprietary and independently validated bioinformatics pipeline. Labcorp^®^ Plasma Detect™ then provided a result for ctDNA status together with an estimated tumour fraction for the samples considered ctDNA positive, referred to as the ‘aggregate ctDNA variant allele fraction (VAF)’. A detailed description of the Labcorp^®^ Plasma Detect™ ctDNA test and its analytical test performance is provided in the *Supplementary material*.

### Sample size calculation

The aims of this prospective observational study were: to evaluate the percentage of post-surgery ctDNA-positive patients using a novel tumour-informed WGS plasma ctDNA test in a real-world cohort; and to evaluate the proportion of post-surgery ctDNA-positive patients who experienced recurrence at 3 years despite treatment with ACT in comparison with the proportion of post-surgery ctDNA-negative patients who experienced recurrence at 3 years despite treatment with ACT. Because the latter aim required a larger number of patients, the rationale for the sample size calculation was based on this aim. ctDNA positivity of 15% and an overall recurrence rate at 3 years of 32% were assumed. To detect a difference in the 3-year recurrence rate of at least 25% (28% in ctDNA-negative patients and 53% in ctDNA-positive patients), 189 patients were required using a two-sided log rank test with a significance level of 5% and a power of 80%. To account for 10% dropout, 210 patients were required.

### Statistical analysis

The prognostic value of ctDNA status was evaluated using time to recurrence (TTR) as the outcome measure. For TTR analyses, only recurrences were considered as events and patients without a recurrence were censored at the last visit to the treating physician, regardless of survival status^18^. Follow-up was censored at year 3 if follow-up was longer. For univariable TTR analyses, the Kaplan–Meier estimator and fitted Cox regression models were used. The pathological variables evaluated in the univariable models were selected based on clinical relevance: pathological risk status (low risk = T1–3 N1, high risk = T4 and/or N2), T status determined by the pathology report (T1–3, T4), N status determined by the pathology report (N1, N2), and microsatellite instability (MSI) status determined by next-generation sequencing of the primary tumour (stable, instable). The change in the HRs was also evaluated in univariable models after adding ctDNA status to each of the pathological covariates (pathological risk + ctDNA status, T status + ctDNA status, N status + ctDNA status, MSI status + ctDNA status). Furthermore, to evaluate whether post-surgery ctDNA status had added and independent predictive value for recurrence in addition to multiple pathological variables, several (multivariable) Cox regression models were fitted. First, the added value of ctDNA status was determined by fitting multivariable models combining the pathological risk factors and ctDNA status and performing likelihood ratio tests (LRTs) (LRT1 = pathological risk *versus* pathological risk + ctDNA status, LRT2 = pathological risk + MSI status *versus* pathological risk + MSI status + ctDNA status, LRT3 = T status + N status *versus* T status + N status + ctDNA status, LRT4 = T status + N status + MSI status *versus* T status + N status + MSI status + ctDNA status). Second, the independent predictive value of each variable in the model was evaluated by calculating the HRs of each variable independently in the two best models resulting from the LRTs (model 1 = pathological risk + MSI status + ctDNA status, model 2 = T status + N status +MSI status + ctDNA status). All statistical and survival analyses were performed using the R package ‘survival’ (https://cran.r-project.org/web/packages/survival/index.html; R version 4.4.1). Differences in baseline characteristics for the post-surgery ctDNA-positive group *versus* the post-surgery ctDNA-negative group were analysed using Fisher’s exact test for categorical variables and the Mann–Whitney test for continuous variables.

## Results

### Study population

In total, 209 patients with stage III colon cancer treated with ACT were included in the PROVENC3 study (*Table S1*). Of these, 188 patients (90.0%) received 3 months of capecitabine + oxaliplatin (CAPOX) as adjuvant treatment. The other patients were treated with 6 months of CAPOX or 5-fluorouracil + leucovorin + oxaliplatin (FOLFOX) (4.8%) or 6 months of capecitabine monotherapy (5.2%). Post-surgery blood samples were obtained and analysed for all 209 patients. In addition, pre-surgery and post-ACT blood samples were analysed for 148 and 171 patients respectively (*Fig. S1*). The median follow-up was 40 (interquartile range (i.q.r.) 21–57) months and 47 patients (22.5%) developed a recurrence within 3 years post-surgery. The median follow-up for patients not developing a recurrence was 45 (range 38–74) months.

### Analytical performance of the ctDNA detection test

Post-surgery detection of ctDNA indicates the presence of MRD; however, application of a highly sensitive tumour-informed plasma ctDNA test is essential to enable detection of the extremely low levels of ctDNA in this clinical setting. The novel Labcorp^®^ Plasma Detect™ ctDNA test was demonstrated to have a high analytical sensitivity, with a 95% limit of detection of 0.005% tumour content and a 50% limit of detection of 0.001% tumour content through integrated WGS analyses of patient-matched FFPE tumour-tissue DNA, WBC-derived normal germline DNA, and plasma cell-free DNA (*supplementary results*, *Fig. S2*, and *Tables S2–S4*). Moreover, among 148 patients for whom pre-surgery blood samples were available, ctDNA could be detected in 135 patients (91.2%), underscoring the high clinical sensitivity of the ctDNA test.

### Post-surgery detection of ctDNA

Post-surgery blood was available for 241 patients; however, 32 samples could not be analysed due to insufficient tumour tissue (3 samples), insufficient tissue DNA (5 samples), insufficient plasma DNA (5 samples), or quality-control failure (19 samples) (*Fig. S1b*). Post-surgery ctDNA status was determined in 209 patients at a landmark time point with a single blood draw at a median of 13 (i.q.r. 4–20) days after surgery (*Table S5*). Of 209 patients, 28 patients (13.4%) were ctDNA positive and 181 patients (86.6%) were ctDNA negative post-surgery. The post-surgery median aggregate ctDNA VAF was 0.035% (range 0.01–3.13%) (*Table S5*). From the baseline pathological features evaluated, only irradical resection was associated with post-surgery ctDNA positivity (*Table S1*). Pre-surgery ctDNA status was not related to post-surgery ctDNA status (Fisher's exact test *P* = 0.741).

Surgical trauma leads to increased levels of cell-free DNA in blood during the first week post-surgery, which may obscure and confound ctDNA detection and therefore lead to false-negative results^19^. Relative to the day of surgery, 30.6% of the post-surgery blood samples were collected before day 7, 21.0% between days 7 and 13, 26.8% between days 14 and 20, and 21.5% from day 21 onward after surgery. In the present study, using the Labcorp^®^ Plasma Detect™ ctDNA test, there was no difference in post-surgery ctDNA detection when blood was collected within 1 week post-surgery or at later time points (*Fig. S3*).

### Prognostic value of post-surgery ctDNA status

**Fig. 1:**
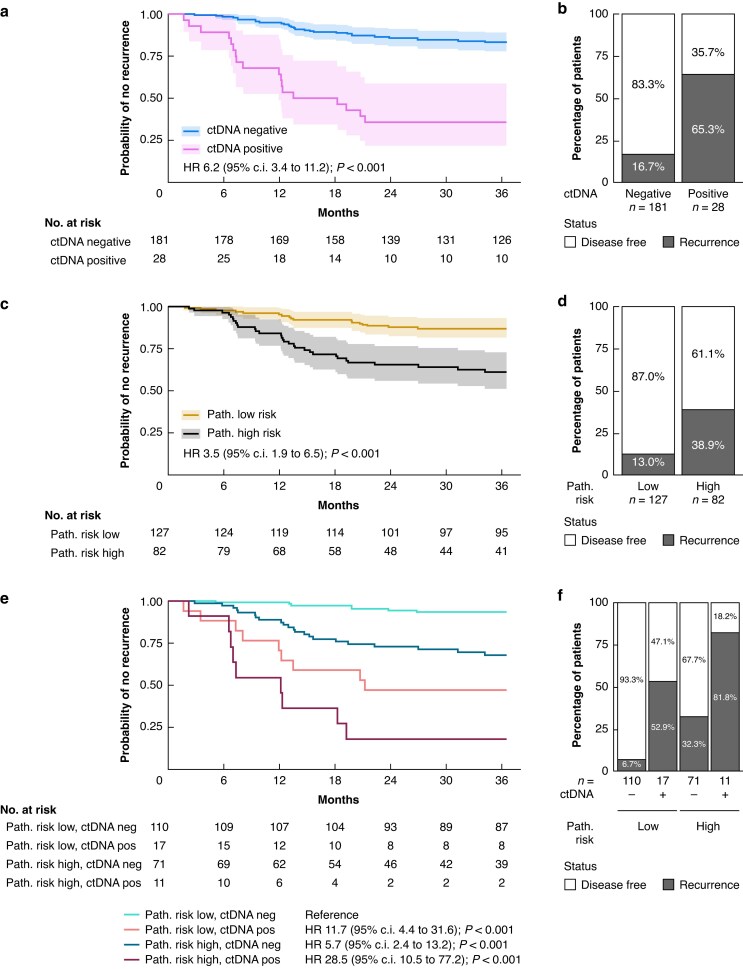
Detection of ctDNA post-surgery is independently associated with recurrence at 3 years in ACT-treated stage III colon cancer **a** Kaplan–Meier estimate for TTR stratified by post-surgery ctDNA status; censored patients are indicated with a vertical line. **b** Proportion of patients at risk of recurrence after 3 years. **c** Kaplan–Meier estimate for TTR stratified by pathological risk; censored patients are indicated with a vertical line. **d** Proportion of patients at risk of recurrence after 3 years. **e** Kaplan–Meier estimate for TTR stratified by pathological risk and ctDNA status; censored patients are indicated with a vertical line. **f** Proportion of patients at risk of recurrence after 3 years. ctDNA, circulating tumour DNA; ACT, adjuvant chemotherapy; TTR, time to recurrence; Path., pathological; ctDNA pos, ctDNA positive; ctDNA neg, ctDNA negative.

Post-surgery ctDNA-positive patients had a worse TTR compared with ctDNA-negative patients (HR 6.2 (95% c.i. 3.4 to 11.2); *P* < 0.001) (*Fig. 1a*,*b*). Among the 28 post-surgery ctDNA-positive patients, 10 patients (35.7%) remained disease free during 36-month follow-up, suggesting they may have benefited from treatment with ACT (*Fig. 1a*).

When assessing pathological risk, 127 patients (60.8%) had low-risk tumours (pT1–3 N1) and 82 patients (39.2%) had high-risk tumours (pT4 and/or pN2). In univariable analysis, pathological high-risk status was associated with recurrence (HR 3.5 (95% c.i. 1.9 to 6.5); *P* < 0.001) (*Fig. 1c*,*d* and *Table S6*). The prognostic value of the combination of pathological risk and post-surgery ctDNA status was evaluated in multivariable analysis. The recurrence risk of pathological high-risk patients was further increased when patients were ctDNA positive, while the recurrence risk of pathological low-risk patients was further decreased when patients were ctDNA negative. Consequently, there is a profound TTR difference between pathological high-risk ctDNA-positive patients (11 patients) and pathological low-risk ctDNA-negative patients (110 patients) (3-year risk of recurrence of 81.8% *versus* 6.7% respectively; HR 28.5 (95% c.i. 10.5 to 77.2); *P* < 0.001) (*Fig. 1e*,*f*, *Table S7*, and *Fig. S4*). ctDNA status improved the multivariable Cox regression model including pathological risk and MSI status (LRT, *P* < 0.001) (*Table S8*; LRT1). ctDNA status was the strongest independent predictor of recurrence (HR 6.8) in a model that also included pathological risk (HR 4.0) and MSI status (HR 0.7, not significant) (*Table S9*). In addition, post-surgery ctDNA status remained the strongest predictor of recurrence in multivariable models that included the effects of tumour (T), node (N), and MSI status as independent risk factors (*Tables S6–S9*).

Among the 47 of 209 patients (22.5%) who experienced a recurrence, post-surgery ctDNA-positive patients had a shorter TTR compared with ctDNA-negative patients (Mann–Whitney test, *P* = 0.030) (*Fig. S5a*). Because ctDNA shedding can be affected by the metastatic site, the association between recurrence location and post-surgery ctDNA status was examined. Eight of 11 patients (72.7%) with liver-limited metastases were ctDNA positive post-surgery, while 0 of 5 patients (0%) with peritoneal metastases were ctDNA positive post-surgery (*Fig. S5b*).

### Prognostic value of post-ACT ctDNA status

**Fig. 2:**
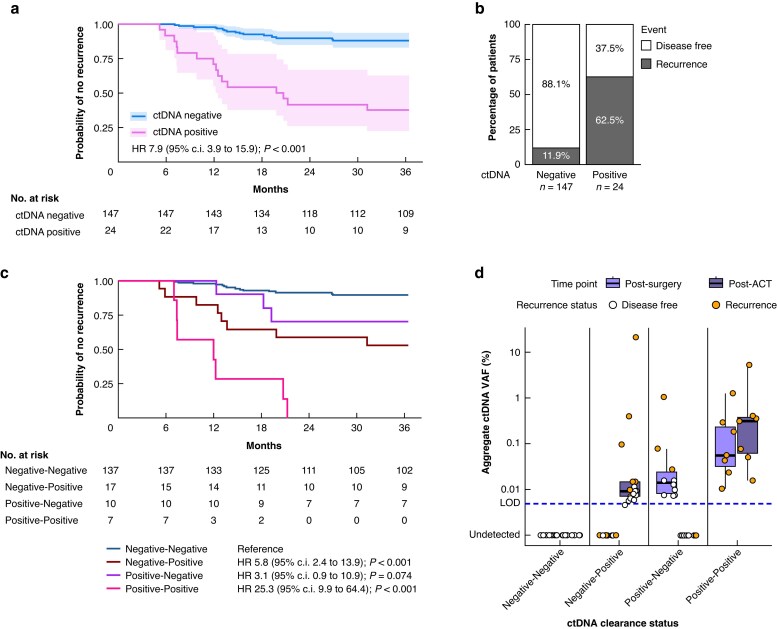
Evaluation of prognostic value of post-ACT ctDNA detection **a** Kaplan–Meier estimate for TTR stratified by post-ACT ctDNA status; censored patients are indicated with a vertical line. **b** Proportion of patients at risk of recurrence after 3 years. **c** Kaplan–Meier estimate for TTR stratified by ctDNA dynamics based on post-surgery and post-ACT ctDNA status; censored patients are indicated with a vertical line. **d** Aggregate ctDNA VAF on a logarithmic scale for each combination of post-surgery—post-ACT ctDNA test results. ACT, adjuvant chemotherapy; ctDNA, circulating tumour DNA; TTR, time to recurrence; VAF, variant allele fraction; LOD, limit of detection.

After adjuvant treatment, post-ACT ctDNA could be investigated in 171 of the 209 patients (*Fig. S6*). Twenty-four patients were ctDNA positive after ACT, which was associated with a poor TTR compared with ctDNA-negative patients after ACT (HR 7.9 (95% c.i. 3.9 to 15.9); *P* < 0.001) (*Fig. 2a*,*b*). Dynamic changes in ctDNA status (‘negative’ or ‘positive’) before and after ACT were evaluated by combining post-surgery and post-ACT ctDNA results (*Fig. 2c*). All patients in the ctDNA positive-positive group (7 patients) experienced disease recurrence, indicating that treatment with ACT did not succeed in eliminating disease. In contrast, the 3-year recurrence risk of the ctDNA negative-negative group (137 patients) was 10.6%. Ten of the 17 patients (58.8%) who were ctDNA positive post-surgery became ctDNA negative post-ACT (positive-negative) and had a risk of recurrence that was not different from the ctDNA negative-negative group (Cox regression, *P* = 0.074), suggesting benefit from ACT. The ctDNA negative-positive group had an intermediate risk of recurrence, with an increased risk of disease recurrence when compared with the ctDNA negative-negative group (Cox regression, *P* < 0.001). Notably, ctDNA levels in the majority of patients in the ctDNA positive-negative and negative-positive groups were close to the limit of detection of the ctDNA test and lower than the ctDNA levels observed in the ctDNA positive-positive patients (*Fig. 2d*). The highest ctDNA levels (>0.10%) corresponded to patients experiencing a recurrence.

### Post-ACT ctDNA surveillance for early detection of recurrences

**Fig. 3:**
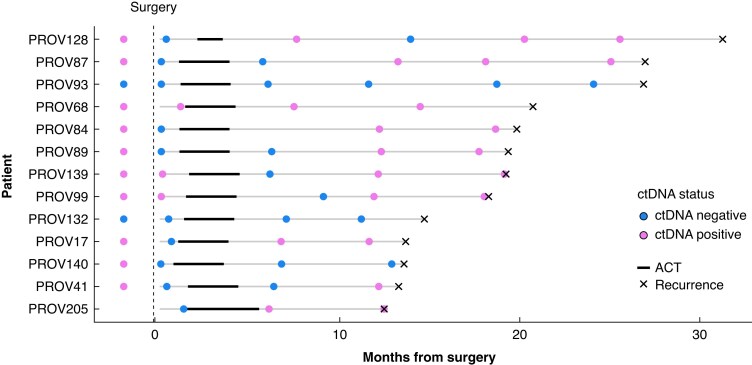
Swimmer plot of all plasma samples evaluated for the 13 patients who experienced a recurrence and for whom more than one post-ACT sample was available ACT, adjuvant chemotherapy; ctDNA, circulating tumour DNA.

Longitudinal ctDNA analyses were performed for the 13 patients who experienced a recurrence and had more than one post-ACT blood collection time point available (*Fig. 3*). Three of these 13 patients were ctDNA positive immediately post-surgery. One of these three patients remained ctDNA positive at all longitudinal time points evaluated, while, in the other two patients, ctDNA was not present in the first blood sample collected after completion of ACT but became detectable again at the second time point evaluated after ACT. Seven of the ten post-surgery ctDNA-negative patients became ctDNA positive during longitudinal follow-up. Three of the 13 patients were ctDNA negative for all of the longitudinal samples evaluated post-surgery, of whom 2 were also ctDNA negative pre-surgery. These three patients developed metastases in the peritoneum, the intra-abdominal lymph nodes, and a local recurrence, respectively. Taken together, these data demonstrate a clinical sensitivity for recurrence detection before clinical recurrence of 77% (10 of 13) in the surveillance setting, with molecular recurrence detected for the first time (that is first ctDNA-positive sample during follow-up) at a median of 7.6 months before recurrence (minimum = 33 days, maximum = 412 days).

## Discussion

The PROVENC3 study demonstrated that the risk of recurrence for patients with stage III colon cancer can be better predicted by performing post-surgery ctDNA testing, using a highly sensitive tumour-informed WGS ctDNA test. The prognostic value is further increased by combining post-surgery ctDNA status with pathological risk, which reveals a large subgroup (more than 50%) of low-risk ctDNA-negative patients who had a very low 3-year disease recurrence risk of 6.7%.

This study also described a novel tumour-informed WGS ctDNA test with robust analytical and clinical sensitivities, illustrated by the high percentage of patients with detectable ctDNA pre-surgery, compared with, for example, a recently published WGS-based ctDNA detection method with 84% sensitivity pre-surgery in a similar cohort of stage III CRC patients^20^ and previously published methods based on next-generation sequencing^21^. Nevertheless, 8.8% of cancers remained undetected pre-surgery, which could be due to preanalytical or analytical factors, but also to variability in ctDNA-shedding biological characteristics among primary tumours. Such variability in ctDNA-shedding properties appears more evident when evaluating the different locations of the metastatic lesions. For example, none of the patients who developed peritoneum-only metastases had detectable ctDNA immediately post-surgery in this study. This observation is in agreement with reports in the literature indicating that peritoneal metastases are difficult to detect by ctDNA testing^22,23^. Therefore, while ctDNA assays are further developed with increasing analytical sensitivity, it remains important to identify the molecular and physiological features that define a high or low likelihood of detectable levels of ctDNA in blood, as ctDNA-based strategies may not be suited for recurrence-risk stratification of the subset of ‘low shedding cancers’.

The clinical sensitivity of ctDNA to detect disease recurrence in the PROVENC3 study, both at the landmark post-surgery time point and longitudinally, is consistent with recent data indicating that ctDNA is detectable typically 6–10 months before a recurrence becomes clinically overt^8^. Post-surgery ctDNA testing holds promise to tailor ACT, specifically, to guide ACT escalation decisions by identifying the patients at (very) high risk of developing a recurrence, either at the MRD time point or after ACT. On the other hand, post-surgery ctDNA could guide de-escalation as well for a group of patients at very low risk of recurrence. However, to further advance robust de-escalation strategies, more data regarding ctDNA status for patients who do not receive ACT are needed.

A considerable proportion of patients who were ctDNA negative post-surgery developed recurrences, which indicates that ctDNA status alone is currently insufficient to routinely withhold chemotherapy. Importantly, longitudinal WGS-based ctDNA testing detected ctDNA in the majority of the patients evaluated, indicating that appropriate surveillance strategies need to be developed to improve disease management. ctDNA-based surveillance may provide a window of opportunity for earlier and potentially more effective intervention than is possible when the recurrence manifests clinically or on imaging. These early interventions may constitute local treatment with curative intent, including surgical resection or radiotherapy for localized recurrences, or systemic treatment for wider metastatic spread or even in the absence of macroscopic disease. However, the lead time of ctDNA testing to detect molecular recurrence *versus* CT, the need of confirmatory imaging procedures after a positive ctDNA test during surveillance, and the optimal frequency of longitudinal ctDNA testing remain to be systematically assessed, and longer follow-up in current and future studies is needed to evaluate whether intensified follow-up with early treatment leads to improved overall survival. Importantly, this will require a shift in mindset from the decades-old dogma of ‘one-size-fits-all’ treatment with ACT to more personalized ctDNA-guided ACT decision-making and interventions.

The clinical performance of the ctDNA test employed in the PROVENC3 study is similar to the results of tumour-informed bespoke panel-based approaches, including the GALAXY study in Japan and the DYNAMIC study in Australia (14.8% and 15.3% of patients harboured detectable ctDNA post-surgery respectively)^5,9^. Compared with the approaches used in those studies, the present study demonstrated that a WGS-based technique can achieve a similar clinical sensitivity to patient-specific bespoke panel-based methods, without the need to design, manufacture, and quality-control custom panels for each patient. This results in a significantly shorter turnaround time (within 14 days *versus* 4–6 weeks from sample to report), which is especially relevant in the post-surgical setting, where ACT should preferably be initiated within 8 weeks post-surgery.

Each ctDNA detection assay has an analytical limit of detection, representing the lowest amount of ctDNA in blood that can be detected. At present, this limit of detection needs to be improved further to increase the clinical sensitivity for ctDNA detection and to detect a higher proportion of patients with occult disease. Technically, this may be achieved by including evaluation of additional tumour-specific cell-free DNA features together with increases in the depth of sequencing and reductions in technical background error rates^24,25^. Clinically, when ctDNA levels are reported that are close to the limit of detection, the results may be influenced by stochastic variation in blood sampling and should be interpreted with caution. This is illustrated by the evaluation in the present study of ctDNA dynamics in response to treatment with ACT: two patients who cleared ctDNA during ACT but experienced a recurrence had an aggregate ctDNA VAF close to the limit of detection post-surgery and then became ctDNA positive quickly during longitudinal surveillance. This phenomenon represents a limitation in the current state of ctDNA technologies to detect MRD, where variation in ctDNA status at very low ctDNA levels may occur due to a reduction or increase in overall tumour burden or may occur due to stochastic variation in ctDNA sampling.

Current stratification for adjuvant treatment in non-metastatic colon cancer is based on lymph node status. Compared with the current standard of care, post-surgery ctDNA testing offers a substantial improvement, as it enables the identification of patients with MRD who are highly likely to develop a recurrence if left untreated (in >95% of cases). At the same time, ctDNA-negative patients have a reduced risk of developing a recurrence. The main challenge in the MRD setting is that the ctDNA levels secreted by the few tumour cells still present in the body are very low and even the most sensitive techniques will miss a number of cases (false negatives). Importantly, the risk stratification presented here in the PROVENC3 study illustrates the added value of the combination of post-surgery ctDNA testing and pathological features, which allowed the identification of a substantial group of patients with a recurrence risk of only 6.7% who might benefit from ACT being withheld. Interventional studies are needed to demonstrate the clinical utility of the combination of ctDNA testing with pathological risk stratification to guide ACT decisions.

Recent studies have shown the potential benefit of using neoadjuvant therapy for non-metastatic colon cancer patients: chemotherapy for stage T4b + microsatellite stability (MSS)^26^, immunotherapy for MSI^27^, and potentially a combination of both for stage T3–4 + MSS + *RAS*/*RAF* wild type^28^. The implementation of neoadjuvant treatment for subgroups of stage III colon cancer patients could have implications for ctDNA testing strategies. Post-surgery detection of ctDNA in patients who are treated with neoadjuvant therapy will be even more challenging than its detection in treatment-naïve patients, due to the reduction in the number of cancer cells in micrometastatic lesions by the neoadjuvant treatment. Pre-surgery, ctDNA testing could be considered as a tool to stratify patients for neoadjuvant treatment or to evaluate the response to neoadjuvant treatment in a minimally invasive manner. The clinical applicability and utility of ctDNA testing in the setting of neoadjuvant treatment would need to be carefully evaluated in prospective observational and interventional clinical trials.

Limitations of this study included lack of overall survival data in addition to TTR data, the focus on a single post-surgery landmark ctDNA time point, variation in timing of the post-surgery blood sample collection, heterogeneity in histopathological subtypes, quality-control failure in 19 of 241 cases, and the fact that preoperative samples and post-ACT samples could not be collected for all patients.

## Collaborators

M. Los (St. Antonius Ziekenhuis, Nieuwegein, The Netherlands); J. M. van Dodewaard (Meander Medisch Centrum, Amersfoort, The Netherlands); M. L. R. Tjin-A-Ton (Ziekenhuis Rivierenland, Tiel, The Netherlands); A. H. W. Schiphorst (Diakonessenhuis Utrecht, Utrecht, The Netherlands); M. Koopman (University Medical Centre Utrecht, Utrecht, The Netherlands), D. W. Sommeijer (Flevoziekenhuis, Almere, The Netherlands; Academisch Medisch Centrum Amsterdam, The Netherlands); P. J. Tanis (Academisch Medisch Centrum Amsterdam, The Netherlands); K. Talsma (Deventer ziekenhuis, Deventer, The Netherlands); H. B. A. C. Stockmann (Spaarne Gasthuis, Haarlem, The Netherlands); D. D. E. Zimmerman (Elisabeth-TweeSteden Ziekenhuis, Tilburg, The Netherlands); W. J. Vles (Ikazia Ziekenhuis, Rotterdam, The Netherlands); L. B. J. Valkenburg (Maastricht University Medical Centre, Maastricht, The Netherlands); R. C. Rietbroek (Rode Kruis Ziekenhuis, Beverwijk, The Netherlands); E. J. Th. Belt (Albert Schweizer Ziekenhuis, Dordrecht, The Netherlands); J. W. T. Dekker (Reinier de Graaf Gasthuis, Delft, The Netherlands); I. H. J. T. de Hingh (Catharina Ziekenhuis, Eindhoven, The Netherlands); R. W. M. Schrauwen (Bernhoven, Uden, The Netherlands); F. J. F. Jeurissen (Haaglanden MC, Den Haag, The Netherlands); R. Hoekstra (Ziekenhuis Groep Twente, Almelo, The Netherlands); M. P. Hendriks (Noordwest Ziekenhuisgroep, Alkmaar, The Netherlands); J. Jansen (Admiraal de Ruyter Ziekenhuis, Goes, The Netherlands); J. Konsten (VieCuri MC, Venlo, The Netherlands); P. Nieboer (Wilhelmina Ziekenhuis Assen, Assen, The Netherlands); N. A. J. B. Peters (St. Jans Gasthuis, Weert, The Netherlands); J. F. M. Pruijt (Jeroen Bosch Ziekenhuis, s-Hertogenbosch, The Netherlands); L. Simkens (Maxima Medisch Centrum, Veldhoven, The Netherlands); A. I. de Vos (Van Weel-Bethesda Ziekenhuis, Middelharnis, The Netherlands); A. Schouten van der Velden (Ziekenhuis St. Jansdal, Harderwijk, The Netherlands); A. W. Haringhuizen (Ziekenhuis Gelderse Vallei, Ede, The Netherlands); M. A. Davidis (Rivas, Gorinchem, The Netherlands); J. Janssen (Canisius Wilhelmina Ziekenhuis, Nijmegen, The Netherlands).

## Supplementary Material

znaf281_Supplementary_Data

## Data Availability

The main data associated with this study are provided in the main text and *Supplementary material*. Raw whole genome sequencing data are deposited in EGAS50000000804 and subject to controlled access via a Data Access Committee and Data Transfer Agreement, conforming to informed consent and GDPR regulations.
