# Supplementary material for: Circulating tumour DNA in patients with stage III colon cancer: multicentre prospective PROVENC3 study
Source: Br J Surg. 2026 Jan 9;113(1):znaf281. doi: 10.1093/bjs/znaf281 (PMC12785881; doi:10.1093/bjs/znaf281)
Supplement: znaf281_Supplementary_Data [file znaf281_supplementary_data.docx]

**Title:**

**Circulating tumour DNA (ctDNA) in patients with stage III colon cancer: the multicentre prospective PROVENC3 study**

**Authors:**

Carmen Rubio-Alarcón^1^, Andrew Georgiadis^2,11^, Ingrid A. Franken^3,11^, Haoyue Wang^4^, Sietske C.M.W. van Nassau^3^, Suzanna J. Schraa^3^, Dave E.W. van der Kruijssen^3^, Karlijn van Rooijen^3^, Theodora C. Linders^5^, Pien Delis-van Diemen^1^, Maartje Alkemade^6^, Anne Bolijn^1^, Marianne Tijssen^1^, Margriet Lemmens^1^, Lana Meiqari^1^, Steven L.C. Ketelaars^1^, Adria Closa-Mosquera^1^, Miranda M.W. van Dongen^1^, Mirthe Lanfermeijer^5^, Birgit I. Lissenberg-Witte^4^, Linda J.W. Bosch^1^, Teunise Bisschop-Snetselaar^3^, Bregje C. Adriaans^3^, Amy Greer^2^, David Riley^2^, James R. White^2^, Christopher Greco^2^, Liam Cox^2^, Jesse Fox^2^, Kaitlin Victor^2^, Catherine Leech^2^, Samuel V. Angiuoli^2^, Niels F.M. Kok^7^, Cornelis J.A. Punt^8^, Daan van den Broek^5^, Miriam Koopman^3^, Gerrit A. Meijer^1^, Victor E. Velculescu^9^, Jeanine M. L. Roodhart^3^, Veerle M.H. Coupé^4^, Mark Sausen^2^, Geraldine R. Vink^3,10^, Remond J.A. Fijneman^1^* on behalf of the PLCRC-MEDOCC group

^1^The Netherlands Cancer Institute, Department of Pathology, Amsterdam, The Netherlands.

^2^Labcorp, Baltimore, MD, USA.

^3^ Department of Medical Oncology, University Medical Center Utrecht, Utrecht University, Utrecht, The Netherlands.

^4^Amsterdam University Medical Centers, Location VU Medical Center, Department of Epidemiology and Data Science, Amsterdam, The Netherlands.

^5^The Netherlands Cancer Institute, Department of Laboratory Medicine, Amsterdam, The Netherlands.

^6^Core Facility Molecular Pathology and Biobanking (CFMPB), The Netherlands Cancer Institute, Amsterdam, The Netherlands.

^7^Department of Surgical Oncology, The Netherlands Cancer Institute, Plesmanlaan 121, 1066 CX, Amsterdam, The Netherlands.

^8^Department of Epidemiology, Julius Center, University Medical Center Utrecht, Utrecht University, Utrecht, The Netherlands

^9^The Sidney Kimmel Comprehensive Cancer Center, Johns Hopkins University School of Medicine, Baltimore, MD , USA.

^10^Department of Research and Development, Netherlands Comprehensive Cancer Organisation, Utrecht, The Netherlands.

^11^These authors contributed equally

***Corresponding author**:

Remond J.A. Fijneman, Plesmanlaan 121, 1066CX Amsterdam, the Netherlands; Email: r.fijneman@nki.nl; Phone: (+31) 20 512 9111; ORCID ID: 0000-0003-2076-5521

**Supplementary Materials - Index**

| **Supplementary Methods** |  |
| --- | --- |
| Sample collection and processing | *pag. 3* |
| Labcorp® Plasma Detect™ ctDNA test methodology | *pag. 3* |
| **Supplementary Results** |  |
| Labcorp Plasma Detect ctDNA test analytical performance | *pag. 7* |
| **Supplementary Figures and tables** |  |
| Supplementary Figure 1 | *pag. 9* |
| Supplementary Figure 2 | *pag. 11* |
| Supplementary Figure 3 | *pag. 12* |
| Supplementary Figure 4 | *pag. 14* |
| Supplementary Figure 5 | *pag. 15* |
| Supplementary Figure 6 | *pag. 16* |
| Supplementary Table 1 | *pag. 18* |
| Supplementary Table 2 | *pag. 20* |
| Supplementary Table 3 | *pag. 22* |
| Supplementary Table 4 | *pag. 30* |
| Supplementary Table 5 | *pag. 44* |
| Supplementary Table 6 | *pag. 62* |
| Supplementary Table 7 | *pag. 63* |
| Supplementary Table 8 | *pag.64* |
| Supplementary Table 9 | *pag. 65* |
| **References** | *pag. 66* |
|  |  |

**Supplementary Methods**

***Sample collection and processing***

DNA was isolated from FFPE slides using the QIAGEN AllPrep DNA/RNA FFPE kit (QIAGEN, Hilden, Germany) and stored at -20°C or 4°C only for short term before shipment. DNA quality and quantity were measured on a Nanodrop One (Isogen, Ijsselstein, The Netherlands) and on a Qubit 3.0 Fluorometer (Molecular Probes, Leiden, The Netherlands) with the use of the Qubit dsDNA High-Sensitivity Assay (Thermo Fisher Scientific, USA).

Cell-free plasma and white blood cells (WBC) were separated by centrifugation of the blood for 10 minutes at 1,700xg followed by 10 minutes at 20,000xg, then stored at -80°C until further processing. Cell-free DNA (cfDNA) was isolated from the available plasma using the QIAsymphony DSP Circulating DNA Kit (QIAGEN, Hilden, Germany) with a fixed elution volume of 60 µL. Genomic DNA was isolated from WBCs using the QIAsymphony DSP DNA Midi Kit (QIAGEN, Hilden, Germany) and 1 ml blood protocol. cfDNA and genomic DNA from WBCs was stored at -20°C until further processing. The Qubit dsDNA High-Sensitivity Assay (Thermo Fisher, Waltham, USA) was used to quantify DNA yield for next generation sequencing.

***Labcorp Plasma Detect ctDNA test methodology***

Labcorp Plasma Detect is a novel tumour-informed WGS-based plasma ctDNA test for detection of minimal residual disease after curative intent intervention, including surgery or adjuvant chemotherapy. We here provide a detailed description of the Labcorp Plasma Detect ctDNA test methodology and associated analytical test performance.

*Noncancerous donor plasma and commercial cell line cohorts*

Noncancerous donor plasma samples were obtained under Institutional Review Board approval from Discovery Life Sciences (Alabama, USA). Human tumour and normal cells from previously characterised cell lines were obtained from ATCC (Virginia, USA) (COLO-829, HCC-1187, HCC-1143, HCC-1954) and SeraCare (Massachusetts, USA) (SeraSeq gDNA TMB-mix Score 26). Clinical samples collected under IRB-approved protocols from nine colorectal cancer patients (SU8790, SU8792, SU8794, SU8799, SU8810, SU8820, SU8830, SU8836, SU8837) and five head and neck patients (SU8804, SU8809, SU8821, SU8825, SU8842) were commercially procured. cfDNA was isolated from plasma using the Qiagen Circulating Nucleic Acid kit (Qiagen, Germany) and the concentration was assessed using the Qubit dsDNA High-Sensitivity Assay (Thermo Fisher, USA). Genomic DNA was isolated from cell line samples using the QIAamp DNA Blood Mini Kit (Qiagen, Germany) and the concentration assessed using the Qubit dsDNA Broad Range Assay (Thermo Fisher, USA).

*WGS analysis of tissue-derived tumour DNA and WBC-derived germline DNA*

White blood cell (WBC)-derived genomic DNA was quantified using the Qubit dsDNA Broad Range Assay (Thermo Fisher, USA) and up to 400 ng of DNA was sheared to a target fragment size of approximately 450 base pairs (bp) using Covaris focused ultrasonication (Covaris, USA). Additionally, genomic DNA derived from FFPE tumour tissue was repaired using the PreCR Repair Mix (New England Biolabs, USA). Whole-genome sequencing libraries were prepared from fragmented genomic DNA through end-repair, A-tailing, and adapter ligation with the KAPA HyperPrep reagent kit according to the manufacturer’s protocol (Roche, USA). Subsequently, these libraries were amplified through 7 cycles of polymerase chain reaction (PCR), pooled, and sequenced with 150 bp paired-end reads using the Illumina NovaSeq6000 platform (Illumina, USA) to a target depth of 80x for tumour samples and 40x for germline samples. After demultiplexing was performed using bcl2fastq (Illumina, USA), FASTQ files were aligned to the GRCh38 human reference genome using BWA-MEM (v0.7.15). PCR duplicates were marked using Novosort (v1.03.01) and base quality score recalibration was performed using GATK BQSR (v4.1.0). The aligned BAM files were subjected to single nucleotide variant (SNV) analyses using MuTect2 (GATK v4.0.5.1), Strelka2 (v2.9.3), and Lancet (v1.0.7). SNVs were annotated as high confidence if they were reported by at least two variant callers^[1]^ .

*WGS analysis of plasma-derived cell-free DNA and contrived DNA*

Plasma cfDNA and contrived DNA obtained from fragmented matched tumour and germline cell lines were quantified using the Qubit dsDNA High-Sensitivity Assay (Thermo Fisher, USA). Whole-genome sequencing libraries were prepared from plasma cell-free DNA or contrived DNA using a target of 10 ng of DNA through end-repair, A-tailing, and adapter ligation with custom molecular barcoded adapters^[2]^. Subsequently, these libraries were amplified through 5 cycles of PCR, pooled, and sequenced with 150 bp paired-end reads using the Illumina NovaSeq6000 platform (Illumina, USA) to a target depth of 30x. After demultiplexing was performed, FASTQ files were quality trimmed using Trimmomatic (v0.33) and aligned to the hg19 human reference genome using BWA-MEM2 (v2.2.1). Somatic variant identification was performed using VariantDx (v11.0.0), which has demonstrated high accuracy for somatic mutation detection and differentiating technical artifacts to enable analyses of SNVs^[3,4]^.

*Detection of ctDNA through integrated WGS analysis*

Initially, to ensure that the tumour, germline, and plasma WGS datasets were derived from the same subject, an analysis was performed across 10,000 common single nucleotide polymorphisms. Then, a quality control analysis was performed using Picard (v2.18.14) and required ≥20x sequencing depth with a median insert size ≥150 bp for cfDNA samples, ≥40x sequencing depth for tumour samples, and ≥20x sequencing depth for germline samples. Tumour-specific SNVs were filtered to a candidate somatic mutation set by removing: (1) variants observed in the 1000 Genomes (Phase 3) or gnomAD (r2.0.1) population databases, (2) variants overlapping the hg19 UCSC simple tandem repeats track, (3) positions with <10x depth in the tumour or matched normal, (4) positions with an alternate allele count <4 in the tumour or >1 in the matched germline, and (5) variants with a tumour variant allele frequency (VAF) <0.05 (more strict filtering was applied to T>C/A>G variants, which were removed if the tumour VAF was <0.20 or the alternate allele count was <10). Additional variant filtering was performed through generation of a blacklist, where variants were further removed if present (1) in >10% of noncancerous donors or (2) any non-cancerous donor contained the variant with ≥25% VAF across a cohort of 20 noncancerous donor plasma samples evaluated in quadruplicate. The final candidate tumour-specific variant set was then compared to the matched test sample unfiltered variant results. Candidate tumour-specific SNVs identified in the test sample were scored (ranging from 0 to 1) using a random forest machine learning algorithm trained using the *caret* package (v6.0.90) within the R statistical computing environment (v4.1.1), independently of the PROVENC3 cohort. To avoid overfitting, model training utilised 5-fold cross validation and limited the number of selected variables per split procedure (hyperparameter *mtry*) to the square-root of the total number of input features. Variants present in properly paired mapped fragments with a random forest score >0.25 were further assessed, requiring an alternate read mapping quality ≥30 and a read-based mutation rate ≤5. The individual variant random forest scores were then aggregated and normalised based on the total number of tumour-specific SNVs assessed. The normalised random forest score (NRFS) was then compared to the noncancerous donor cohort, and a cutoff of one standard deviation above the maximum observed NRFS was required to report an individual test sample as having evidence of the tumour-specific variants. An estimated tumour fraction (termed “Aggregate ctDNA VAF”) was then calculated for each positive test sample based on the aggregate variant allele observations observed as a proportion of the total unique coverage of all individual tumour-specific variants assessed.

**Supplementary Results**

***Labcorp Plasma Detect ctDNA test analytical performance***

The Labcorp Plasma Detect ctDNA assay involves integrated WGS analyses of patient-matched FFPE tumour tissue DNA, WBC-derived germline DNA and plasma cfDNA (Supplementary Figure 2). Analytical sensitivity studies were performed using contrived reference models derived from five commercially available cell lines, including lung cancer (n=1), breast cancer (n=3), and melanoma (n=1); and commercially procured clinical samples, including nine colorectal cancer and five head and neck cancer patients. Contrived samples were generated from three cell lines (COLO-829, HCC-1187, and HCC-1143) and evaluated in triplicate at 10%, 1%, 0.10%, 0.05%, 0.02%, 0.01%, 0.005%, and 0.001% tumour content. An additional contrived sample series (HCC-1187, HCC-1954) was generated and evaluated in triplicate at 0.05%, 0.01%, 0.005%, and 0.001% tumour content, along with the external contrived reference control sample (SeraSeq gDNA TMB-mix Score 26) evaluated at 0.05% (n=7), 0.01% (n=2), 0.005% (n=3), and 0.001% (n=3) tumour content to increase the number of datapoints near the expected limit of detection. Through a regression analysis of these data, a 95% limit of detection of 0.005% tumour content and a 50% limit of detection of 0.001% tumour content was demonstrated (Supplementary Figure 2b; Supplementary Tables 2 and 3). Analytical specificity studies demonstrated a specificity of 99.6% (2,015/2,023) across 119 noncancerous donor plasma specimens evaluated against 17 reference whole-genome somatic mutation datasets (Supplementary Figure 2c; Supplementary Tables 2 and 3). Analysis of an external contrived reference control sample (SeraSeq gDNA TMB-mix Score 26; 0.05% tumour content) demonstrated highly reproducible results for the estimated tumour fraction across 45 independent sequencing runs evaluated for the PROVENC3 clinical study cohort (n=45 runs, coefficient of variation (CV) = 7.2%; Supplementary Figure 2d). A median of 5,108 (IQR 3,776-7,411) high confidence tumour-specific single nucleotide variants were identified per patient in the PROVENC3 cohort, consistent with previous studies^[5,6]^, which were utilised for plasma ctDNA detection (Supplementary Table 4).

**Supplementary Figure 1**


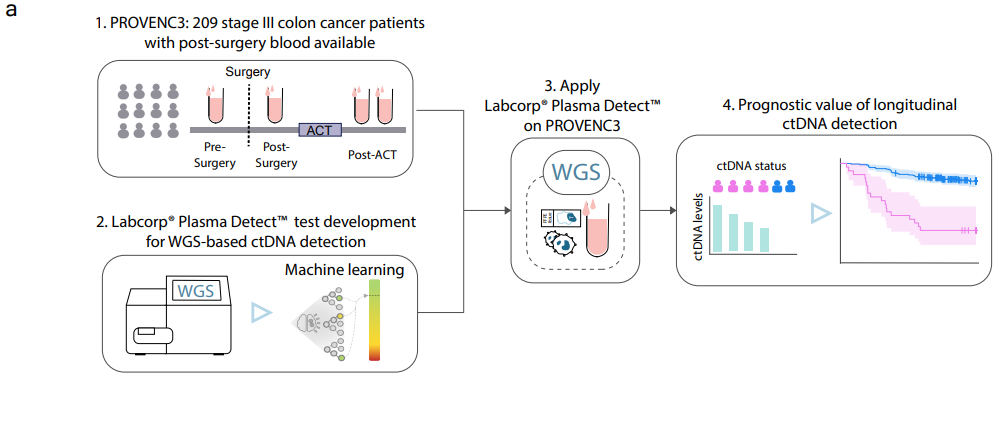

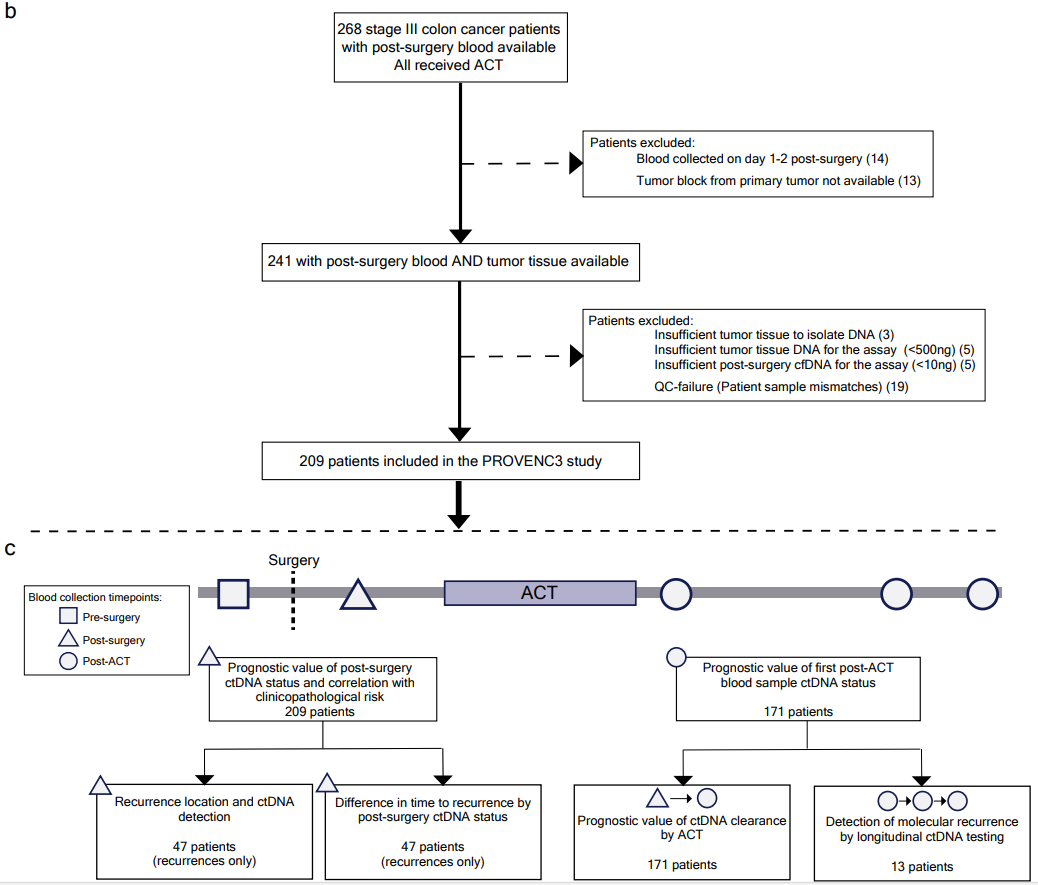


**Supplementary Figure 1**. PROVENC3 study overview. a) Study overview. b) Selection of the 209 patients of the PROVENC3 clinical study population and the main exclusion criteria from final analysis. c) Overview of the number of patients analysed for each research question. Each rectangle contains a research question evaluated, and the number of patients and ctDNA time points evaluated. ACT, adjuvant chemotherapy; WGS, whole genome sequencing; QC, quality control; FU, follow up.

**Supplementary Figure 2**


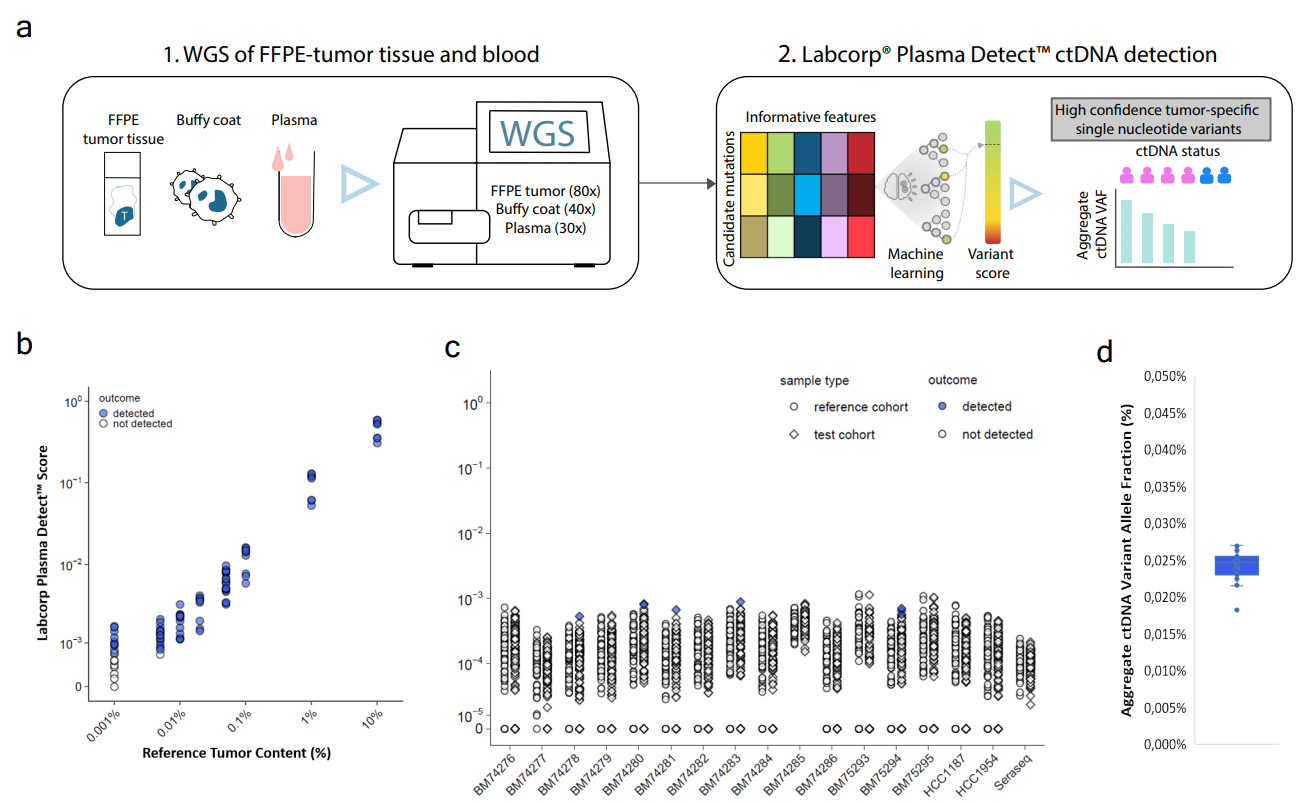


**Supplementary Figure 2.** a) Schematic overview of the Labcorp Plasma Detect test workflow. b) Analytical sensitivity studies were conducted across five commercially available contrived cell line samples at 10%, 1%, 0.10%, 0.05%, 0.02%, 0.01%, 0.005%, and 0.001% tumour content. c) Analytical specificity was evaluated across 119 noncancerous donor plasma specimens against 17 reference whole genome somatic mutation datasets. d) Reproducibility analysis of an external contrived reference control sample (0.05% tumour content) across 45 independent runs evaluated for the PROVENC3 clinical study cohort (n=45 runs, CV = 7.2%).

**Supplementary Figure 3**


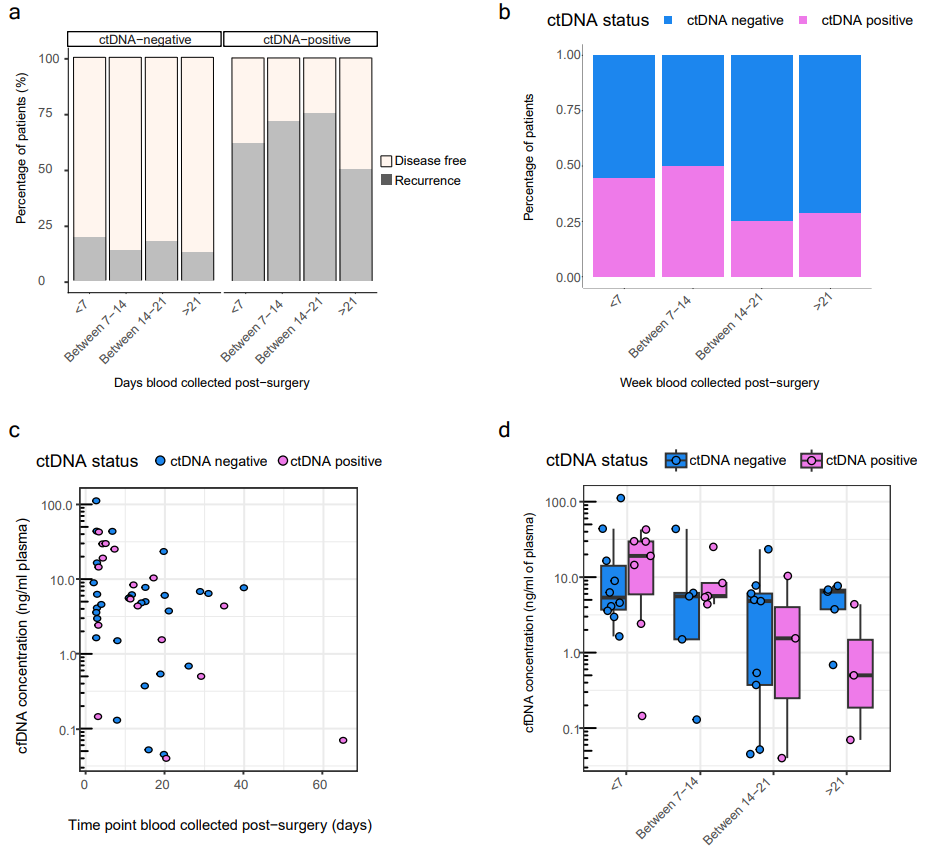


**Supplementary Figure 3.** Post-surgery ctDNA status and cfDNA concentration in context of timing of the landmark post-surgery blood draw. a) Percentage of patients experiencing a recurrence per time window of post-surgery blood draw, stratified by ctDNA status post-surgery (n=209). b) Percentage of the 47 patients experiencing a recurrence per time window of post-surgery blood draw. c) Overview of cfDNA concentration (ng/mL of plasma) compared across different time windows for 47 patients who experienced a recurrence. d) cfDNA concentration stratified by ctDNA status across different time windows for 47 patients who experienced a recurrence. cfDNA, cell free DNA; ng, nanograms; mL, milliliter.

**Supplementary Figure 4**


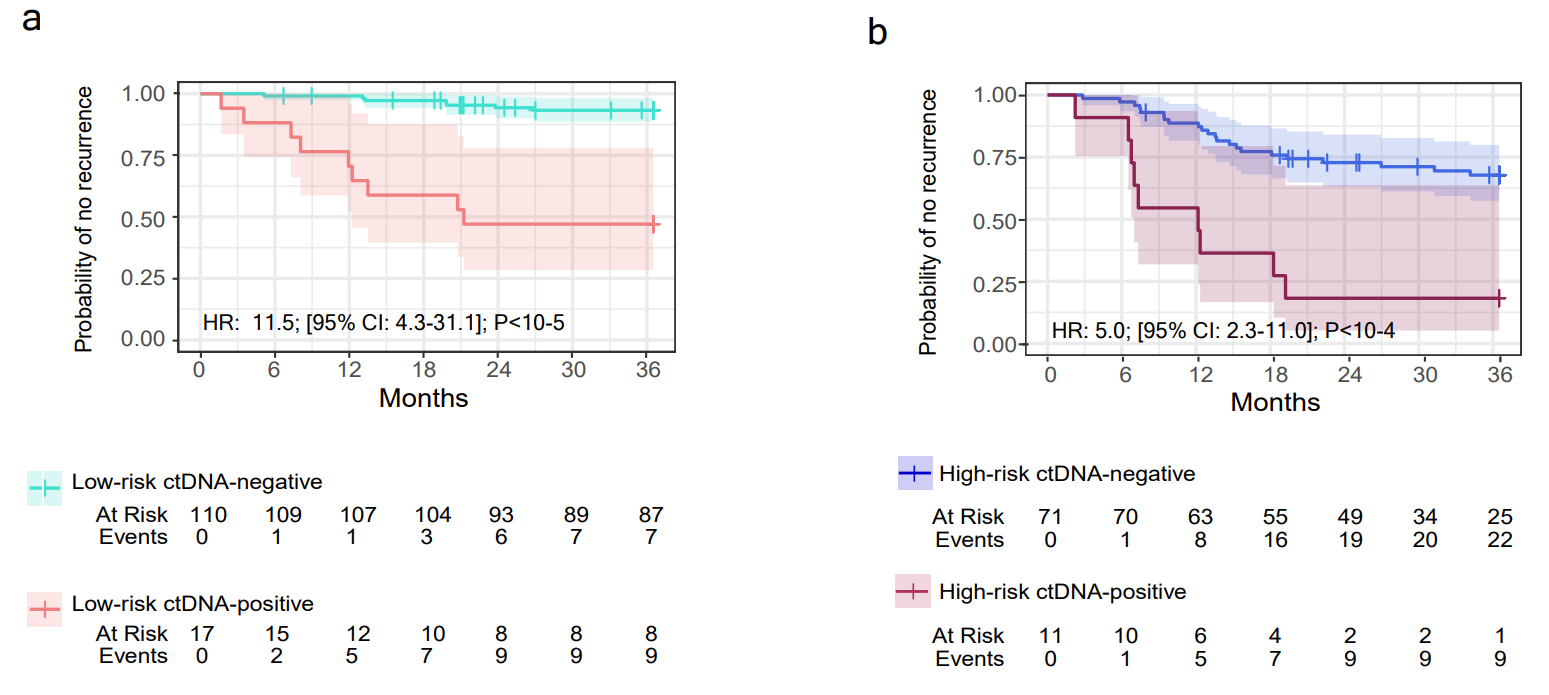


**Supplementary Figure 4.** TTR for pathological low-risk and high-risk groups, stratified by post-surgery ctDNA status. a) Kaplan-Meier estimate for cox regression analyses for pathological low-risk stage III colon cancer patients stratified by post-surgery ctDNA status, including confidence intervals. Censored patients are indicated with a vertical line. b) Kaplan-Meier estimate for cox regression analyses for pathological high-risk stage III colon cancer patients stratified by post-surgery ctDNA status, including confidence intervals. Censored patients are indicated with a vertical line.

**Supplementary Figure 5**


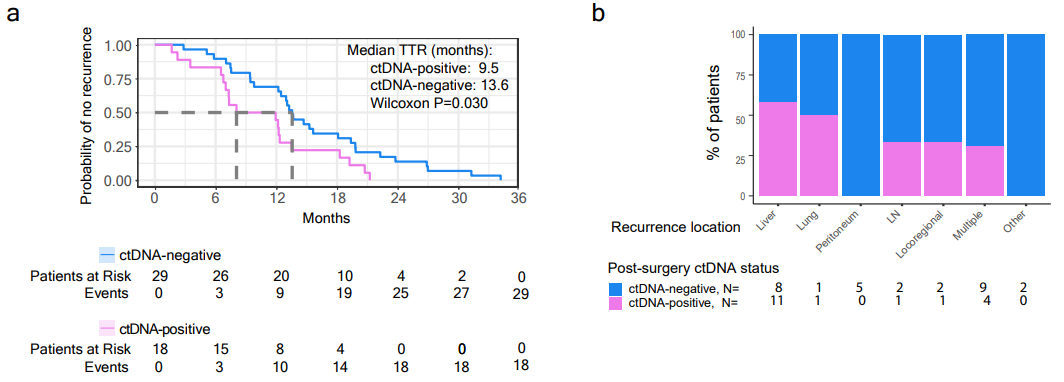


**Supplementary Figure 5.** Post-surgery ctDNA status in patients experiencing a recurrence. a) Kaplan-Meier estimate for time to recurrence, stratified by post-surgery ctDNA status for all patients experiencing disease recurrence (n=47). b) Post-surgery ctDNA status per recurrence location (n=47). ctDNA, circulating tumour DNA; ACT, adjuvant chemotherapy; TTR, time to recurrence.

**Supplementary Figure 6** ­­­­­­

­­
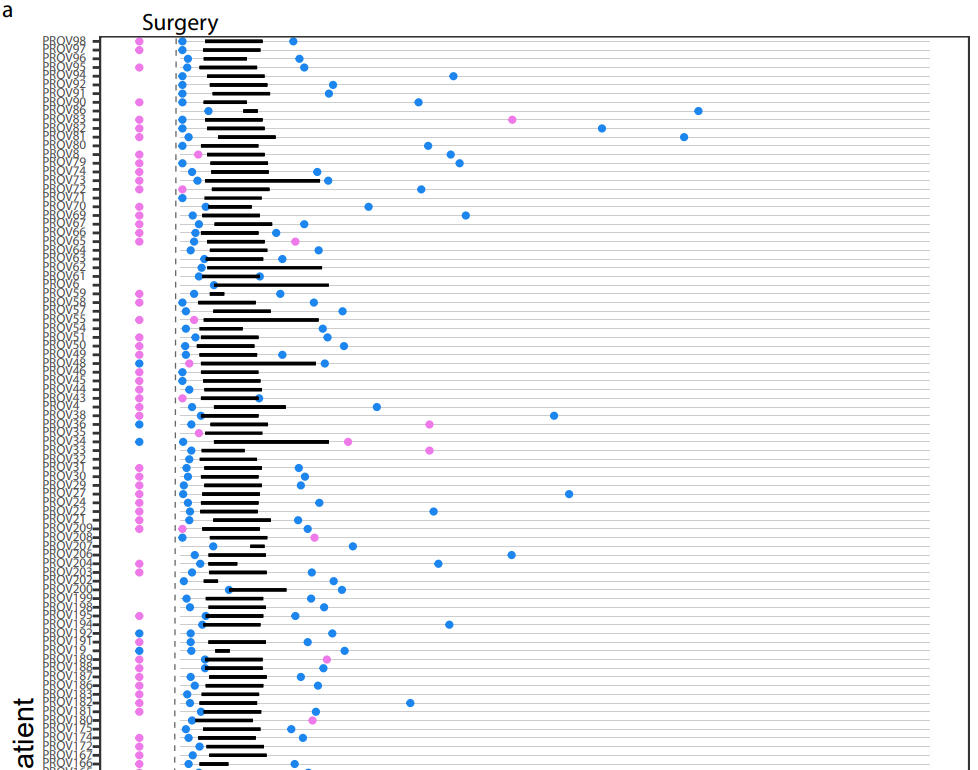


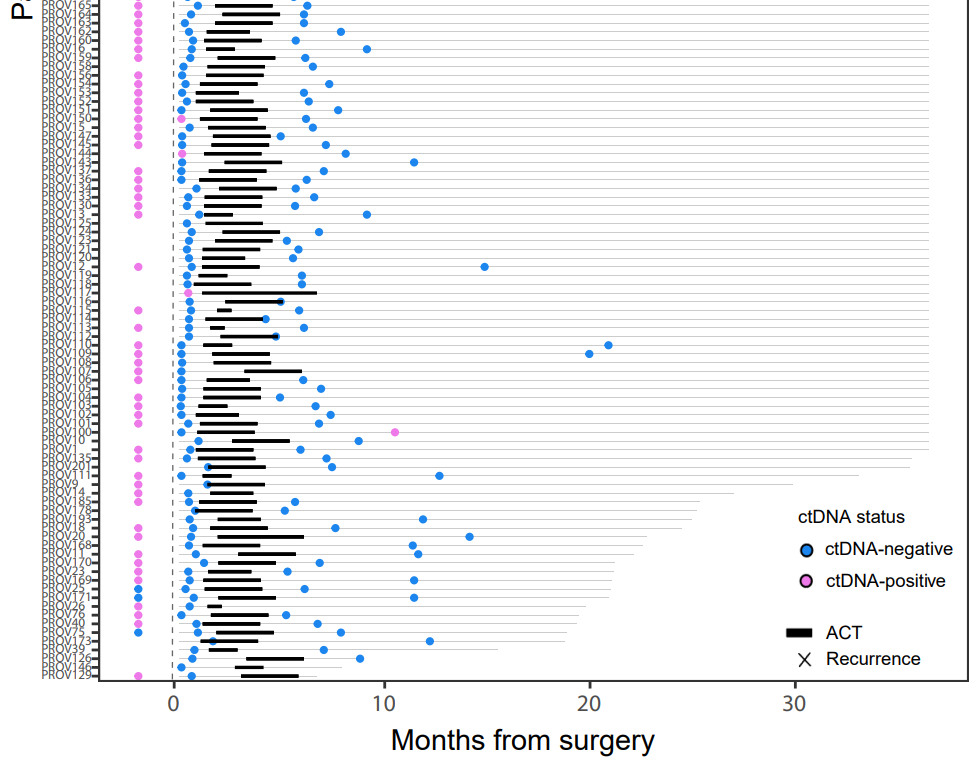


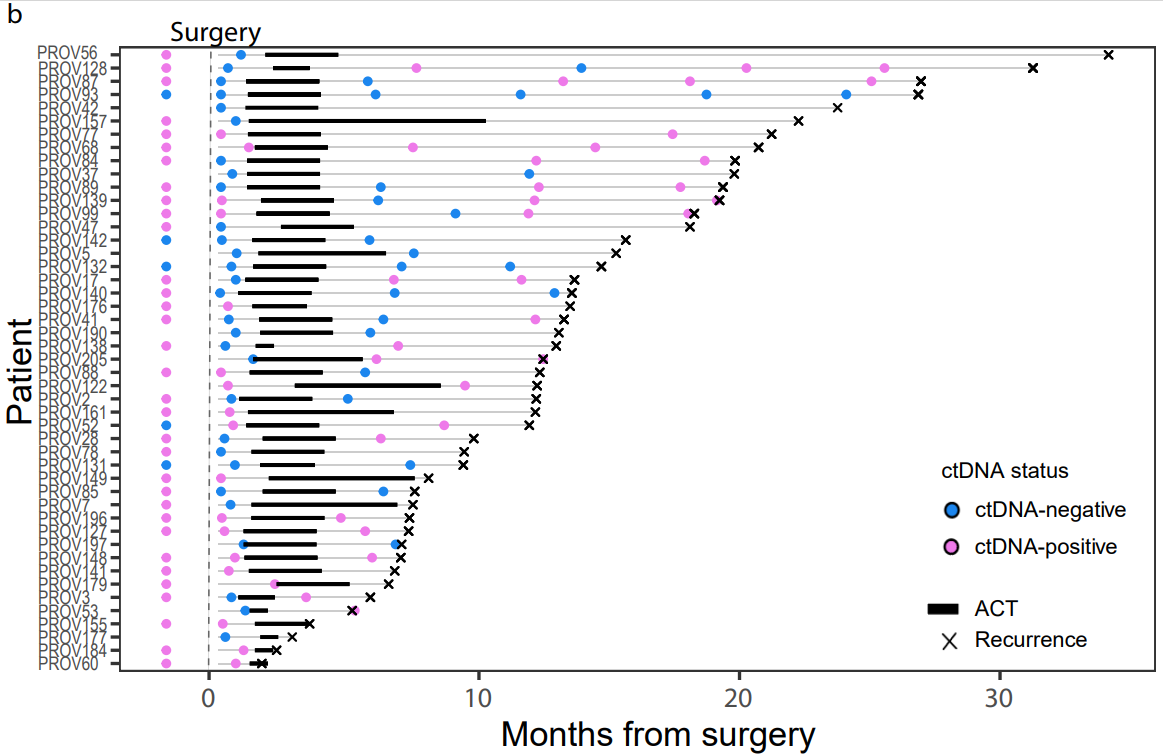


**Supplementary Figure 6.** Swimmer plot of all samples analysed for patients who remained a) recurrence-free or b) experienced a recurrence during the follow up reported.

**Supplementary Table 1**. Summary of baseline clinicopathological characteristics for the PROVENC3 cohort.

|  |  | **Total** | | **ctDNA positive** | | **ctDNA negative** | | **p.value** |
| --- | --- | --- | --- | --- | --- | --- | --- | --- |
|  |  | **n** | **%** | **n** | **%** | **n** | **%** | **0.05 significance level** |
|  |  | 209 | 100 | 28 | 13.4 | 181 | 86.6 |  |
| **Median age (years)** | |  | | 68 (43-83) | | 63 (32-79) | | 0.110 |
| **Sex** |  |  |  |  |  |  |  |  |
|  | **Female** | 97 | 46.4 | 9 | 32.1 | 88 | 48.6 | 0.153 |
|  | **Male** | 112 | 53.6 | 19 | 67.9 | 93 | 51.4 |  |
| **Pathological risk** | |  |  |  |  |  |  |  |
|  | **Low risk** | 127 | 60.8 | 17 | 60.7 | 110 | 60.8 | 1.000 |
|  | **High risk** | 82 | 39.2 | 11 | 39.3 | 71 | 39.2 |  |
| **T status** |  |  |  |  |  |  |  |  |
|  | **T1** | 4 | 1.9 | 0 | 0.0 | 4 | 2.2 |  |
|  | **T2** | 29 | 13.9 | 3 | 10.7 | 26 | 14.4 |  |
|  | **T3** | 125 | 59.8 | 18 | 64.3 | 107 | 59.1 |  |
|  | **T4** | 51 | 24.4 | 7 | 25.0 | 44 | 24.3 |  |
|  | **T1-3** | 158 | 75.6 | 21 | 75.0 | 137 | 75.7 | 1.000 |
|  | **T4** | 51 | 24.4 | 7 | 25.0 | 44 | 24.3 |  |
| **N status** |  |  |  |  |  |  |  |  |
|  | **N1a** | 54 | 25.8 | 6 | 21.4 | 48 | 26.5 |  |
|  | **N1b** | 80 | 38.3 | 11 | 39.3 | 69 | 38.1 |  |
|  | **N1c** | 11 | 5.3 | 2 | 7.1 | 9 | 5.0 |  |
|  | **N1m** | 9 | 4.3 | 0 | 0.0 | 9 | 5.0 |  |
|  | **N2a** | 31 | 14.8 | 5 | 17.9 | 26 | 14.4 |  |
|  | **N2b** | 24 | 11.5 | 4 | 14.3 | 20 | 11.0 |  |
|  | **N1** | 154 | 73.7 | 19 | 67.9 | 135 | 74.6 | 0.491 |
|  | **N2** | 55 | 26.3 | 9 | 32.1 | 46 | 25.4 |  |
| **MSI status** |  |  |  |  |  |  |  |  |
|  | **Stable (MSS)** | 179 | 85.6 | 26 | 92.9 | 153 | 84.5 | 0.384 |
|  | **Instable (MSI)** | 30 | 14.4 | 2 | 7.1 | 28 | 15.5 |  |
| **Resection** |  |  |  |  |  |  |  |  |
|  | **Radical** | 201 | 96.2 | 24 | 85.7 | 177 | 97.8 | 0.043 |
|  | **Non radical** | 3 | 1.4 | 2 | 7.1 | 1 | 0.6 |  |
|  | **UNK** | 5 | 2.4 | 2 | 7.1 | 3 | 1.7 |  |
| **Histology** |  |  |  |  |  |  |  |  |
|  | **Adenocarcinoma, NOS** | 185 | 88.5 | 26 | 92.9 | 159 | 87.8 | 0.636 |
|  | **Medullary carcinoma, NOS** | 3 | 1.4 | 0 | 0.0 | 3 | 1.7 |  |
|  | **Mucinous adenocarcinoma** | 18 | 8.6 | 1 | 3.6 | 17 | 9.4 |  |
|  | **Signet ring cell carcinoma** | 3 | 1.4 | 1 | 3.6 | 2 | 1.1 |  |
| **Differentiation grade** | |  |  |  |  |  |  |  |
|  | **Well differentiated** | 0 | 0.0 | 0 | 0.0 | 0 | 0.0 | 1.000 |
|  | **Moderately differentiated** | 171 | 81.8 | 22 | 78.6 | 149 | 82.3 |  |
|  | **Poorly differentiated** | 29 | 13.9 | 4 | 14.3 | 25 | 13.8 |  |
|  | **Undifferentiated** | 0 | 0.0 | 0 | 0.0 | 0 | 0.0 |  |
|  | **UNK** | 9 | 4.3 | 2 | 7.1 | 7 | 3.9 |  |
| **Tumour location** | |  |  |  |  |  |  |  |
|  | **Left** | 119 | 56.9 | 13 | 46.4 | 106 | 58.6 | 0.305 |
|  | **Right** | 90 | 43.1 | 15 | 53.6 | 75 | 41.4 |  |
| **Angioinvasion** | |  |  |  |  |  |  |  |
|  | **Extramural venous invasion** | 40 | 19.1 | 6 | 21.4 | 34 | 18.8 | 0.664 |
|  | **Intramural venous invasion** | 6 | 2.9 | 1 | 3.6 | 5 | 2.8 |  |
|  | **No** | 108 | 51.7 | 13 | 46.4 | 95 | 52.5 |  |
|  | **UNK** | 55 | 26.3 | 8 | 28.6 | 47 | 26.0 |  |
| **RAS** |  |  |  |  | 0.0 |  |  |  |
|  | **WT** | 126 | 60.3 | 13 | 46.4 | 113 | 62.4 | 0.146 |
|  | **mut** | 83 | 39.7 | 15 | 53.6 | 68 | 37.6 |  |
| **BRAF** |  |  |  |  |  |  |  |  |
|  | **WT** | 167 | 79.9 | 21 | 75.0 | 146 | 80.7 | 0.457 |
|  | **mut** | 42 | 20.1 | 7 | 25.0 | 35 | 19.3 |  |
| **ACT type** |  |  |  |  |  |  |  |  |
|  | **CAPOX 1-4 cycles*** | 188 | 90.0 | 20 | 71.4 | 168 | 92.8 |  |
|  | **CAPOX 5-8 cycles**** | 9 | 4.3 | 2 | 7.1 | 7 | 3.9 |  |
|  | **FOLFOX 8 cycles** | 1 | 1.0 | 0 | 0.0 | 1 | 0.6 |  |
|  | **Capecitabine 1-7 cycles** | 4 | 1.9 | 2 | 7.1 | 2 | 1.1 |  |
|  | **Capecitabine 8 cycles** | 7 | 3.0 | 4 | 14.3 | 3 | 1.7 |  |

* 89% of the patients receiving CAPOX 1-4 cycles received 4 cycles

** 60% of the patients receiving CAPOX 5-8 cycles received 8 cycles

Baseline characteristics and ACT type received stratified by ctDNA status post-surgery. MSI, microsatellite instability; MSS, Microsatellite stability; ctDNA, circulating tumour DNA; UNK, unknown. Fisher exact test, significance level 0.05. "UNK" values excluded from the statistical analysis.

**Supplementary Table 2.** Summary of whole genome sequencing QC metrics for cell line, tumour, and germline analytical study cohort

| **Sample ID** | **Alternate ID** | **Sample Source** | **Sample Type** | **Tumour Type** | **Total Reads** | **Mean Coverage (fold)** | **Tumour and Normal SNP Concordance** | **Median Insert Size (bp)** | **Duplication** | **Final Variant Count (Single Nucleotide Variants)** |
| --- | --- | --- | --- | --- | --- | --- | --- | --- | --- | --- |
| BM72167 | COLO-829 | Normal | Cell Line | - | 874,440,842 | 36.6 | 99.9% | 408 | 13.9% | - |
| BM72162 | COLO-829 | Tumour | Cell Line | Melanoma | 2,359,584,240 | 97.6 | 99.9% | 417 | 15.1% | 15,285 |
| BM72187 | HCC-1143 | Normal | Cell Line | - | 861,687,370 | 36.2 | 99.8% | 412 | 13.0% | - |
| BM72172 | HCC-1143 | Tumour | Cell Line | Breast | 2,365,677,758 | 97.2 | 99.8% | 358 | 13.3% | 8,660 |
| BM72182 | HCC-1187 | Normal | Cell Line | - | 881,856,962 | 36.5 | 99.9% | 417 | 14.1% | - |
| BM72177 | HCC-1187 | Tumour | Cell Line | Breast | 2,436,880,216 | 100.6 | 99.9% | 418 | 14.1% | 5,296 |
| BM69240 | HCC-1954 | Normal | Cell Line | - | 887,445,692 | 37.2 | 99.8% | 420 | 13.1% | - |
| BM69235 | HCC-1954 | Tumour | Cell Line | Breast | 2,415,547,926 | 98.3 | 99.8% | 411 | 15.4% | 9,079 |
| BM72207 | SeraSeq TMB26 | Normal | Cell Line | - | 883,852,012 | 35.9 | 98.9% | 401 | 15.2% | - |
| BM72206 | SeraSeq TMB26 | Tumour | Cell Line | Lung | 2,359,366,754 | 99.5 | 98.9% | 426 | 13.9% | 62,330 |
| BM74017 | SU8790 | Normal | Clinical | - | 874,806,102 | 37.2 | 99.9% | 420 | 13.2% | - |
| BM74276 | SU8790 | Tumour | Clinical | Colorectal | 2,386,887,084 | 71.9 | 99.9% | 256 | 24.0% | 7,138 |
| BM74023 | SU8792 | Normal | Clinical | - | 875,165,910 | 39.5 | 100.0% | 418 | 7.9% | - |
| BM75293 | SU8792 | Tumour | Clinical | Colorectal | 1,829,894,526 | 64.6 | 100.0% | 256 | 13.2% | 2,613 |
| BM74021 | SU8794 | Normal | Clinical | - | 1,438,861,178 | 64.5 | 100.0% | 411 | 8.1% | - |
| BM75294 | SU8794 | Tumour | Clinical | Colorectal | 1,838,113,306 | 68.6 | 100.0% | 275 | 11.4% | 6,299 |
| BM74019 | SU8799 | Normal | Clinical | - | 1,257,011,362 | 56.4 | 100.0% | 406 | 7.8% | - |
| BM75295 | SU8799 | Tumour | Clinical | Colorectal | 1,766,507,188 | 63.5 | 99.8% | 265 | 13.1% | 4,158 |
| BM74025 | SU8804 | Normal | Clinical | - | 889,051,342 | 37.2 | 99.9% | 407 | 13.7% | - |
| BM74277 | SU8804 | Tumour | Clinical | Head and Neck | 2,410,084,568 | 80.8 | 99.9% | 275 | 20.4% | 26,076 |
| BM74027 | SU8809 | Normal | Clinical | - | 910,570,848 | 38.8 | 99.9% | 418 | 13.4% | - |
| BM74278 | SU8809 | Tumour | Clinical | Head and Neck | 2,399,736,340 | 86.0 | 99.9% | 309 | 19.5% | 10,800 |
| BM74029 | SU8810 | Normal | Clinical | - | 895,625,126 | 36.7 | 99.9% | 404 | 14.3% | - |
| BM74279 | SU8810 | Tumour | Clinical | Colorectal | 2,391,847,406 | 86.5 | 99.9% | 322 | 19.2% | 9,979 |
| BM74031 | SU8820 | Normal | Clinical | - | 877,151,534 | 37.3 | 99.9% | 414 | 13.0% | - |
| BM74280 | SU8820 | Tumour | Clinical | Colorectal | 2,424,542,824 | 85.2 | 99.9% | 295 | 20.0% | 5,899 |
| BM74033 | SU8821 | Normal | Clinical | - | 893,703,240 | 37.6 | 100.0% | 422 | 14.4% | - |
| BM74281 | SU8821 | Tumour | Clinical | Head and Neck | 2,402,033,910 | 84.3 | 100.0% | 298 | 19.9% | 10,943 |
| BM74035 | SU8825 | Normal | Clinical | - | 883,453,530 | 37.4 | 99.8% | 414 | 13.3% | - |
| BM74282 | SU8825 | Tumour | Clinical | Head and Neck | 2,394,121,344 | 88.0 | 99.8% | 326 | 18.8% | 7,655 |
| BM74037 | SU8830 | Normal | Clinical | - | 877,705,386 | 36.3 | 99.9% | 406 | 13.7% | - |
| BM74283 | SU8830 | Tumour | Clinical | Colorectal | 2,399,253,438 | 85.6 | 99.9% | 317 | 19.5% | 3,959 |
| BM74041 | SU8836 | Normal | Clinical | - | 888,043,748 | 36.7 | 99.8% | 401 | 13.5% | - |
| BM74284 | SU8836 | Tumour | Clinical | Colorectal | 2,475,871,846 | 86.2 | 99.8% | 287 | 18.4% | 7,621 |
| BM74043 | SU8837 | Normal | Clinical | - | 895,914,540 | 37.1 | 99.7% | 407 | 13.6% | - |
| BM74285 | SU8837 | Tumour | Clinical | Colorectal | 2,444,344,116 | 74.6 | 99.7% | 259 | 23.4% | 25,639 |
| BM75297 | SU8842 | Normal | Clinical | - | 1,196,415,136 | 53.6 | 100.0% | 397 | 7.6% | - |
| BM74286 | SU8842 | Tumour | Clinical | Head and Neck | 1,850,224,326 | 63.5 | 100.0% | 242 | 13.7% | 6,557 |

**Supplementary Table 3.** Summary of whole genome sequencing QC metrics for cell-free and contrived DNA analytical study cohort.

| **Sample ID** | **Sample Source** | **Reference Tumour Content** | **Analytical Study** | **Total Reads** | **Total Reads Aligned** | **Median Insert Size (bp)** | **Mean Coverage (fold)** | **Fraction of Genome with**  **≥10-fold Depth** |
| --- | --- | --- | --- | --- | --- | --- | --- | --- |
| BM70625_R01 | Noncancerous donor plasma | 0.000% | Specificity | 1,404,432,378 | 99.8% | 171 | 41.0 | 95.7% |
| BM70625_R02 | Noncancerous donor plasma | 0.000% | Specificity | 1,101,807,902 | 99.8% | 172 | 32.3 | 94.8% |
| BM70625_R03 | Noncancerous donor plasma | 0.000% | Specificity | 1,492,543,704 | 99.8% | 173 | 45.5 | 95.9% |
| BM70627_R01 | Noncancerous donor plasma | 0.000% | Specificity | 1,099,299,012 | 99.6% | 171 | 31.9 | 93.2% |
| BM70627_R02 | Noncancerous donor plasma | 0.000% | Specificity | 1,383,922,920 | 99.6% | 171 | 39.9 | 94.8% |
| BM70627_R03 | Noncancerous donor plasma | 0.000% | Specificity | 1,271,539,994 | 99.6% | 173 | 37.9 | 94.4% |
| BM70628_R01 | Noncancerous donor plasma | 0.000% | Specificity | 1,356,763,632 | 99.8% | 171 | 39.3 | 95.5% |
| BM70628_R02 | Noncancerous donor plasma | 0.000% | Specificity | 1,091,479,596 | 99.8% | 172 | 31.9 | 94.8% |
| BM70628_R03 | Noncancerous donor plasma | 0.000% | Specificity | 1,330,983,082 | 99.7% | 173 | 40.2 | 95.7% |
| BM70633_R01 | Noncancerous donor plasma | 0.000% | Specificity | 1,467,935,278 | 99.7% | 169 | 40.9 | 95.3% |
| BM70633_R02 | Noncancerous donor plasma | 0.000% | Specificity | 904,151,206 | 99.7% | 170 | 25.5 | 91.6% |
| BM70633_R03 | Noncancerous donor plasma | 0.000% | Specificity | 1,620,363,320 | 99.7% | 170 | 47.2 | 95.8% |
| BM70634_R01 | Noncancerous donor plasma | 0.000% | Specificity | 1,372,641,136 | 99.8% | 170 | 39.6 | 95.2% |
| BM70634_R02 | Noncancerous donor plasma | 0.000% | Specificity | 1,501,344,308 | 99.8% | 170 | 43.6 | 95.6% |
| BM70634_R03 | Noncancerous donor plasma | 0.000% | Specificity | 1,528,865,634 | 99.8% | 171 | 46.4 | 95.7% |
| BM70635_R01 | Noncancerous donor plasma | 0.000% | Specificity | 1,418,225,476 | 99.7% | 170 | 40.2 | 95.6% |
| BM70635_R02 | Noncancerous donor plasma | 0.000% | Specificity | 1,388,212,102 | 99.7% | 170 | 39.7 | 95.6% |
| BM70635_R03 | Noncancerous donor plasma | 0.000% | Specificity | 1,415,819,430 | 99.7% | 172 | 41.9 | 95.8% |
| BM70636_R01 | Noncancerous donor plasma | 0.000% | Specificity | 1,637,648,032 | 99.7% | 169 | 46.8 | 95.5% |
| BM70636_R02 | Noncancerous donor plasma | 0.000% | Specificity | 1,541,263,676 | 99.7% | 169 | 44.0 | 95.5% |
| BM70636_R03 | Noncancerous donor plasma | 0.000% | Specificity | 1,347,559,756 | 99.7% | 170 | 39.8 | 95.3% |
| BM70637_R01 | Noncancerous donor plasma | 0.000% | Specificity | 1,080,550,962 | 99.8% | 165 | 29.5 | 92.0% |
| BM70637_R02 | Noncancerous donor plasma | 0.000% | Specificity | 1,515,016,818 | 99.8% | 166 | 41.4 | 94.4% |
| BM70637_R03 | Noncancerous donor plasma | 0.000% | Specificity | 1,540,511,436 | 99.7% | 166 | 43.4 | 94.6% |
| BM70638_R01 | Noncancerous donor plasma | 0.000% | Specificity | 1,302,832,556 | 99.7% | 172 | 37.9 | 80.0% |
| BM70638_R02 | Noncancerous donor plasma | 0.000% | Specificity | 1,192,439,890 | 99.7% | 172 | 34.6 | 78.8% |
| BM70638_R03 | Noncancerous donor plasma | 0.000% | Specificity | 1,337,483,906 | 99.7% | 174 | 41.4 | 81.6% |
| BM70641_R01 | Noncancerous donor plasma | 0.000% | Specificity | 1,272,839,438 | 99.6% | 170 | 36.0 | 95.2% |
| BM70641_R02 | Noncancerous donor plasma | 0.000% | Specificity | 1,644,600,182 | 99.6% | 170 | 46.6 | 95.9% |
| BM70641_R03 | Noncancerous donor plasma | 0.000% | Specificity | 1,334,526,680 | 99.6% | 171 | 38.8 | 95.5% |
| BM70642_R01 | Noncancerous donor plasma | 0.000% | Specificity | 1,143,084,716 | 99.6% | 171 | 33.0 | 94.5% |
| BM70642_R02 | Noncancerous donor plasma | 0.000% | Specificity | 1,344,906,668 | 99.6% | 171 | 38.6 | 95.1% |
| BM70642_R03 | Noncancerous donor plasma | 0.000% | Specificity | 1,314,808,500 | 99.6% | 172 | 38.7 | 95.2% |
| BM70643_R01 | Noncancerous donor plasma | 0.000% | Specificity | 1,156,230,280 | 99.6% | 171 | 33.0 | 94.1% |
| BM70643_R02 | Noncancerous donor plasma | 0.000% | Specificity | 1,304,536,032 | 99.6% | 171 | 37.3 | 94.9% |
| BM70643_R03 | Noncancerous donor plasma | 0.000% | Specificity | 1,461,718,268 | 99.6% | 172 | 43.1 | 95.5% |
| BM70644_R01 | Noncancerous donor plasma | 0.000% | Specificity | 1,167,941,964 | 99.6% | 172 | 33.4 | 92.3% |
| BM70644_R02 | Noncancerous donor plasma | 0.000% | Specificity | 1,074,557,678 | 99.6% | 172 | 30.9 | 91.4% |
| BM70644_R03 | Noncancerous donor plasma | 0.000% | Specificity | 1,017,623,420 | 99.6% | 173 | 29.7 | 90.6% |
| BM70645_R01 | Noncancerous donor plasma | 0.000% | Specificity | 1,229,416,956 | 99.6% | 175 | 36.9 | 91.8% |
| BM70645_R02 | Noncancerous donor plasma | 0.000% | Specificity | 1,273,854,534 | 99.7% | 176 | 38.6 | 92.4% |
| BM70645_R03 | Noncancerous donor plasma | 0.000% | Specificity | 962,385,936 | 99.6% | 177 | 29.9 | 89.5% |
| BM70647_R01 | Noncancerous donor plasma | 0.000% | Specificity | 1,787,438,138 | 99.8% | 168 | 50.1 | 95.7% |
| BM70647_R02 | Noncancerous donor plasma | 0.000% | Specificity | 1,513,079,160 | 99.8% | 168 | 42.8 | 95.5% |
| BM70647_R03 | Noncancerous donor plasma | 0.000% | Specificity | 1,449,195,976 | 99.7% | 168 | 41.4 | 95.5% |
| BM70648_R01 | Noncancerous donor plasma | 0.000% | Specificity | 1,580,869,782 | 99.7% | 167 | 44.6 | 95.5% |
| BM70648_R02 | Noncancerous donor plasma | 0.000% | Specificity | 1,141,038,564 | 99.7% | 167 | 32.2 | 94.6% |
| BM70648_R03 | Noncancerous donor plasma | 0.000% | Specificity | 1,284,826,268 | 99.7% | 168 | 37.2 | 95.1% |
| BM70649_R01 | Noncancerous donor plasma | 0.000% | Specificity | 2,035,773,052 | 99.6% | 169 | 57.9 | 95.8% |
| BM70649_R02 | Noncancerous donor plasma | 0.000% | Specificity | 1,199,617,574 | 99.6% | 169 | 34.1 | 95.0% |
| BM70649_R03 | Noncancerous donor plasma | 0.000% | Specificity | 1,135,440,718 | 99.6% | 170 | 33.3 | 94.9% |
| BM70650_R01 | Noncancerous donor plasma | 0.000% | Specificity | 1,359,667,494 | 99.6% | 170 | 38.7 | 95.0% |
| BM70650_R02 | Noncancerous donor plasma | 0.000% | Specificity | 1,325,073,968 | 99.6% | 170 | 37.9 | 95.0% |
| BM70650_R03 | Noncancerous donor plasma | 0.000% | Specificity | 1,287,643,800 | 99.6% | 171 | 37.5 | 94.9% |
| BM70651_R01 | Noncancerous donor plasma | 0.000% | Specificity | 1,757,644,476 | 99.5% | 168 | 49.4 | 95.6% |
| BM70651_R02 | Noncancerous donor plasma | 0.000% | Specificity | 1,263,296,240 | 99.6% | 168 | 35.9 | 94.9% |
| BM70651_R03 | Noncancerous donor plasma | 0.000% | Specificity | 1,302,328,554 | 99.5% | 170 | 37.5 | 95.0% |
| BM71950_R01 | Noncancerous donor plasma | 0.000% | Specificity | 1,565,643,922 | 99.8% | 170 | 45.4 | 95.6% |
| BM71950_R02 | Noncancerous donor plasma | 0.000% | Specificity | 1,417,568,796 | 99.8% | 170 | 41.2 | 95.5% |
| BM71950_R03 | Noncancerous donor plasma | 0.000% | Specificity | 1,276,105,046 | 99.8% | 171 | 37.8 | 95.4% |
| BM71951_R01 | Noncancerous donor plasma | 0.000% | Specificity | 1,310,562,416 | 99.8% | 171 | 38.6 | 95.5% |
| BM71951_R02 | Noncancerous donor plasma | 0.000% | Specificity | 1,387,961,596 | 99.8% | 172 | 40.8 | 95.6% |
| BM71951_R03 | Noncancerous donor plasma | 0.000% | Specificity | 1,027,557,334 | 99.8% | 173 | 30.8 | 95.0% |
| BM71952_R01 | Noncancerous donor plasma | 0.000% | Specificity | 936,503,204 | 99.8% | 169 | 27.1 | 94.4% |
| BM71952_R02 | Noncancerous donor plasma | 0.000% | Specificity | 1,355,300,064 | 99.8% | 170 | 39.4 | 95.4% |
| BM71952_R03 | Noncancerous donor plasma | 0.000% | Specificity | 1,373,798,064 | 99.8% | 171 | 40.6 | 95.5% |
| BM71953_R01 | Noncancerous donor plasma | 0.000% | Specificity | 1,452,015,228 | 99.8% | 170 | 41.7 | 95.7% |
| BM71953_R02 | Noncancerous donor plasma | 0.000% | Specificity | 1,292,054,980 | 99.8% | 170 | 37.3 | 95.4% |
| BM71953_R03 | Noncancerous donor plasma | 0.000% | Specificity | 1,389,741,148 | 99.7% | 171 | 40.7 | 95.7% |
| BM71959_R01 | Noncancerous donor plasma | 0.000% | Specificity | 1,481,547,222 | 99.8% | 169 | 42.6 | 95.8% |
| BM71959_R02 | Noncancerous donor plasma | 0.000% | Specificity | 1,377,024,286 | 99.8% | 169 | 39.5 | 95.6% |
| BM71959_R03 | Noncancerous donor plasma | 0.000% | Specificity | 1,202,701,644 | 99.8% | 170 | 35.1 | 95.2% |
| BM71960_R01 | Noncancerous donor plasma | 0.000% | Specificity | 1,591,212,200 | 99.8% | 166 | 44.1 | 95.6% |
| BM71960_R02 | Noncancerous donor plasma | 0.000% | Specificity | 1,396,344,262 | 99.8% | 166 | 38.8 | 95.4% |
| BM71960_R03 | Noncancerous donor plasma | 0.000% | Specificity | 1,316,003,770 | 99.8% | 167 | 37.1 | 95.4% |
| BM71961_R01 | Noncancerous donor plasma | 0.000% | Specificity | 1,231,891,878 | 99.8% | 170 | 35.6 | 95.0% |
| BM71961_R02 | Noncancerous donor plasma | 0.000% | Specificity | 1,334,040,280 | 99.8% | 171 | 39.0 | 95.2% |
| BM71961_R03 | Noncancerous donor plasma | 0.000% | Specificity | 1,274,354,940 | 99.8% | 171 | 37.6 | 95.1% |
| BM71962_R01 | Noncancerous donor plasma | 0.000% | Specificity | 1,334,473,404 | 99.8% | 169 | 38.3 | 95.2% |
| BM71962_R02 | Noncancerous donor plasma | 0.000% | Specificity | 1,568,749,966 | 99.8% | 169 | 45.2 | 95.5% |
| BM71962_R03 | Noncancerous donor plasma | 0.000% | Specificity | 1,317,497,952 | 99.8% | 170 | 38.2 | 95.2% |
| BM71964_R01 | Noncancerous donor plasma | 0.000% | Specificity | 724,528,982 | 99.7% | 170 | 20.7 | 77.3% |
| BM71964_R02 | Noncancerous donor plasma | 0.000% | Specificity | 1,327,708,312 | 99.7% | 170 | 38.2 | 86.0% |
| BM71964_R03 | Noncancerous donor plasma | 0.000% | Specificity | 1,347,825,346 | 99.7% | 171 | 39.4 | 86.4% |
| BM71982_R01 | Noncancerous donor plasma | 0.000% | Specificity | 1,143,182,046 | 99.7% | 170 | 33.1 | 93.8% |
| BM71982_R02 | Noncancerous donor plasma | 0.000% | Specificity | 1,290,116,580 | 99.7% | 171 | 37.7 | 94.7% |
| BM71982_R03 | Noncancerous donor plasma | 0.000% | Specificity | 1,410,520,762 | 99.7% | 171 | 41.8 | 95.1% |
| BM71983_R01 | Noncancerous donor plasma | 0.000% | Specificity | 1,038,646,060 | 99.7% | 173 | 31.1 | 94.3% |
| BM71983_R02 | Noncancerous donor plasma | 0.000% | Specificity | 1,262,708,816 | 99.7% | 173 | 38.2 | 95.1% |
| BM71983_R03 | Noncancerous donor plasma | 0.000% | Specificity | 1,453,729,108 | 99.7% | 175 | 44.8 | 95.5% |
| BM71985_R01 | Noncancerous donor plasma | 0.000% | Specificity | 1,053,586,760 | 99.7% | 175 | 32.0 | 94.8% |
| BM71985_R02 | Noncancerous donor plasma | 0.000% | Specificity | 1,520,556,596 | 99.6% | 175 | 45.3 | 95.6% |
| BM71985_R03 | Noncancerous donor plasma | 0.000% | Specificity | 1,021,066,230 | 99.7% | 174 | 30.1 | 94.4% |
| BM71986_R01 | Noncancerous donor plasma | 0.000% | Specificity | 1,323,087,684 | 99.7% | 171 | 38.2 | 83.8% |
| BM71986_R03 | Noncancerous donor plasma | 0.000% | Specificity | 1,148,618,476 | 99.7% | 170 | 32.5 | 81.8% |
| BM71987_R01 | Noncancerous donor plasma | 0.000% | Specificity | 1,337,553,524 | 99.8% | 167 | 37.2 | 95.6% |
| BM71987_R02 | Noncancerous donor plasma | 0.000% | Specificity | 1,307,249,972 | 99.8% | 167 | 36.3 | 95.5% |
| BM71987_R03 | Noncancerous donor plasma | 0.000% | Specificity | 1,066,445,426 | 99.8% | 167 | 29.4 | 94.5% |
| BM71988_R01 | Noncancerous donor plasma | 0.000% | Specificity | 1,768,344,152 | 99.6% | 170 | 50.5 | 95.8% |
| BM71988_R02 | Noncancerous donor plasma | 0.000% | Specificity | 1,595,968,518 | 99.6% | 170 | 45.9 | 95.7% |
| BM71988_R03 | Noncancerous donor plasma | 0.000% | Specificity | 898,384,396 | 99.6% | 169 | 25.1 | 94.0% |
| BM71989_R01 | Noncancerous donor plasma | 0.000% | Specificity | 1,179,956,004 | 99.8% | 172 | 34.4 | 95.3% |
| BM71989_R02 | Noncancerous donor plasma | 0.000% | Specificity | 1,341,132,372 | 99.8% | 172 | 39.1 | 95.5% |
| BM71989_R03 | Noncancerous donor plasma | 0.000% | Specificity | 1,150,249,746 | 99.8% | 171 | 32.7 | 95.1% |
| BM71990_R01 | Noncancerous donor plasma | 0.000% | Specificity | 1,290,171,974 | 99.7% | 167 | 36.0 | 95.3% |
| BM71990_R02 | Noncancerous donor plasma | 0.000% | Specificity | 1,069,467,668 | 99.8% | 167 | 29.3 | 94.8% |
| BM71990_R03 | Noncancerous donor plasma | 0.000% | Specificity | 1,082,552,742 | 99.8% | 167 | 29.6 | 94.8% |
| BM71991_R01 | Noncancerous donor plasma | 0.000% | Specificity | 1,096,537,210 | 99.8% | 170 | 31.5 | 95.1% |
| BM71991_R02 | Noncancerous donor plasma | 0.000% | Specificity | 1,537,128,610 | 99.8% | 168 | 42.9 | 95.5% |
| BM71991_R03 | Noncancerous donor plasma | 0.000% | Specificity | 1,207,078,844 | 99.8% | 168 | 33.7 | 95.1% |
| BM72157_R01 | Noncancerous donor plasma | 0.000% | Specificity | 1,226,148,398 | 99.7% | 169 | 34.7 | 94.5% |
| BM72157_R02 | Noncancerous donor plasma | 0.000% | Specificity | 1,021,849,100 | 99.8% | 169 | 28.9 | 93.5% |
| BM72157_R03 | Noncancerous donor plasma | 0.000% | Specificity | 1,075,878,540 | 99.7% | 169 | 29.8 | 93.7% |
| BM72158_R01 | Noncancerous donor plasma | 0.000% | Specificity | 1,150,077,144 | 99.8% | 171 | 33.2 | 94.6% |
| BM72158_R02 | Noncancerous donor plasma | 0.000% | Specificity | 1,130,803,266 | 99.8% | 170 | 31.9 | 94.4% |
| BM72158_R03 | Noncancerous donor plasma | 0.000% | Specificity | 1,450,575,992 | 99.8% | 170 | 41.0 | 95.3% |
| BM72159_R01 | Noncancerous donor plasma | 0.000% | Specificity | 1,478,685,582 | 99.8% | 167 | 40.9 | 95.6% |
| BM72159_R02 | Noncancerous donor plasma | 0.000% | Specificity | 1,320,700,234 | 99.8% | 167 | 35.7 | 95.1% |
| BM72159_R03 | Noncancerous donor plasma | 0.000% | Specificity | 1,601,225,142 | 99.8% | 167 | 43.3 | 95.7% |
| CATES_BM72162_Dilution1_R01 | COLO-829 | 10.000% | Sensitivity | 873,265,318 | 99.9% | 154 | 22.2 | 93.2% |
| CATES_BM72162_Dilution1_R02 | COLO-829 | 10.000% | Sensitivity | 1,111,353,004 | 99.9% | 148 | 27.0 | 94.7% |
| CATES_BM72162_Dilution1_R03 | COLO-829 | 10.000% | Sensitivity | 1,171,199,196 | 99.9% | 152 | 29.3 | 95.1% |
| CATES_BM72162_Dilution2_R01 | COLO-829 | 1.000% | Sensitivity | 1,008,915,734 | 99.9% | 154 | 25.6 | 94.5% |
| CATES_BM72162_Dilution2_R02 | COLO-829 | 1.000% | Sensitivity | 1,308,395,910 | 99.9% | 148 | 31.6 | 95.5% |
| CATES_BM72162_Dilution2_R03 | COLO-829 | 1.000% | Sensitivity | 1,417,868,546 | 99.9% | 151 | 35.1 | 95.8% |
| CATES_BM72162_Dilution3_R01 | COLO-829 | 0.100% | Sensitivity | 1,102,687,744 | 99.9% | 155 | 28.1 | 95.0% |
| CATES_BM72162_Dilution3_R02 | COLO-829 | 0.100% | Sensitivity | 1,235,382,288 | 99.9% | 149 | 30.0 | 95.2% |
| CATES_BM72162_Dilution3_R03 | COLO-829 | 0.100% | Sensitivity | 1,283,787,460 | 99.9% | 151 | 31.9 | 95.5% |
| CATES_BM72162_Dilution4_R01 | COLO-829 | 0.050% | Sensitivity | 1,335,612,948 | 99.9% | 155 | 34.0 | 95.8% |
| CATES_BM72162_Dilution4_R02 | COLO-829 | 0.050% | Sensitivity | 1,294,642,752 | 99.9% | 149 | 31.6 | 95.5% |
| CATES_BM72162_Dilution4_R03 | COLO-829 | 0.050% | Sensitivity | 1,649,519,040 | 99.9% | 151 | 40.9 | 96.1% |
| CATES_BM72162_Dilution5_R01 | COLO-829 | 0.020% | Sensitivity | 1,434,871,220 | 99.9% | 154 | 36.6 | 95.9% |
| CATES_BM72162_Dilution5_R02 | COLO-829 | 0.020% | Sensitivity | 1,241,749,310 | 99.9% | 155 | 31.5 | 95.5% |
| CATES_BM72162_Dilution5_R03 | COLO-829 | 0.020% | Sensitivity | 1,376,874,352 | 99.9% | 151 | 34.3 | 95.7% |
| CATES_BM72162_Dilution6_R01 | COLO-829 | 0.010% | Sensitivity | 1,125,770,328 | 99.9% | 156 | 28.8 | 95.2% |
| CATES_BM72162_Dilution6_R02 | COLO-829 | 0.010% | Sensitivity | 1,206,706,008 | 99.9% | 147 | 29.1 | 95.1% |
| CATES_BM72162_Dilution6_R03 | COLO-829 | 0.010% | Sensitivity | 1,237,780,928 | 99.9% | 150 | 30.4 | 95.3% |
| CATES_BM72162_Dilution7_R01 | COLO-829 | 0.005% | Sensitivity | 1,386,658,722 | 99.9% | 155 | 35.3 | 95.9% |
| CATES_BM72162_Dilution7_R02 | COLO-829 | 0.005% | Sensitivity | 1,188,190,500 | 99.9% | 148 | 28.7 | 95.0% |
| CATES_BM72162_Dilution7_R03 | COLO-829 | 0.005% | Sensitivity | 1,343,489,786 | 99.9% | 149 | 33.0 | 95.6% |
| CATES_BM72162_Dilution8_R01 | COLO-829 | 0.001% | Sensitivity | 1,271,157,420 | 99.9% | 155 | 32.4 | 95.6% |
| CATES_BM72162_Dilution8_R02 | COLO-829 | 0.001% | Sensitivity | 1,192,054,552 | 99.9% | 147 | 28.7 | 95.0% |
| CATES_BM72162_Dilution8_R03 | COLO-829 | 0.001% | Sensitivity | 1,346,467,316 | 99.9% | 150 | 33.2 | 95.6% |
| CATES_BM72173_Dilution1_R01 | HCC-1143 | 10.000% | Sensitivity | 1,590,122,204 | 99.9% | 155 | 40.8 | 95.7% |
| CATES_BM72173_Dilution1_R02 | HCC-1143 | 10.000% | Sensitivity | 982,543,638 | 99.9% | 147 | 23.9 | 94.4% |
| CATES_BM72173_Dilution1_R03 | HCC-1143 | 10.000% | Sensitivity | 1,419,514,980 | 99.9% | 150 | 35.2 | 95.5% |
| CATES_BM72173_Dilution2_R01 | HCC-1143 | 1.000% | Sensitivity | 1,446,232,424 | 99.9% | 154 | 36.8 | 95.6% |
| CATES_BM72173_Dilution2_R02 | HCC-1143 | 1.000% | Sensitivity | 1,040,827,920 | 99.9% | 146 | 25.1 | 94.7% |
| CATES_BM72173_Dilution2_R03 | HCC-1143 | 1.000% | Sensitivity | 1,456,541,314 | 99.9% | 149 | 35.9 | 95.5% |
| CATES_BM72173_Dilution3_R01 | HCC-1143 | 0.100% | Sensitivity | 1,349,550,776 | 99.9% | 154 | 34.4 | 95.5% |
| CATES_BM72173_Dilution3_R02 | HCC-1143 | 0.100% | Sensitivity | 1,034,553,748 | 99.9% | 147 | 25.1 | 94.7% |
| CATES_BM72173_Dilution3_R03 | HCC-1143 | 0.100% | Sensitivity | 1,304,403,006 | 99.9% | 149 | 32.1 | 95.4% |
| CATES_BM72173_Dilution4_R01 | HCC-1143 | 0.050% | Sensitivity | 1,645,319,872 | 99.9% | 155 | 42.2 | 95.8% |
| CATES_BM72173_Dilution4_R02 | HCC-1143 | 0.050% | Sensitivity | 1,106,090,284 | 99.9% | 147 | 26.7 | 94.9% |
| CATES_BM72173_Dilution4_R03 | HCC-1143 | 0.050% | Sensitivity | 1,865,756,162 | 99.9% | 151 | 46.5 | 95.8% |
| CATES_BM72173_Dilution5_R01 | HCC-1143 | 0.020% | Sensitivity | 1,785,340,436 | 99.9% | 153 | 45.3 | 95.8% |
| CATES_BM72173_Dilution5_R02 | HCC-1143 | 0.020% | Sensitivity | 1,150,619,108 | 99.9% | 158 | 29.8 | 95.3% |
| CATES_BM72173_Dilution5_R03 | HCC-1143 | 0.020% | Sensitivity | 1,353,890,038 | 99.9% | 150 | 33.6 | 95.4% |
| CATES_BM72173_Dilution6_R01 | HCC-1143 | 0.010% | Sensitivity | 1,439,089,348 | 99.9% | 154 | 36.8 | 95.6% |
| CATES_BM72173_Dilution6_R02 | HCC-1143 | 0.010% | Sensitivity | 1,280,446,540 | 99.9% | 147 | 31.0 | 95.3% |
| CATES_BM72173_Dilution6_R03 | HCC-1143 | 0.010% | Sensitivity | 1,246,647,900 | 99.9% | 149 | 30.8 | 95.3% |
| CATES_BM72173_Dilution7_R01 | HCC-1143 | 0.005% | Sensitivity | 1,345,276,590 | 99.9% | 153 | 34.0 | 95.5% |
| CATES_BM72173_Dilution7_R02 | HCC-1143 | 0.005% | Sensitivity | 1,115,933,218 | 99.9% | 146 | 26.8 | 94.8% |
| CATES_BM72173_Dilution7_R03 | HCC-1143 | 0.005% | Sensitivity | 1,218,402,698 | 99.9% | 147 | 29.7 | 95.1% |
| CATES_BM72173_Dilution8_R01 | HCC-1143 | 0.001% | Sensitivity | 1,599,505,650 | 99.9% | 153 | 40.5 | 95.7% |
| CATES_BM72173_Dilution8_R02 | HCC-1143 | 0.001% | Sensitivity | 1,156,548,252 | 99.9% | 146 | 27.8 | 95.0% |
| CATES_BM72173_Dilution8_R03 | HCC-1143 | 0.001% | Sensitivity | 1,378,058,644 | 99.9% | 151 | 34.3 | 95.5% |
| CAT_BM72177_Dilution1_R01 | HCC-1187 | 0.050% | Sensitivity | 1,078,043,626 | 99.9% | 161 | 28.6 | 95.3% |
| CAT_BM72177_Dilution1_R02 | HCC-1187 | 0.050% | Sensitivity | 1,088,895,970 | 99.9% | 162 | 29.0 | 95.3% |
| CAT_BM72177_Dilution1_R03 | HCC-1187 | 0.050% | Sensitivity | 1,167,788,914 | 100.0% | 161 | 31.0 | 95.5% |
| CAT_BM72177_Dilution2_R01 | HCC-1187 | 0.010% | Sensitivity | 1,161,268,448 | 100.0% | 161 | 30.7 | 95.4% |
| CAT_BM72177_Dilution2_R03 | HCC-1187 | 0.010% | Sensitivity | 1,224,921,074 | 99.9% | 167 | 33.6 | 95.6% |
| CAT_BM72177_Dilution3_R01 | HCC-1187 | 0.005% | Sensitivity | 1,044,558,184 | 100.0% | 161 | 27.6 | 95.2% |
| CAT_BM72177_Dilution3_R02 | HCC-1187 | 0.005% | Sensitivity | 1,164,836,974 | 100.0% | 160 | 30.7 | 95.4% |
| CAT_BM72177_Dilution3_R03 | HCC-1187 | 0.005% | Sensitivity | 1,136,163,550 | 99.9% | 161 | 30.1 | 95.4% |
| CAT_BM72177_Dilution4_R01 | HCC-1187 | 0.001% | Sensitivity | 1,192,770,774 | 99.9% | 162 | 31.8 | 95.5% |
| CAT_BM72177_Dilution4_R02 | HCC-1187 | 0.001% | Sensitivity | 1,189,164,620 | 100.0% | 161 | 31.4 | 95.5% |
| CAT_BM72177_Dilution4_R03 | HCC-1187 | 0.001% | Sensitivity | 1,170,458,910 | 99.9% | 161 | 31.1 | 95.5% |
| CATES_BM72178_Dilution1_R01 | HCC-1187 | 10.000% | Sensitivity | 1,548,910,636 | 100.0% | 154 | 39.6 | 95.7% |
| CATES_BM72178_Dilution1_R02 | HCC-1187 | 10.000% | Sensitivity | 1,251,404,706 | 100.0% | 154 | 32.2 | 95.5% |
| CATES_BM72178_Dilution1_R03 | HCC-1187 | 10.000% | Sensitivity | 1,190,398,470 | 100.0% | 153 | 30.2 | 95.3% |
| CATES_BM72178_Dilution2_R01 | HCC-1187 | 1.000% | Sensitivity | 1,476,097,496 | 100.0% | 154 | 37.7 | 95.7% |
| CATES_BM72178_Dilution2_R02 | HCC-1187 | 1.000% | Sensitivity | 1,384,234,084 | 100.0% | 154 | 35.5 | 95.6% |
| CATES_BM72178_Dilution2_R03 | HCC-1187 | 1.000% | Sensitivity | 1,280,043,288 | 100.0% | 152 | 32.3 | 95.4% |
| CATES_BM72178_Dilution3_R01 | HCC-1187 | 0.100% | Sensitivity | 1,427,383,710 | 100.0% | 154 | 36.5 | 95.6% |
| CATES_BM72178_Dilution3_R02 | HCC-1187 | 0.100% | Sensitivity | 1,299,304,092 | 100.0% | 154 | 33.3 | 95.5% |
| CATES_BM72178_Dilution3_R03 | HCC-1187 | 0.100% | Sensitivity | 1,313,125,590 | 100.0% | 152 | 33.2 | 95.5% |
| CATES_BM72178_Dilution4_R01 | HCC-1187 | 0.050% | Sensitivity | 1,408,689,732 | 100.0% | 153 | 35.8 | 95.6% |
| CATES_BM72178_Dilution4_R02 | HCC-1187 | 0.050% | Sensitivity | 1,472,572,052 | 100.0% | 155 | 37.9 | 95.7% |
| CATES_BM72178_Dilution4_R03 | HCC-1187 | 0.050% | Sensitivity | 1,405,895,040 | 100.0% | 153 | 35.7 | 95.6% |
| CATES_BM72178_Dilution5_R01 | HCC-1187 | 0.020% | Sensitivity | 1,585,433,646 | 100.0% | 154 | 40.6 | 95.7% |
| CATES_BM72178_Dilution5_R02 | HCC-1187 | 0.020% | Sensitivity | 1,337,230,120 | 100.0% | 155 | 34.6 | 95.6% |
| CATES_BM72178_Dilution5_R03 | HCC-1187 | 0.020% | Sensitivity | 1,361,557,768 | 100.0% | 154 | 34.9 | 95.5% |
| CATES_BM72178_Dilution6_R01 | HCC-1187 | 0.010% | Sensitivity | 1,341,482,816 | 100.0% | 153 | 34.1 | 95.5% |
| CATES_BM72178_Dilution6_R02 | HCC-1187 | 0.010% | Sensitivity | 1,216,252,372 | 100.0% | 154 | 31.3 | 95.4% |
| CATES_BM72178_Dilution6_R03 | HCC-1187 | 0.010% | Sensitivity | 1,224,765,884 | 100.0% | 154 | 31.2 | 95.4% |
| CATES_BM72178_Dilution7_R01 | HCC-1187 | 0.005% | Sensitivity | 1,209,212,946 | 100.0% | 154 | 30.8 | 95.4% |
| CATES_BM72178_Dilution7_R02 | HCC-1187 | 0.005% | Sensitivity | 1,257,560,924 | 100.0% | 154 | 32.2 | 95.5% |
| CATES_BM72178_Dilution7_R03 | HCC-1187 | 0.005% | Sensitivity | 1,181,710,674 | 100.0% | 153 | 30.1 | 95.3% |
| CATES_BM72178_Dilution8_R01 | HCC-1187 | 0.001% | Sensitivity | 1,283,856,546 | 100.0% | 154 | 32.7 | 95.4% |
| CATES_BM72178_Dilution8_R02 | HCC-1187 | 0.001% | Sensitivity | 1,287,417,248 | 100.0% | 154 | 33.0 | 95.4% |
| CATES_BM72178_Dilution8_R03 | HCC-1187 | 0.001% | Sensitivity | 1,222,834,882 | 100.0% | 153 | 31.1 | 95.3% |
| CAT_BM56759_Dilution1_R01 | HCC-1954 | 0.050% | Sensitivity | 1,196,199,536 | 99.9% | 162 | 31.9 | 95.5% |
| CAT_BM56759_Dilution1_R02 | HCC-1954 | 0.050% | Sensitivity | 1,268,810,452 | 99.9% | 163 | 33.9 | 95.6% |
| CAT_BM56759_Dilution1_R03 | HCC-1954 | 0.050% | Sensitivity | 1,317,844,824 | 99.9% | 162 | 35.1 | 95.6% |
| CAT_BM56759_Dilution2_R01 | HCC-1954 | 0.010% | Sensitivity | 1,377,691,742 | 99.9% | 162 | 36.7 | 95.7% |
| CAT_BM56759_Dilution2_R02 | HCC-1954 | 0.010% | Sensitivity | 1,354,637,100 | 99.9% | 163 | 36.2 | 95.7% |
| CAT_BM56759_Dilution3_R01 | HCC-1954 | 0.005% | Sensitivity | 1,425,208,750 | 99.9% | 162 | 38.0 | 95.7% |
| CAT_BM56759_Dilution3_R02 | HCC-1954 | 0.005% | Sensitivity | 1,332,088,478 | 99.9% | 163 | 35.6 | 95.7% |
| CAT_BM56759_Dilution3_R03 | HCC-1954 | 0.005% | Sensitivity | 1,259,885,690 | 99.9% | 162 | 33.5 | 95.6% |
| CAT_BM56759_Dilution4_R01 | HCC-1954 | 0.001% | Sensitivity | 1,359,123,490 | 99.9% | 162 | 36.3 | 95.7% |
| CAT_BM56759_Dilution4_R02 | HCC-1954 | 0.001% | Sensitivity | 1,254,345,210 | 99.9% | 162 | 33.4 | 95.6% |
| CAT_BM56759_Dilution4_R03 | HCC-1954 | 0.001% | Sensitivity | 1,246,904,838 | 99.9% | 162 | 33.2 | 95.6% |
| CAT_BM72206_Dilution1_R01 | SeraSeq TMB26 | 0.050% | Sensitivity | 1,714,888,958 | 100.0% | 149 | 41.8 | 95.6% |
| CAT_BM72206_Dilution1_R02 | SeraSeq TMB26 | 0.050% | Sensitivity | 1,004,900,354 | 100.0% | 148 | 24.3 | 94.3% |
| CAT_BM72206_Dilution1_R03 | SeraSeq TMB26 | 0.050% | Sensitivity | 1,142,715,682 | 100.0% | 148 | 27.7 | 94.9% |
| CAT_BM72206_Dilution2_R01 | SeraSeq TMB26 | 0.010% | Sensitivity | 1,310,839,138 | 100.0% | 149 | 32.0 | 95.3% |
| CAT_BM72206_Dilution2_R02 | SeraSeq TMB26 | 0.010% | Sensitivity | 1,259,874,020 | 100.0% | 149 | 30.8 | 95.2% |
| CAT_BM72206_Dilution3_R01 | SeraSeq TMB26 | 0.005% | Sensitivity | 1,317,448,754 | 100.0% | 149 | 32.2 | 95.3% |
| CAT_BM72206_Dilution3_R02 | SeraSeq TMB26 | 0.005% | Sensitivity | 1,242,419,216 | 100.0% | 149 | 30.3 | 95.2% |
| CAT_BM72206_Dilution3_R03 | SeraSeq TMB26 | 0.005% | Sensitivity | 1,216,645,750 | 100.0% | 148 | 29.4 | 95.1% |
| CAT_BM72206_Dilution4_R01 | SeraSeq TMB26 | 0.001% | Sensitivity | 1,327,894,304 | 100.0% | 151 | 32.9 | 95.3% |
| CAT_BM72206_Dilution4_R02 | SeraSeq TMB26 | 0.001% | Sensitivity | 1,176,702,314 | 100.0% | 148 | 28.6 | 95.0% |
| CAT_BM72206_Dilution4_R03 | SeraSeq TMB26 | 0.001% | Sensitivity | 1,213,265,644 | 100.0% | 149 | 29.5 | 95.1% |
| CAT_EC_BM72206_R01 | SeraSeq TMB26 | 0.050% | Sensitivity | 1,738,701,636 | 100.0% | 152 | 44.0 | 95.7% |
| CAT_EC_BM72206_R02 | SeraSeq TMB26 | 0.050% | Sensitivity | 1,161,009,624 | 100.0% | 149 | 29.0 | 94.9% |
| CAT_EC_BM72206_R03 | SeraSeq TMB26 | 0.050% | Sensitivity | 1,599,944,406 | 100.0% | 152 | 40.7 | 95.7% |
| CAT_EC_BM72206_R04 | SeraSeq TMB26 | 0.050% | Sensitivity | 1,486,729,118 | 100.0% | 154 | 38.2 | 95.6% |

**Supplementary Table 4.** Summary of whole genome sequencing QC metrics for the PROVENC3 tumour and germline cohort

| **Subject ID** | **Sample ID** | **Sample Source** | **Diagnosis** | **Total Reads** | **Mean Coverage (fold)** | **Median Insert Size (bp)** | **Duplication** | **Final Variant Count (Single Nucleotide Variants)** |
| --- | --- | --- | --- | --- | --- | --- | --- | --- |
|  |  |  |  |  |  |  |  |  |
| PROV1 | CF66175 | Normal | N/A | 950,475,876 | 39.2 | 403 | 15.4% | N/A |
| PROV1 | CF55821 | Tumour | CRC | 2,315,451,756 | 67.1 | 237 | 26.0% | 3,093 |
| PROV2 | CF66176 | Normal | N/A | 948,844,830 | 38.3 | 406 | 15.4% | N/A |
| PROV2 | CF55819 | Tumour | CRC | 2,391,660,458 | 59.5 | 209 | 28.9% | 4,474 |
| PROV3 | CF68109 | Normal | N/A | 970,388,676 | 41.4 | 408 | 11.0% | N/A |
| PROV3 | CF55817 | Tumour | CRC | 2,936,060,922 | 93.9 | 260 | 20.8% | 36,527 |
| PROV4 | CF66178 | Normal | N/A | 932,892,572 | 39.2 | 405 | 13.9% | N/A |
| PROV4 | CF55813 | Tumour | CRC | 2,754,854,538 | 90.4 | 265 | 22.5% | 5,126 |
| PROV5 | CF69837 | Normal | N/A | 941,763,872 | 38.5 | 484 | 16.3% | N/A |
| PROV5 | CF55454 | Tumour | CRC | 2,626,011,256 | 89.5 | 281 | 17.4% | 6,440 |
| PROV6 | CF69628 | Normal | N/A | 996,840,242 | 35 | 455 | 29.0% | N/A |
| PROV6 | CF55452 | Tumour | CRC | 2,859,910,394 | 105.4 | 285 | 13.4% | 3,361 |
| PROV7 | CF66704 | Normal | N/A | 938,728,564 | 38.6 | 399 | 15.2% | N/A |
| PROV7 | CF66266 | Tumour | CRC | 2,366,598,542 | 68.7 | 236 | 26.6% | 5,612 |
| PROV8 | CF69793 | Normal | N/A | 1,202,709,072 | 46 | 440 | 21.0% | N/A |
| PROV8 | CF66264 | Tumour | CRC | 2,795,669,386 | 115 | 362 | 12.0% | 48,130 |
| PROV9 | CF69677 | Normal | N/A | 1,106,594,322 | 47 | 422 | 12.4% | N/A |
| PROV9 | CF66270 | Tumour | CRC | 2,722,960,468 | 100.8 | 314 | 18.6% | 2,815 |
| PROV10 | CF69820 | Normal | N/A | 968,009,116 | 40.1 | 483 | 14.9% | N/A |
| PROV10 | CF66268 | Tumour | CRC | 2,441,958,912 | 85 | 301 | 21.3% | 4,344 |
| PROV11 | CF69694 | Normal | N/A | 1,460,527,394 | 57 | 432 | 21.0% | N/A |
| PROV11 | CF67235 | Tumour | CRC | 2,987,373,822 | 117 | 351 | 16.0% | 6,915 |
| PROV12 | CF69799 | Normal | N/A | 858,177,586 | 38 | 421 | 9.0% | N/A |
| PROV12 | CF70561 | Tumour | CRC | 2,590,323,872 | 89 | 285 | 19.0% | 18,687 |
| PROV13 | CF69806 | Normal | N/A | 984,634,466 | 40.6 | 495 | 15.3% | N/A |
| PROV13 | CF70557 | Tumour | CRC | 2,762,678,508 | 100.5 | 304 | 17.9% | 33,553 |
| PROV14 | CF69674 | Normal | N/A | 1,040,242,582 | 44.4 | 448 | 12.1% | N/A |
| PROV14 | CF70555 | Tumour | CRC | 2,747,313,416 | 87.3 | 285 | 25.4% | 7,411 |
| PROV15 | CF69809 | Normal | N/A | 1,002,875,958 | 44.1 | 512 | 11.8% | N/A |
| PROV15 | CF70553 | Tumour | CRC | 2,845,470,386 | 104.2 | 295 | 17.9% | 4,465 |
| PROV16 | CF69684 | Normal | N/A | 953,667,892 | 41.2 | 443 | 12.3% | N/A |
| PROV16 | CF70543 | Tumour | CRC | 2,721,512,780 | 96.2 | 289 | 18.8% | 5,285 |
| PROV17 | CF69686 | Normal | N/A | 953,118,800 | 42 | 416 | 10.0% | N/A |
| PROV17 | CF70547 | Tumour | CRC | 2,569,070,906 | 88 | 269 | 19.0% | 4,754 |
| PROV18 | CF66705 | Normal | N/A | 954,075,418 | 39.9 | 457 | 15.4% | N/A |
| PROV18 | CF60449 | Tumour | CRC | 2,686,340,292 | 100.8 | 330 | 18.8% | 5,704 |
| PROV19 | CF69828 | Normal | N/A | 989,766,508 | 40 | 505 | 17.7% | N/A |
| PROV19 | CF70541 | Tumour | CRC | 2,856,405,594 | 101.8 | 306 | 19.0% | 5,344 |
| PROV20 | CF66706 | Normal | N/A | 948,532,140 | 38.3 | 400 | 16.0% | N/A |
| PROV20 | CF64407 | Tumour | CRC | 2,736,051,468 | 101.1 | 334 | 19.5% | 4,544 |
| PROV21 | CF69783 | Normal | N/A | 1,464,245,576 | 61.4 | 437 | 13.8% | N/A |
| PROV21 | CF67594 | Tumour | CRC | 2,256,162,430 | 66.2 | 219 | 20.6% | 1,942 |
| PROV22 | CF66707 | Normal | N/A | 946,774,786 | 38.5 | 414 | 15.2% | N/A |
| PROV22 | CF66258 | Tumour | CRC | 2,848,761,038 | 98.8 | 295 | 20.2% | 6,482 |
| PROV23 | CF69829 | Normal | N/A | 1,041,848,924 | 45 | 526 | 11.4% | N/A |
| PROV23 | CF67590 | Tumour | CRC | 2,164,066,866 | 52.8 | 202 | 31.5% | 5,041 |
| PROV24 | CF69834 | Normal | N/A | 966,260,944 | 39.3 | 507 | 18.2% | N/A |
| PROV24 | CF67592 | Tumour | CRC | 2,647,249,632 | 95.6 | 291 | 17.7% | 27,619 |
| PROV25 | CF69835 | Normal | N/A | 966,087,932 | 39.1 | 472 | 17.4% | N/A |
| PROV25 | CF67588 | Tumour | CRC | 2,785,447,540 | 95.4 | 285 | 20.9% | 3,090 |
| PROV26 | CF69696 | Normal | N/A | 832,011,446 | 37 | 416 | 10.0% | N/A |
| PROV26 | CF67586 | Tumour | CRC | 2,293,380,606 | 66 | 224 | 24.0% | 6,390 |
| PROV27 | CF69796 | Normal | N/A | 1,062,801,656 | 47 | 427 | 9.0% | N/A |
| PROV27 | CF69206 | Tumour | CRC | 2,702,418,062 | 91 | 258 | 19.0% | 3,175 |
| PROV28 | CF69666 | Normal | N/A | 895,386,564 | 38.9 | 438 | 11.6% | N/A |
| PROV28 | CF69204 | Tumour | CRC | 2,656,533,112 | 94.3 | 286 | 17.6% | 4,546 |
| PROV29 | CF69668 | Normal | N/A | 993,769,808 | 42 | 417 | 12.4% | N/A |
| PROV29 | CF69200 | Tumour | CRC | 2,579,011,326 | 74.1 | 209 | 21.1% | 4,013 |
| PROV30 | CF69689 | Normal | N/A | 946,984,750 | 41 | 420 | 9.0% | N/A |
| PROV30 | CF69202 | Tumour | CRC | 2,689,376,330 | 94 | 280 | 17.0% | 3,541 |
| PROV31 | CF66179 | Normal | N/A | 949,404,232 | 38.8 | 405 | 16.1% | N/A |
| PROV31 | CF59388 | Tumour | CRC | 2,783,157,414 | 78.6 | 207 | 20.7% | 2,687 |
| PROV32 | CF69838 | Normal | N/A | 932,770,744 | 37.4 | 491 | 17.6% | N/A |
| PROV32 | CF59386 | Tumour | CRC | 2,576,807,188 | 77.1 | 239 | 21.6% | 14,290 |
| PROV33 | CF66180 | Normal | N/A | 1,001,838,476 | 42.2 | 415 | 12.1% | N/A |
| PROV33 | CF59384 | Tumour | CRC | 2,614,951,384 | 85.2 | 261 | 19.5% | 5,286 |
| PROV34 | CF66181 | Normal | N/A | 1,051,818,892 | 44.8 | 449 | 13.4% | N/A |
| PROV34 | CF59382 | Tumour | CRC | 2,589,242,408 | 88.3 | 277 | 18.7% | 3,613 |
| PROV35 | CF66182 | Normal | N/A | 1,068,278,256 | 45.8 | 440 | 12.6% | N/A |
| PROV35 | CF59380 | Tumour | CRC | 2,494,436,452 | 75.5 | 225 | 19.4% | 9,714 |
| PROV36 | CF69821 | Normal | N/A | 1,360,987,536 | 53 | 503 | 22.0% | N/A |
| PROV36 | CF69188 | Tumour | CRC | 2,551,035,600 | 86 | 255 | 19.0% | 3,715 |
| PROV37 | CF69830 | Normal | N/A | 1,012,376,822 | 40.5 | 493 | 19.4% | N/A |
| PROV37 | CF69186 | Tumour | CRC | 2,607,596,304 | 67.8 | 202 | 26.5% | 2,635 |
| PROV38 | CF66708 | Normal | N/A | 935,077,992 | 38.9 | 427 | 13.8% | N/A |
| PROV38 | CF66256 | Tumour | CRC | 3,175,321,240 | 75.2 | 230 | 37.8% | 3,522 |
| PROV39 | CF69798 | Normal | N/A | 867,793,908 | 37 | 447 | 11.0% | N/A |
| PROV39 | CF66250 | Tumour | CRC | 2,796,125,522 | 98 | 325 | 23.0% | 3,914 |
| PROV40 | CF66721 | Normal | N/A | 999,832,536 | 41.4 | 429 | 16.2% | N/A |
| PROV40 | CF66252 | Tumour | CRC | 2,774,402,024 | 94.9 | 297 | 22.8% | 3,735 |
| PROV41 | CF66709 | Normal | N/A | 938,371,642 | 39 | 407 | 14.7% | N/A |
| PROV41 | CF66254 | Tumour | CRC | 2,748,331,734 | 95.2 | 298 | 21.5% | 6,968 |
| PROV42 | CF69839 | Normal | N/A | 1,007,660,610 | 41.2 | 517 | 18.6% | N/A |
| PROV42 | CF55448 | Tumour | CRC | 2,575,362,562 | 83.5 | 268 | 23.5% | 5,087 |
| PROV43 | CF66183 | Normal | N/A | 935,941,424 | 38.4 | 398 | 15.3% | N/A |
| PROV43 | CF55446 | Tumour | CRC | 2,375,408,188 | 68.9 | 221 | 23.3% | 9,012 |
| PROV44 | CF66184 | Normal | N/A | 956,993,534 | 39 | 396 | 14.5% | N/A |
| PROV44 | CF55444 | Tumour | CRC | 2,357,135,784 | 60 | 211 | 28.0% | 17,101 |
| PROV45 | CF66221 | Normal | N/A | 948,371,488 | 38.2 | 403 | 16.5% | N/A |
| PROV45 | CF55442 | Tumour | CRC | 2,749,087,132 | 91.5 | 270 | 20.6% | 4,362 |
| PROV46 | CF69779 | Normal | N/A | 1,197,044,498 | 51 | 407 | 11.0% | N/A |
| PROV46 | CF66244 | Tumour | CRC | 2,551,408,646 | 60 | 180 | 29.0% | 19,918 |
| PROV47 | CF69807 | Normal | N/A | 1,127,242,056 | 44.1 | 497 | 20.7% | N/A |
| PROV47 | CF66248 | Tumour | CRC | 2,567,105,534 | 84.3 | 286 | 23.0% | 5,961 |
| PROV48 | CF69788 | Normal | N/A | 991,723,970 | 42.8 | 455 | 12.2% | N/A |
| PROV48 | CF69212 | Tumour | CRC | 2,546,685,194 | 74.8 | 234 | 25.1% | 5,634 |
| PROV49 | CF69794 | Normal | N/A | 1,056,060,692 | 46 | 458 | 11.0% | N/A |
| PROV49 | CF69210 | Tumour | CRC | 2,555,740,442 | 76 | 256 | 27.0% | 31,121 |
| PROV50 | CF69797 | Normal | N/A | 997,735,614 | 43 | 455 | 11.0% | N/A |
| PROV50 | CF69208 | Tumour | CRC | 2,852,648,452 | 94 | 292 | 24.0% | 4,627 |
| PROV51 | CF69801 | Normal | N/A | 928,912,814 | 39.1 | 418 | 13.0% | N/A |
| PROV51 | CF69226 | Tumour | CRC | 2,230,023,928 | 57.2 | 190 | 21.7% | 21,514 |
| PROV52 | CF69669 | Normal | N/A | 1,164,583,836 | 50.3 | 466 | 12.3% | N/A |
| PROV52 | CF69230 | Tumour | CRC | 2,667,605,998 | 85 | 270 | 23.4% | 8,140 |
| PROV53 | CF69811 | Normal | N/A | 1,235,475,026 | 48.4 | 509 | 19.6% | N/A |
| PROV53 | CF69228 | Tumour | CRC | 2,492,918,486 | 82.7 | 292 | 20.8% | 6,290 |
| PROV54 | CF69826 | Normal | N/A | 959,302,058 | 41.9 | 504 | 12.5% | N/A |
| PROV54 | CF70503 | Tumour | CRC | 2,865,954,658 | 93 | 284 | 23.6% | 5,242 |
| PROV55 | CF69840 | Normal | N/A | 997,362,202 | 43.2 | 532 | 11.8% | N/A |
| PROV55 | CF70797 | Tumour | CRC | 2,726,466,042 | 83.1 | 252 | 24.1% | 14,180 |
| PROV56 | CF66185 | Normal | N/A | 946,784,636 | 39.9 | 412 | 14.8% | N/A |
| PROV56 | CF60114 | Tumour | CRC | 2,794,067,160 | 100 | 308 | 19.7% | 4,823 |
| PROV57 | CF69638 | Normal | N/A | 1,216,221,506 | 45.8 | 415 | 22.0% | N/A |
| PROV57 | CF64598 | Tumour | CRC | 2,667,688,288 | 82.2 | 253 | 22.8% | 3,539 |
| PROV58 | CF70677 | Normal | N/A | 1,171,746,868 | 50.9 | 483 | 10.7% | N/A |
| PROV58 | CF64519 | Tumour | CRC | 2,732,302,158 | 76.4 | 222 | 24.2% | 6,790 |
| PROV59 | CF66186 | Normal | N/A | 944,546,344 | 39 | 417 | 15.6% | N/A |
| PROV59 | CF55462 | Tumour | CRC | 2,807,733,806 | 93 | 273 | 22.6% | 6,152 |
| PROV60 | CF66187 | Normal | N/A | 950,390,868 | 38.7 | 416 | 17.0% | N/A |
| PROV60 | CF55460 | Tumour | CRC | 2,323,658,270 | 69.6 | 257 | 25.4% | 3,927 |
| PROV61 | CF69626 | Normal | N/A | 928,852,644 | 40 | 419 | 12.4% | N/A |
| PROV61 | CF55456 | Tumour | CRC | 2,815,340,104 | 100.8 | 274 | 16.2% | 44,521 |
| PROV62 | CF69698 | Normal | N/A | 1,029,972,040 | 44.6 | 449 | 12.3% | N/A |
| PROV62 | CF55458 | Tumour | CRC | 2,868,579,918 | 98.9 | 313 | 22.6% | 3,989 |
| PROV63 | CF69699 | Normal | N/A | 989,228,026 | 41.8 | 432 | 12.7% | N/A |
| PROV63 | CF63889 | Tumour | CRC | 2,455,996,498 | 83.8 | 298 | 21.9% | 4,718 |
| PROV64 | CF69700 | Normal | N/A | 1,002,726,216 | 41.5 | 513 | 15.5% | N/A |
| PROV64 | CF62918 | Tumour | CRC | 2,558,483,928 | 64.2 | 219 | 29.9% | 3,983 |
| PROV65 | CF66188 | Normal | N/A | 941,297,418 | 39.9 | 429 | 14.3% | N/A |
| PROV65 | CF64296 | Tumour | CRC | 2,836,278,278 | 89.8 | 260 | 23.0% | 27,698 |
| PROV66 | CF66223 | Normal | N/A | 931,946,204 | 38.4 | 411 | 15.0% | N/A |
| PROV66 | CF64292 | Tumour | CRC | 2,794,690,596 | 96.1 | 297 | 21.6% | 3,402 |
| PROV67 | CF66189 | Normal | N/A | 942,636,190 | 38.4 | 400 | 15.5% | N/A |
| PROV67 | CF63883 | Tumour | CRC | 3,389,107,792 | 69.2 | 207 | 42.7% | 6,634 |
| PROV68 | CF66224 | Normal | N/A | 946,591,402 | 39.1 | 392 | 13.6% | N/A |
| PROV68 | CF63881 | Tumour | CRC | 2,335,212,624 | 72.1 | 279 | 26.3% | 5,403 |
| PROV69 | CF69778 | Normal | N/A | 982,181,512 | 40.4 | 443 | 15.9% | N/A |
| PROV69 | CF66234 | Tumour | CRC | 2,639,360,732 | 67.3 | 212 | 30.1% | 3,977 |
| PROV70 | CF69812 | Normal | N/A | 945,015,154 | 40.5 | 478 | 12.1% | N/A |
| PROV70 | CF69198 | Tumour | CRC | 2,806,525,616 | 98.1 | 319 | 22.2% | 23,456 |
| PROV71 | CF69621 | Normal | N/A | 1,043,992,058 | 44.1 | 450 | 12.6% | N/A |
| PROV71 | CF56924 | Tumour | CRC | 2,525,032,466 | 78.3 | 253 | 23.0% | 3,674 |
| PROV72 | CF70678 | Normal | N/A | 1,124,251,464 | 49.5 | 485 | 10.9% | N/A |
| PROV72 | CF56912 | Tumour | CRC | 2,747,762,570 | 99.2 | 340 | 21.5% | 6,703 |
| PROV73 | CF69786 | Normal | N/A | 1,000,765,912 | 42.2 | 502 | 14.5% | N/A |
| PROV73 | CF70493 | Tumour | CRC | 2,745,902,696 | 81.4 | 250 | 25.3% | 4,056 |
| PROV74 | CF69824 | Normal | N/A | 1,252,400,038 | 55.8 | 500 | 10.7% | N/A |
| PROV74 | CF67556 | Tumour | CRC | 2,737,432,312 | 93.3 | 296 | 23.3% | 3,619 |
| PROV75 | CF69692 | Normal | N/A | 971,002,708 | 39.9 | 480 | 16.8% | N/A |
| PROV75 | CF70491 | Tumour | CRC | 2,628,622,940 | 69.6 | 224 | 30.0% | 3,552 |
| PROV76 | CF69836 | Normal | N/A | 1,038,613,106 | 45 | 490 | 11.9% | N/A |
| PROV76 | CF69190 | Tumour | CRC | 2,340,981,614 | 88.2 | 351 | 20.2% | 3,885 |
| PROV77 | CF66190 | Normal | N/A | 945,275,452 | 39.6 | 402 | 14.3% | N/A |
| PROV77 | CF56936 | Tumour | CRC | 2,827,589,610 | 95.9 | 275 | 19.6% | 49,685 |
| PROV78 | CF66225 | Normal | N/A | 936,916,810 | 37 | 380 | 16.4% | N/A |
| PROV78 | CF56938 | Tumour | CRC | 2,742,919,592 | 91.5 | 281 | 21.6% | 5,882 |
| PROV79 | CF69757 | Normal | N/A | 1,077,511,734 | 44.2 | 485 | 15.4% | N/A |
| PROV79 | CF56940 | Tumour | CRC | 2,765,566,838 | 89.1 | 279 | 24.1% | 51,581 |
| PROV80 | CF66191 | Normal | N/A | 1,041,218,508 | 36.3 | 413 | 27.5% | N/A |
| PROV80 | CF56942 | Tumour | CRC | 2,708,739,150 | 67.5 | 206 | 28.8% | 4,258 |
| PROV81 | CF66226 | Normal | N/A | 944,095,830 | 38.4 | 405 | 15.1% | N/A |
| PROV81 | CF56880 | Tumour | CRC | 2,740,400,024 | 96 | 286 | 18.8% | 16,475 |
| PROV82 | CF66192 | Normal | N/A | 809,164,556 | 35.4 | 386 | 8.1% | N/A |
| PROV82 | CF56944 | Tumour | CRC | 3,302,209,328 | 76.4 | 224 | 38.3% | 4,063 |
| PROV83 | CF69625 | Normal | N/A | 1,188,282,172 | 40.7 | 442 | 29.0% | N/A |
| PROV83 | CF61787 | Tumour | CRC | 2,827,227,652 | 65.4 | 202 | 32.3% | 31,912 |
| PROV84 | CF69762 | Normal | N/A | 925,247,814 | 38.4 | 470 | 15.6% | N/A |
| PROV84 | CF61789 | Tumour | CRC | 2,519,577,808 | 58.3 | 198 | 33.0% | 3,837 |
| PROV85 | CF69764 | Normal | N/A | 1,056,005,026 | 43.7 | 488 | 15.1% | N/A |
| PROV85 | CF61959 | Tumour | CRC | 2,785,183,868 | 91.3 | 282 | 23.1% | 6,523 |
| PROV86 | CF69631 | Normal | N/A | 1,043,624,002 | 44.1 | 430 | 12.6% | N/A |
| PROV86 | CF61793 | Tumour | CRC | 2,587,920,442 | 81.4 | 272 | 23.7% | 4,532 |
| PROV87 | CF69769 | Normal | N/A | 1,069,843,288 | 43.4 | 479 | 15.9% | N/A |
| PROV87 | CF61799 | Tumour | CRC | 2,804,311,946 | 85.8 | 262 | 25.1% | 2,647 |
| PROV88 | CF69640 | Normal | N/A | 1,100,474,492 | 47.8 | 457 | 12.4% | N/A |
| PROV88 | CF61803 | Tumour | CRC | 2,687,786,100 | 81.4 | 248 | 24.3% | 4,065 |
| PROV89 | CF69777 | Normal | N/A | 937,725,006 | 38.2 | 484 | 16.0% | N/A |
| PROV89 | CF61291 | Tumour | CRC | 2,741,363,150 | 88.1 | 294 | 24.2% | 6,683 |
| PROV90 | CF70679 | Normal | N/A | 970,829,648 | 41 | 486 | 13.3% | N/A |
| PROV90 | CF61293 | Tumour | CRC | 2,442,641,632 | 74.8 | 257 | 24.4% | 3,495 |
| PROV91 | CF69767 | Normal | N/A | 955,837,620 | 38.8 | 457 | 16.8% | N/A |
| PROV91 | CF61295 | Tumour | CRC | 2,741,224,938 | 89.6 | 285 | 24.2% | 3,350 |
| PROV92 | CF70674 | Normal | N/A | 968,853,748 | 42.6 | 491 | 11.5% | N/A |
| PROV92 | CF67245 | Tumour | CRC | 2,717,859,814 | 88.3 | 293 | 25.4% | 3,872 |
| PROV93 | CF70675 | Normal | N/A | 1,013,080,672 | 44.9 | 546 | 11.1% | N/A |
| PROV93 | CF67243 | Tumour | CRC | 2,679,880,090 | 68.2 | 221 | 31.8% | 4,885 |
| PROV94 | CF69646 | Normal | N/A | 869,292,798 | 37.1 | 446 | 11.8% | N/A |
| PROV94 | CF61925 | Tumour | CRC | 2,494,100,178 | 44 | 149 | 35.6% | 3,298 |
| PROV95 | CF66711 | Normal | N/A | 847,345,162 | 36.7 | 382 | 9.1% | N/A |
| PROV95 | CF60415 | Tumour | CRC | 3,619,511,054 | 88.2 | 242 | 38.5% | 6,520 |
| PROV96 | CF66722 | Normal | N/A | 820,040,784 | 36.1 | 414 | 8.8% | N/A |
| PROV96 | CF60443 | Tumour | CRC | 2,781,423,704 | 91.6 | 272 | 20.3% | 3,543 |
| PROV97 | CF66193 | Normal | N/A | 823,456,590 | 36.2 | 378 | 9.2% | N/A |
| PROV97 | CF56910 | Tumour | CRC | 2,344,151,636 | 64.3 | 226 | 28.5% | 2,448 |
| PROV98 | CF66194 | Normal | N/A | 804,360,110 | 35.9 | 385 | 8.1% | N/A |
| PROV98 | CF56908 | Tumour | CRC | 2,360,277,388 | 70.2 | 237 | 24.3% | 10,396 |
| PROV99 | CF66195 | Normal | N/A | 812,914,682 | 36.1 | 388 | 9.0% | N/A |
| PROV99 | CF56904 | Tumour | CRC | 3,275,193,262 | 67.9 | 194 | 37.9% | 4,019 |
| PROV100 | CF69756 | Normal | N/A | 1,059,928,226 | 44.3 | 485 | 15.4% | N/A |
| PROV100 | CF55827 | Tumour | CRC | 2,622,411,872 | 80.5 | 256 | 25.1% | 5,689 |
| PROV101 | CF68111 | Normal | N/A | 962,990,164 | 42.3 | 392 | 9.9% | N/A |
| PROV101 | CF56906 | Tumour | CRC | 2,746,164,624 | 49.2 | 154 | 37.5% | 4,512 |
| PROV102 | CF69622 | Normal | N/A | 891,978,588 | 37 | 418 | 14.0% | N/A |
| PROV102 | CF55825 | Tumour | CRC | 2,441,966,512 | 72.8 | 247 | 25.4% | 3,939 |
| PROV103 | CF69760 | Normal | N/A | 992,981,378 | 40.6 | 493 | 16.1% | N/A |
| PROV103 | CF55829 | Tumour | CRC | 2,611,419,902 | 77.8 | 255 | 26.4% | 36,420 |
| PROV104 | CF69768 | Normal | N/A | 1,278,149,734 | 52.3 | 485 | 15.7% | N/A |
| PROV104 | CF62914 | Tumour | CRC | 2,679,851,972 | 61.7 | 200 | 32.3% | 3,761 |
| PROV105 | CF69701 | Normal | N/A | 1,051,126,142 | 42.2 | 452 | 16.0% | N/A |
| PROV105 | CF62157 | Tumour | CRC | 2,559,179,332 | 72.3 | 234 | 25.8% | 25,401 |
| PROV106 | CF69772 | Normal | N/A | 990,018,504 | 39.7 | 465 | 17.7% | N/A |
| PROV106 | CF62161 | Tumour | CRC | 2,511,225,840 | 52.9 | 174 | 32.6% | 11,415 |
| PROV107 | CF69773 | Normal | N/A | 978,538,884 | 40.7 | 494 | 15.8% | N/A |
| PROV107 | CF62910 | Tumour | CRC | 2,431,281,150 | 78.9 | 279 | 24.5% | 11,658 |
| PROV108 | CF66197 | Normal | N/A | 802,550,694 | 35.6 | 415 | 8.0% | N/A |
| PROV108 | CF62167 | Tumour | CRC | 2,821,696,550 | 79 | 216 | 22.5% | 2,738 |
| PROV109 | CF70680 | Normal | N/A | 1,066,001,018 | 46.4 | 540 | 11.6% | N/A |
| PROV109 | CF62165 | Tumour | CRC | 2,439,854,564 | 73.1 | 219 | 17.8% | 37,639 |
| PROV110 | CF69647 | Normal | N/A | 1,212,041,716 | 51.7 | 456 | 12.0% | N/A |
| PROV110 | CF66232 | Tumour | CRC | 2,405,453,828 | 75.4 | 249 | 18.6% | 2,361 |
| PROV111 | CF69810 | Normal | N/A | 1,226,912,874 | 52.3 | 520 | 13.0% | N/A |
| PROV111 | CF56900 | Tumour | CRC | 2,557,920,896 | 99.3 | 333 | 15.1% | 32,893 |
| PROV112 | CF69758 | Normal | N/A | 968,517,612 | 40.8 | 493 | 14.9% | N/A |
| PROV112 | CF56934 | Tumour | CRC | 2,749,930,204 | 64.3 | 187 | 29.6% | 3,515 |
| PROV113 | CF66198 | Normal | N/A | 823,037,974 | 35.6 | 385 | 9.7% | N/A |
| PROV113 | CF56918 | Tumour | CRC | 2,795,659,808 | 87.3 | 258 | 23.0% | 3,227 |
| PROV114 | CF66227 | Normal | N/A | 1,250,276,778 | 54.4 | 451 | 12.5% | N/A |
| PROV114 | CF56916 | Tumour | CRC | 2,481,936,356 | 87.5 | 263 | 15.3% | 7,107 |
| PROV115 | CF66199 | Normal | N/A | 816,118,258 | 36.7 | 386 | 8.3% | N/A |
| PROV115 | CF56930 | Tumour | CRC | 2,795,818,304 | 88.8 | 251 | 22.4% | 7,271 |
| PROV116 | CF69759 | Normal | N/A | 936,158,266 | 38.4 | 483 | 16.8% | N/A |
| PROV116 | CF56886 | Tumour | CRC | 2,711,024,758 | 56.2 | 172 | 33.5% | 2,599 |
| PROV117 | CF69761 | Normal | N/A | 1,009,242,132 | 42.4 | 473 | 15.2% | N/A |
| PROV117 | CF56884 | Tumour | CRC | 2,691,049,104 | 64 | 175 | 24.5% | 4,790 |
| PROV118 | CF69624 | Normal | N/A | 990,643,064 | 43.1 | 445 | 12.0% | N/A |
| PROV118 | CF56932 | Tumour | CRC | 2,551,537,642 | 90 | 259 | 14.3% | 3,194 |
| PROV119 | CF69702 | Normal | N/A | 954,009,892 | 39 | 481 | 17.4% | N/A |
| PROV119 | CF56882 | Tumour | CRC | 2,620,235,876 | 92.3 | 267 | 16.5% | 3,712 |
| PROV120 | CF69703 | Normal | N/A | 1,019,507,572 | 42.4 | 464 | 15.6% | N/A |
| PROV120 | CF56920 | Tumour | CRC | 2,967,034,818 | 101.1 | 256 | 16.7% | 6,976 |
| PROV121 | CF69704 | Normal | N/A | 995,539,416 | 40.1 | 457 | 16.9% | N/A |
| PROV121 | CF56922 | Tumour | CRC | 2,703,356,058 | 94.2 | 277 | 17.4% | 5,986 |
| PROV122 | CF69634 | Normal | N/A | 986,550,642 | 39.8 | 434 | 18.7% | N/A |
| PROV122 | CF61277 | Tumour | CRC | 2,803,588,092 | 100.4 | 275 | 16.4% | 2,963 |
| PROV123 | CF69770 | Normal | N/A | 1,050,188,630 | 43.6 | 478 | 15.8% | N/A |
| PROV123 | CF61273 | Tumour | CRC | 2,748,481,948 | 93.4 | 270 | 18.7% | 5,984 |
| PROV124 | CF69635 | Normal | N/A | 917,984,426 | 39.6 | 441 | 12.5% | N/A |
| PROV124 | CF61285 | Tumour | CRC | 3,015,288,176 | 53.6 | 159 | 39.0% | 5,913 |
| PROV125 | CF69771 | Normal | N/A | 1,022,939,264 | 41 | 453 | 17.2% | N/A |
| PROV125 | CF61283 | Tumour | CRC | 2,519,650,010 | 84.6 | 247 | 16.6% | 2,972 |
| PROV126 | CF69774 | Normal | N/A | 991,443,284 | 40.4 | 482 | 15.6% | N/A |
| PROV126 | CF68313 | Tumour | CRC | 2,751,152,808 | 92 | 252 | 16.4% | 2,515 |
| PROV127 | CF66200 | Normal | N/A | 821,395,340 | 36.4 | 403 | 8.1% | N/A |
| PROV127 | CF61279 | Tumour | CRC | 2,757,520,290 | 85.7 | 261 | 24.4% | 3,953 |
| PROV128 | CF66201 | Normal | N/A | 828,686,106 | 36.7 | 392 | 8.7% | N/A |
| PROV128 | CF61275 | Tumour | CRC | 2,865,250,542 | 89.3 | 259 | 23.5% | 15,336 |
| PROV129 | CF66202 | Normal | N/A | 846,920,834 | 37.4 | 385 | 9.1% | N/A |
| PROV129 | CF61281 | Tumour | CRC | 2,321,792,590 | 71.1 | 255 | 25.3% | 2,423 |
| PROV130 | CF69792 | Normal | N/A | 935,390,658 | 37.9 | 478 | 16.8% | N/A |
| PROV130 | CF67241 | Tumour | CRC | 2,654,478,658 | 71 | 195 | 20.9% | 2,478 |
| PROV131 | CF69816 | Normal | N/A | 956,664,796 | 42.1 | 539 | 11.8% | N/A |
| PROV131 | CF67239 | Tumour | CRC | 2,660,401,980 | 98.1 | 289 | 15.9% | 4,101 |
| PROV132 | CF69804 | Normal | N/A | 918,424,872 | 40.1 | 529 | 12.1% | N/A |
| PROV132 | CF70302 | Tumour | CRC | 2,525,920,406 | 91.8 | 280 | 15.4% | 2,183 |
| PROV133 | CF69675 | Normal | N/A | 1,251,963,766 | 52.9 | 440 | 12.6% | N/A |
| PROV133 | CF70288 | Tumour | CRC | 2,610,939,100 | 95.2 | 292 | 15.5% | 49,659 |
| PROV134 | CF69822 | Normal | N/A | 1,162,983,902 | 51.1 | 508 | 11.9% | N/A |
| PROV134 | CF70284 | Tumour | CRC | 2,836,670,034 | 92.9 | 235 | 17.1% | 46,728 |
| PROV135 | CF66714 | Normal | N/A | 821,283,748 | 36.9 | 399 | 8.5% | N/A |
| PROV135 | CF60455 | Tumour | CRC | 2,738,669,266 | 101.2 | 324 | 19.4% | 9,870 |
| PROV136 | CF66203 | Normal | N/A | 812,899,704 | 35.7 | 386 | 8.9% | N/A |
| PROV136 | CF60106 | Tumour | CRC | 2,694,405,290 | 88.8 | 274 | 22.4% | 4,469 |
| PROV137 | CF69843 | Normal | N/A | 1,100,047,384 | 47.7 | 538 | 12.2% | N/A |
| PROV137 | CF70799 | Tumour | CRC | 2,619,380,062 | 87.1 | 255 | 16.3% | 3,718 |
| PROV138 | CF66204 | Normal | N/A | 822,281,690 | 35.6 | 412 | 9.0% | N/A |
| PROV138 | CF60108 | Tumour | CRC | 2,742,312,158 | 83.8 | 245 | 22.5% | 8,673 |
| PROV139 | CF66205 | Normal | N/A | 815,203,422 | 35.7 | 383 | 8.9% | N/A |
| PROV139 | CF60104 | Tumour | CRC | 2,799,228,560 | 95.6 | 287 | 21.2% | 5,389 |
| PROV140 | CF66206 | Normal | N/A | 824,903,676 | 35.8 | 409 | 10.2% | N/A |
| PROV140 | CF60112 | Tumour | CRC | 2,841,759,352 | 93.5 | 263 | 21.8% | 8,453 |
| PROV141 | CF66207 | Normal | N/A | 800,430,890 | 35.7 | 401 | 8.8% | N/A |
| PROV141 | CF59967 | Tumour | CRC | 2,724,574,208 | 90.7 | 274 | 22.2% | 11,449 |
| PROV142 | CF66208 | Normal | N/A | 819,580,024 | 35.7 | 398 | 9.0% | N/A |
| PROV142 | CF60110 | Tumour | CRC | 2,364,675,616 | 72.8 | 263 | 25.8% | 5,653 |
| PROV143 | CF69705 | Normal | N/A | 933,883,338 | 39.2 | 510 | 15.2% | N/A |
| PROV143 | CF60100 | Tumour | CRC | 2,549,950,462 | 96.3 | 306 | 15.9% | 5,412 |
| PROV144 | CF69627 | Normal | N/A | 1,139,589,552 | 39.2 | 450 | 29.0% | N/A |
| PROV144 | CF60102 | Tumour | CRC | 2,695,507,696 | 93.5 | 290 | 19.1% | 4,023 |
| PROV145 | CF69765 | Normal | N/A | 961,399,004 | 39.3 | 484 | 15.9% | N/A |
| PROV145 | CF70569 | Tumour | CRC | 2,754,669,310 | 96.3 | 276 | 16.0% | 6,898 |
| PROV146 | CF70676 | Normal | N/A | 970,818,896 | 42.4 | 521 | 12.2% | N/A |
| PROV146 | CF64594 | Tumour | CRC | 2,568,504,524 | 93.3 | 283 | 16.3% | 7,663 |
| PROV147 | CF66209 | Normal | N/A | 831,202,194 | 36.5 | 385 | 8.8% | N/A |
| PROV147 | CF64546 | Tumour | CRC | 2,327,903,358 | 74.6 | 281 | 24.7% | 3,063 |
| PROV148 | CF70681 | Normal | N/A | 949,678,280 | 41.8 | 535 | 11.6% | N/A |
| PROV148 | CF70801 | Tumour | CRC | 2,760,057,958 | 97.7 | 281 | 17.6% | 4,820 |
| PROV149 | CF69648 | Normal | N/A | 1,267,213,062 | 53.9 | 444 | 12.2% | N/A |
| PROV149 | CF70567 | Tumour | CRC | 2,633,725,632 | 95.9 | 286 | 15.4% | 4,265 |
| PROV150 | CF69645 | Normal | N/A | 919,335,522 | 39.5 | 450 | 12.8% | N/A |
| PROV150 | CF70539 | Tumour | CRC | 2,885,932,716 | 95.2 | 254 | 19.1% | 4,436 |
| PROV151 | CF69656 | Normal | N/A | 925,127,230 | 39.5 | 446 | 13.3% | N/A |
| PROV151 | CF70565 | Tumour | CRC | 2,762,605,174 | 101.3 | 285 | 15.8% | 2,890 |
| PROV152 | CF69663 | Normal | N/A | 940,226,440 | 40.2 | 438 | 11.9% | N/A |
| PROV152 | CF70563 | Tumour | CRC | 2,628,120,296 | 100.3 | 309 | 14.6% | 6,083 |
| PROV153 | CF69681 | Normal | N/A | 1,382,794,192 | 52.8 | 425 | 21.4% | N/A |
| PROV153 | CF70551 | Tumour | CRC | 2,690,273,460 | 102.4 | 315 | 14.4% | 8,055 |
| PROV154 | CF66715 | Normal | N/A | 825,070,406 | 35.9 | 403 | 9.5% | N/A |
| PROV154 | CF56902 | Tumour | CRC | 2,654,962,238 | 96 | 327 | 18.6% | 5,213 |
| PROV155 | CF69818 | Normal | N/A | 910,332,778 | 39.8 | 516 | 11.7% | N/A |
| PROV155 | CF70545 | Tumour | CRC | 2,596,790,316 | 99.8 | 310 | 14.9% | 4,279 |
| PROV156 | CF66716 | Normal | N/A | 815,143,110 | 35.8 | 401 | 9.3% | N/A |
| PROV156 | CF60433 | Tumour | CRC | 2,652,577,258 | 99.8 | 334 | 19.0% | 3,366 |
| PROV157 | CF68112 | Normal | N/A | 974,425,660 | 41.5 | 407 | 10.6% | N/A |
| PROV157 | CF60447 | Tumour | CRC | 2,752,695,992 | 100.2 | 307 | 14.7% | 4,482 |
| PROV158 | CF66718 | Normal | N/A | 813,926,806 | 23.3 | 199 | 15.6% | N/A |
| PROV158 | CF60451 | Tumour | CRC | 2,810,785,000 | 98.8 | 299 | 20.6% | 4,397 |
| PROV159 | CF66211 | Normal | N/A | 830,107,106 | 36.1 | 382 | 9.6% | N/A |
| PROV159 | CF55536 | Tumour | CRC | 2,812,454,260 | 90.6 | 262 | 21.7% | 5,058 |
| PROV160 | CF66212 | Normal | N/A | 799,783,358 | 35.7 | 397 | 8.8% | N/A |
| PROV160 | CF55464 | Tumour | CRC | 2,698,616,824 | 88.3 | 270 | 22.9% | 6,711 |
| PROV161 | CF66213 | Normal | N/A | 797,751,832 | 35.3 | 388 | 8.9% | N/A |
| PROV161 | CF64308 | Tumour | CRC | 2,254,451,698 | 67.1 | 244 | 25.4% | 3,692 |
| PROV162 | CF69784 | Normal | N/A | 998,717,500 | 42 | 485 | 15.0% | N/A |
| PROV162 | CF70300 | Tumour | CRC | 2,910,355,362 | 104 | 264 | 15.0% | 38,484 |
| PROV163 | CF69781 | Normal | N/A | 996,843,532 | 40.6 | 472 | 15.9% | N/A |
| PROV163 | CF70296 | Tumour | CRC | 2,893,372,912 | 96.6 | 257 | 16.3% | 3,593 |
| PROV164 | CF69791 | Normal | N/A | 971,583,624 | 39.8 | 494 | 15.8% | N/A |
| PROV164 | CF70292 | Tumour | CRC | 2,712,677,882 | 98.2 | 286 | 14.7% | 6,390 |
| PROV165 | CF69665 | Normal | N/A | 936,528,048 | 39.3 | 431 | 12.9% | N/A |
| PROV165 | CF70290 | Tumour | CRC | 2,642,021,242 | 97 | 295 | 15.5% | 31,432 |
| PROV166 | CF69800 | Normal | N/A | 1,010,100,752 | 42.4 | 493 | 13.4% | N/A |
| PROV166 | CF67233 | Tumour | CRC | 2,629,719,814 | 98 | 295 | 14.6% | 40,689 |
| PROV167 | CF69683 | Normal | N/A | 933,543,400 | 39.9 | 451 | 12.0% | N/A |
| PROV167 | CF67229 | Tumour | CRC | 2,569,061,060 | 100.1 | 314 | 13.0% | 83,033 |
| PROV168 | CF69832 | Normal | N/A | 956,237,398 | 41 | 514 | 12.3% | N/A |
| PROV168 | CF70306 | Tumour | CRC | 2,940,073,426 | 103.6 | 269 | 15.8% | 2,961 |
| PROV169 | CF69691 | Normal | N/A | 1,006,142,728 | 41.8 | 494 | 15.8% | N/A |
| PROV169 | CF70294 | Tumour | CRC | 2,631,916,472 | 101.3 | 301 | 13.3% | 5,897 |
| PROV170 | CF69693 | Normal | N/A | 984,011,496 | 39.4 | 488 | 18.1% | N/A |
| PROV170 | CF70304 | Tumour | CRC | 2,492,268,346 | 100.5 | 329 | 12.6% | 6,716 |
| PROV171 | CF66723 | Normal | N/A | 815,734,036 | 35.5 | 381 | 8.9% | N/A |
| PROV171 | CF66402 | Tumour | CRC | 2,815,811,562 | 102.1 | 319 | 19.0% | 5,598 |
| PROV172 | CF69817 | Normal | N/A | 1,035,282,554 | 45.8 | 528 | 11.2% | N/A |
| PROV172 | CF69194 | Tumour | CRC | 2,638,148,656 | 100 | 296 | 15.2% | 121,896 |
| PROV173 | CF69695 | Normal | N/A | 904,911,664 | 38.2 | 484 | 15.1% | N/A |
| PROV173 | CF70495 | Tumour | CRC | 2,590,676,912 | 100.9 | 316 | 14.1% | 9,395 |
| PROV174 | CF69782 | Normal | N/A | 944,512,100 | 39.4 | 498 | 15.5% | N/A |
| PROV174 | CF69224 | Tumour | CRC | 3,095,160,318 | 83.9 | 205 | 22.7% | 6,123 |
| PROV175 | CF69659 | Normal | N/A | 1,022,763,490 | 44 | 440 | 12.6% | N/A |
| PROV175 | CF66242 | Tumour | CRC | 2,737,019,364 | 101.2 | 291 | 16.4% | 3,313 |
| PROV176 | CF66719 | Normal | N/A | 820,255,092 | 35.8 | 408 | 8.8% | N/A |
| PROV176 | CF66240 | Tumour | CRC | 2,328,145,758 | 71.3 | 265 | 25.2% | 4,719 |
| PROV177 | CF69789 | Normal | N/A | 980,494,554 | 42 | 499 | 12.3% | N/A |
| PROV177 | CF66238 | Tumour | CRC | 2,758,888,632 | 101.6 | 282 | 15.0% | 40,133 |
| PROV178 | CF69661 | Normal | N/A | 1,107,522,174 | 47.1 | 437 | 12.3% | N/A |
| PROV178 | CF69222 | Tumour | CRC | 2,600,668,718 | 97.4 | 307 | 14.7% | 4,262 |
| PROV179 | CF69652 | Normal | N/A | 1,314,895,524 | 57.3 | 457 | 12.4% | N/A |
| PROV179 | CF67227 | Tumour | CRC | 2,613,346,610 | 97.9 | 293 | 15.0% | 12,827 |
| PROV180 | CF69787 | Normal | N/A | 952,521,554 | 41.3 | 513 | 12.7% | N/A |
| PROV180 | CF67223 | Tumour | CRC | 2,583,077,832 | 101.4 | 310 | 13.4% | 5,072 |
| PROV181 | CF69660 | Normal | N/A | 1,073,400,928 | 46.2 | 425 | 12.7% | N/A |
| PROV181 | CF67221 | Tumour | CRC | 2,624,104,486 | 100.9 | 306 | 14.8% | 4,672 |
| PROV182 | CF69805 | Normal | N/A | 970,175,304 | 41.5 | 535 | 12.6% | N/A |
| PROV182 | CF67566 | Tumour | CRC | 2,490,826,854 | 96.8 | 307 | 13.3% | 7,512 |
| PROV183 | CF69678 | Normal | N/A | 992,517,876 | 42.1 | 438 | 12.3% | N/A |
| PROV183 | CF67564 | Tumour | CRC | 2,514,594,260 | 95.5 | 314 | 13.2% | 2,900 |
| PROV184 | CF69808 | Normal | N/A | 1,001,191,314 | 43.3 | 555 | 11.5% | N/A |
| PROV184 | CF67560 | Tumour | CRC | 2,563,796,756 | 100.7 | 315 | 13.5% | 5,883 |
| PROV185 | CF69680 | Normal | N/A | 1,105,155,032 | 44 | 437 | 19.0% | N/A |
| PROV185 | CF67562 | Tumour | CRC | 2,928,231,588 | 107 | 284 | 16.0% | 3,369 |
| PROV186 | CF69688 | Normal | N/A | 973,999,330 | 43.2 | 517 | 11.4% | N/A |
| PROV186 | CF67558 | Tumour | CRC | 2,520,310,722 | 88.9 | 271 | 17.2% | 7,057 |
| PROV187 | CF66228 | Normal | N/A | 803,860,070 | 35.9 | 396 | 9.3% | N/A |
| PROV187 | CF59945 | Tumour | CRC | 2,314,846,858 | 67.5 | 240 | 25.9% | 6,115 |
| PROV188 | CF66215 | Normal | N/A | 792,080,046 | 34.7 | 384 | 9.4% | N/A |
| PROV188 | CF59374 | Tumour | CRC | 2,328,998,114 | 75.5 | 275 | 23.8% | 3,776 |
| PROV189 | CF66216 | Normal | N/A | 789,503,742 | 34.7 | 389 | 9.2% | N/A |
| PROV189 | CF59378 | Tumour | CRC | 3,556,533,858 | 84.2 | 219 | 35.1% | 5,278 |
| PROV190 | CF69844 | Normal | N/A | 995,297,850 | 43.6 | 518 | 11.4% | N/A |
| PROV190 | CF59947 | Tumour | CRC | 2,709,749,290 | 96.7 | 277 | 14.8% | 2,405 |
| PROV191 | CF66229 | Normal | N/A | 819,218,152 | 35.6 | 398 | 9.1% | N/A |
| PROV191 | CF59372 | Tumour | CRC | 2,328,610,730 | 69.3 | 249 | 24.5% | 6,193 |
| PROV192 | CF66230 | Normal | N/A | 793,928,600 | 34.9 | 402 | 8.8% | N/A |
| PROV192 | CF59961 | Tumour | CRC | 3,313,508,916 | 67.4 | 213 | 43.4% | 3,978 |
| PROV193 | CF66217 | Normal | N/A | 1,116,803,532 | 44 | 429 | 20.0% | N/A |
| PROV193 | CF59955 | Tumour | CRC | 2,783,149,702 | 96 | 264 | 16.0% | 72,994 |
| PROV194 | CF66231 | Normal | N/A | 883,626,982 | 39 | 431 | 11.0% | N/A |
| PROV194 | CF59368 | Tumour | CRC | 2,732,325,034 | 99 | 283 | 15.0% | 3,649 |
| PROV195 | CF66218 | Normal | N/A | 782,201,552 | 35.6 | 417 | 7.9% | N/A |
| PROV195 | CF59957 | Tumour | CRC | 2,768,801,284 | 97.7 | 295 | 20.1% | 23,898 |
| PROV196 | CF66219 | Normal | N/A | 808,267,900 | 36.1 | 396 | 8.8% | N/A |
| PROV196 | CF59959 | Tumour | CRC | 2,800,565,908 | 89.9 | 276 | 24.3% | 4,706 |
| PROV197 | CF69766 | Normal | N/A | 964,751,418 | 39.8 | 475 | 16.4% | N/A |
| PROV197 | CF59951 | Tumour | CRC | 2,584,169,736 | 89.2 | 270 | 18.4% | 4,735 |
| PROV198 | CF69637 | Normal | N/A | 1,059,933,126 | 44.5 | 411 | 12.0% | N/A |
| PROV198 | CF59963 | Tumour | CRC | 2,566,265,664 | 83.9 | 260 | 18.3% | 4,324 |
| PROV199 | CF69707 | Normal | N/A | 1,069,894,568 | 44.6 | 502 | 15.7% | N/A |
| PROV199 | CF59949 | Tumour | CRC | 2,672,478,936 | 99.3 | 295 | 15.5% | 6,018 |
| PROV200 | CF69785 | Normal | N/A | 990,696,688 | 41.2 | 497 | 14.9% | N/A |
| PROV200 | CF67578 | Tumour | CRC | 2,523,672,872 | 88.1 | 277 | 17.4% | 3,714 |
| PROV201 | CF69657 | Normal | N/A | 944,996,132 | 41 | 451 | 12.3% | N/A |
| PROV201 | CF67584 | Tumour | CRC | 2,396,974,692 | 76.4 | 247 | 20.1% | 5,108 |
| PROV202 | CF69790 | Normal | N/A | 993,941,672 | 41.4 | 497 | 15.5% | N/A |
| PROV202 | CF67570 | Tumour | CRC | 2,380,505,914 | 68.3 | 212 | 21.5% | 842,124 |
| PROV203 | CF69803 | Normal | N/A | 1,287,687,496 | 56 | 536 | 11.4% | N/A |
| PROV203 | CF67572 | Tumour | CRC | 2,742,043,968 | 101.7 | 309 | 15.9% | 5,222 |
| PROV204 | CF69685 | Normal | N/A | 1,019,105,288 | 41.8 | 439 | 15.2% | N/A |
| PROV204 | CF67576 | Tumour | CRC | 2,849,813,258 | 113.7 | 341 | 13.4% | 25,789 |
| PROV205 | CF69823 | Normal | N/A | 966,937,570 | 42.6 | 535 | 11.8% | N/A |
| PROV205 | CF67582 | Tumour | CRC | 2,389,774,616 | 83.3 | 275 | 17.6% | 3,452 |
| PROV206 | CF69827 | Normal | N/A | 1,013,389,704 | 44.5 | 511 | 10.8% | N/A |
| PROV206 | CF67574 | Tumour | CRC | 2,746,937,052 | 102.5 | 295 | 15.7% | 7,007 |
| PROV207 | CF69642 | Normal | N/A | 929,256,294 | 40.3 | 428 | 11.5% | N/A |
| PROV207 | CF61953 | Tumour | CRC | 2,277,670,316 | 55.2 | 174 | 20.8% | 4,643 |
| PROV208 | CF69651 | Normal | N/A | 872,483,866 | 37.2 | 425 | 11.4% | N/A |
| PROV208 | CF66861 | Tumour | CRC | 2,817,962,558 | 96.6 | 258 | 15.6% | 30,942 |
| PROV209 | CF69795 | Normal | N/A | 939,326,538 | 38.3 | 468 | 15.6% | N/A |
| PROV209 | CF67568 | Tumour | CRC | 2,713,547,694 | 97.7 | 281 | 15.3% | 13,357 |

**Supplementary Table 5.** Summary of whole genome sequencing QC metrics for the PROVENC3 plasma cohort

| **Subject ID** | **Sample ID** | **Timepoint** | **Diagnosis** | **Days form surgery to sample collection** | **Total Reads** | **Total Reads Aligned** | **Median Insert Size (bp)** | **Mean Coverage (fold)** | **Fraction of Genome with ≥10-fold Depth** | **ctDNA Detected** | **Aggregate ctDNA VAF (%)** |
| --- | --- | --- | --- | --- | --- | --- | --- | --- | --- | --- | --- |
| PROV1 | CF65106 | Pre-Surgery | CRC | -22 | 1087409324 | 1085942651 | 169 | 30.234 | 0.932 | yes | 0.084 |
| PROV1 | CF72693 | Post-ACT-1 | CRC | 177 | 1212009196 | 1210708066 | 168 | 34.481 | 0.955 | no | NA |
| PROV1 | CF67863 | Post-Surgery | CRC | 16 | 1245242078 | 1243797634 | 166 | 33.670 | 0.952 | no | NA |
| PROV10 | CF72640 | Post-ACT-1 | CRC | 262 | 1195407232 | 1194187585 | 170 | 35.116 | 0.951 | no | NA |
| PROV10 | CF72433 | Post-Surgery | CRC | 28 | 1260230266 | 1258817352 | 169 | 37.100 | 0.955 | no | NA |
| PROV100 | CF72701 | Post-ACT-1 | CRC | 315 | 1530853328 | 1529087017 | 170 | 44.293 | 0.961 | yes | 0.006 |
| PROV100 | CF70137 | Post-Surgery | CRC | 3 | 834865252 | 833902511 | 165 | 22.350 | 0.875 | no | NA |
| PROV101 | CF72769 | Post-ACT-1 | CRC | 204 | 1404147320 | 1402238047 | 167 | 38.427 | 0.958 | no | NA |
| PROV101 | CF67898 | Post-Surgery | CRC | 13 | 1251056446 | 1249585088 | 168 | 34.992 | 0.950 | no | NA |
| PROV101 | CF65127 | Pre-Surgery | CRC | 0 | 1225864736 | 1224321748 | 168 | 33.963 | 0.949 | yes | 0.166 |
| PROV102 | CF72325 | Pre-Surgery | CRC | 0 | 1337785754 | 1336416108 | 170 | 38.835 | 0.953 | yes | 0.019 |
| PROV102 | CF72771 | Post-ACT-1 | CRC | 221 | 1272702196 | 1271403448 | 170 | 36.580 | 0.955 | no | NA |
| PROV102 | CF70398 | Post-Surgery | CRC | 3 | 927103188 | 926018926 | 165 | 24.926 | 0.924 | no | NA |
| PROV103 | CF72326 | Pre-Surgery | CRC | -5 | 1469224032 | 1467675806 | 167 | 40.793 | 0.954 | yes | 0.049 |
| PROV103 | CF70098 | Post-Surgery | CRC | 2 | 1183788556 | 1182540762 | 166 | 32.236 | 0.948 | no | NA |
| PROV103 | CF72742 | Post-ACT-1 | CRC | 199 | 1552345006 | 1550601464 | 168 | 43.366 | 0.956 | no | NA |
| PROV104 | CF69976 | Post-Surgery | CRC | 3 | 1507724342 | 1506071047 | 164 | 40.108 | 0.955 | no | NA |
| PROV104 | CF72703 | Post-ACT-1 | CRC | 147 | 1695928990 | 1694152694 | 165 | 45.657 | 0.957 | no | NA |
| PROV104 | CF72334 | Pre-Surgery | CRC | 0 | 1525482968 | 1523919912 | 168 | 42.467 | 0.952 | yes | 0.823 |
| PROV105 | CF69926 | Post-Surgery | CRC | 4 | 1392759524 | 1391250687 | 168 | 38.979 | 0.954 | no | NA |
| PROV105 | CF72778 | Post-ACT-1 | CRC | 207 | 1365004156 | 1363651874 | 174 | 40.295 | 0.956 | no | NA |
| PROV106 | CF69966 | Post-Surgery | CRC | 3 | 1338269190 | 1336412596 | 163 | 34.635 | 0.950 | no | NA |
| PROV106 | CF72337 | Pre-Surgery | CRC | 0 | 1307628190 | 1306144103 | 167 | 36.171 | 0.949 | yes | 0.106 |
| PROV106 | CF72739 | Post-ACT-1 | CRC | 181 | 1563142426 | 1561345331 | 165 | 41.837 | 0.953 | no | NA |
| PROV107 | CF69967 | Post-Surgery | CRC | 3 | 1357641002 | 1355886021 | 166 | 37.164 | 0.952 | no | NA |
| PROV107 | CF72338 | Pre-Surgery | CRC | 0 | 1235501992 | 1234091137 | 172 | 36.257 | 0.952 | yes | 0.142 |
| PROV108 | CF67899 | Post-Surgery | CRC | 4 | 1172662086 | 1171366672 | 165 | 31.567 | 0.932 | no | NA |
| PROV108 | CF66142 | Pre-Surgery | CRC | -13 | 1317252820 | 1315776544 | 168 | 37.112 | 0.955 | yes | 0.462 |
| PROV109 | CF72343 | Pre-Surgery | CRC | -1 | 1259373340 | 1258089390 | 172 | 37.050 | 0.941 | yes | 0.303 |
| PROV109 | CF70405 | Post-Surgery | CRC | 3 | 1254600048 | 1252991426 | 166 | 33.960 | 0.948 | no | NA |
| PROV109 | CF72654 | Post-ACT-1 | CRC | 599 | 1234453924 | 1233015898 | 170 | 36.435 | 0.953 | no | NA |
| PROV11 | CF72807 | Post-ACT-1 | CRC | 349 | 1371682960 | 1370207504 | 175 | 41.396 | 0.958 | no | NA |
| PROV11 | CF72431 | Pre-Surgery | CRC | -8 | 1343268518 | 1341300793 | 171 | 38.846 | 0.959 | yes | 0.108 |
| PROV11 | CF69954 | Post-Surgery | CRC | 24 | 1200783386 | 1199350941 | 169 | 34.000 | 0.920 | no | NA |
| PROV110 | CF72711 | Post-ACT-1 | CRC | 627 | 1297490252 | 1296070080 | 169 | 37.314 | 0.955 | no | NA |
| PROV110 | CF70113 | Post-Surgery | CRC | 3 | 1388573058 | 1387038573 | 165 | 37.202 | 0.936 | no | NA |
| PROV110 | CF72351 | Pre-Surgery | CRC | 0 | 1211685388 | 1210394995 | 170 | 34.784 | 0.939 | yes | 0.015 |
| PROV111 | CF72628 | Post-ACT-1 | CRC | 380 | 1301109264 | 1299803345 | 172 | 38.560 | 0.954 | no | NA |
| PROV111 | CF72401 | Pre-Surgery | CRC | 0 | 1342130334 | 1340789898 | 169 | 38.532 | 0.955 | yes | 0.110 |
| PROV111 | CF69937 | Post-Surgery | CRC | 3 | 1241429892 | 1240005585 | 165 | 33.653 | 0.908 | no | NA |
| PROV112 | CF69940 | Post-Surgery | CRC | 14 | 1305114830 | 1303546453 | 166 | 35.342 | 0.944 | no | NA |
| PROV112 | CF72597 | Post-ACT-1 | CRC | 141 | 1659942906 | 1657723214 | 167 | 45.962 | 0.962 | no | NA |
| PROV113 | CF65128 | Pre-Surgery | CRC | -1 | 1202163164 | 1200678249 | 169 | 34.316 | 0.954 | yes | 0.397 |
| PROV113 | CF72602 | Post-ACT-1 | CRC | 182 | 1422976590 | 1421408001 | 169 | 40.493 | 0.956 | no | NA |
| PROV113 | CF67900 | Post-Surgery | CRC | 14 | 1171505430 | 1170240117 | 167 | 32.068 | 0.947 | no | NA |
| PROV114 | CF72596 | Post-ACT-1 | CRC | 126 | 1241496426 | 1239967954 | 165 | 33.705 | 0.955 | no | NA |
| PROV114 | CF70397 | Post-Surgery | CRC | 14 | 1235782948 | 1234281316 | 168 | 34.642 | 0.946 | no | NA |
| PROV115 | CF65130 | Pre-Surgery | CRC | -1 | 1126923514 | 1125548069 | 169 | 32.113 | 0.947 | yes | 0.228 |
| PROV115 | CF72601 | Post-ACT-1 | CRC | 175 | 1398543968 | 1396965034 | 169 | 39.857 | 0.955 | no | NA |
| PROV115 | CF67901 | Post-Surgery | CRC | 17 | 1050709826 | 1049540574 | 165 | 28.284 | 0.932 | no | NA |
| PROV116 | CF72599 | Post-ACT-1 | CRC | 148 | 1084945324 | 1083572407 | 170 | 30.775 | 0.953 | no | NA |
| PROV116 | CF70139 | Post-Surgery | CRC | 15 | 1387386102 | 1385696834 | 164 | 36.710 | 0.945 | no | NA |
| PROV117 | CF70144 | Post-Surgery | CRC | 13 | 1258093524 | 1256374953 | 166 | 34.432 | 0.950 | yes | 0.025 |
| PROV118 | CF72698 | Post-ACT-1 | CRC | 179 | 1265895310 | 1264536359 | 171 | 36.775 | 0.958 | no | NA |
| PROV118 | CF70143 | Post-Surgery | CRC | 12 | 1328186858 | 1326621223 | 165 | 36.005 | 0.952 | no | NA |
| PROV119 | CF70146 | Post-Surgery | CRC | 11 | 1232640290 | 1231107818 | 164 | 32.860 | 0.940 | no | NA |
| PROV119 | CF72740 | Post-ACT-1 | CRC | 179 | 1212764600 | 1211454348 | 172 | 35.240 | 0.953 | no | NA |
| PROV12 | CF70071 | Post-Surgery | CRC | 18 | 1489348822 | 1487563892 | 169 | 42.000 | 0.960 | no | NA |
| PROV12 | CF72380 | Pre-Surgery | CRC | -10 | 1412820758 | 1411252294 | 169 | 40.258 | 0.956 | yes | 0.195 |
| PROV12 | CF72629 | Post-ACT-1 | CRC | 446 | 1218680314 | 1217421704 | 168 | 34.600 | 0.953 | no | NA |
| PROV120 | CF72741 | Post-ACT-1 | CRC | 166 | 1336596278 | 1335172736 | 168 | 37.447 | 0.957 | no | NA |
| PROV120 | CF70103 | Post-Surgery | CRC | 14 | 1233529504 | 1232058909 | 169 | 34.743 | 0.944 | no | NA |
| PROV121 | CF70100 | Post-Surgery | CRC | 11 | 1413196148 | 1411512217 | 166 | 38.543 | 0.951 | no | NA |
| PROV121 | CF72700 | Post-ACT-1 | CRC | 174 | 1129750608 | 1128611810 | 170 | 32.251 | 0.953 | no | NA |
| PROV122 | CF72436 | Post-Surgery | CRC | 11 | 1386833116 | 1385238828 | 164 | 37.350 | 0.955 | yes | 0.055 |
| PROV122 | CF72647 | Post-ACT-1 | CRC | 284 | 1232440576 | 1231134513 | 168 | 34.596 | 0.953 | yes | 0.347 |
| PROV123 | CF69975 | Post-Surgery | CRC | 14 | 1267060364 | 1265504883 | 166 | 34.657 | 0.953 | no | NA |
| PROV123 | CF72735 | Post-ACT-1 | CRC | 157 | 1400716168 | 1399161674 | 166 | 38.121 | 0.957 | no | NA |
| PROV124 | CF72644 | Post-ACT-1 | CRC | 204 | 1175274638 | 1173904201 | 170 | 34.394 | 0.955 | no | NA |
| PROV124 | CF69972 | Post-Surgery | CRC | 18 | 1464374892 | 1462497972 | 162 | 38.321 | 0.956 | no | NA |
| PROV125 | CF69968 | Post-Surgery | CRC | 11 | 1359604018 | 1358077120 | 159 | 35.315 | 0.948 | no | NA |
| PROV126 | CF69933 | Post-Surgery | CRC | 19 | 1196090518 | 1194699043 | 165 | 32.470 | 0.946 | no | NA |
| PROV126 | CF72777 | Post-ACT-1 | CRC | 264 | 1299794314 | 1298513600 | 168 | 36.409 | 0.955 | no | NA |
| PROV127 | CF66143 | Pre-Surgery | CRC | 0 | 1115865974 | 1114649980 | 171 | 32.209 | 0.953 | yes | 0.750 |
| PROV127 | CF72649 | Post-ACT-1 | CRC | 169 | 1170672784 | 1169495671 | 170 | 34.089 | 0.953 | yes | 5.177 |
| PROV127 | CF67902 | Post-Surgery | CRC | 7 | 1312181340 | 1310832428 | 165 | 35.127 | 0.947 | yes | 0.288 |
| PROV128 | CF66144 | Pre-Surgery | CRC | -1 | 1253517016 | 1251893742 | 171 | 36.376 | 0.957 | yes | 0.071 |
| PROV128 | CF82277 | Post-ACT-4 | CRC | 767 | 1139545670 | 1137994286 | 170 | 32.139 | 0.954 | yes | 0.148 |
| PROV128 | CF82271 | Post-ACT-2 | CRC | 418 | 1350611778 | 1348687482 | 169 | 39.765 | 0.960 | no | NA |
| PROV128 | CF82275 | Post-ACT-3 | CRC | 608 | 1421693686 | 1419835111 | 170 | 41.595 | 0.961 | yes | 0.029 |
| PROV128 | CF67903 | Post-Surgery | CRC | 11 | 1166820736 | 1165437585 | 169 | 32.836 | 0.951 | no | NA |
| PROV128 | CF72749 | Post-ACT-1 | CRC | 228 | 1128688750 | 1127428175 | 171 | 32.801 | 0.954 | yes | 0.015 |
| PROV129 | CF67904 | Post-Surgery | CRC | 18 | 1253662844 | 1252027810 | 164 | 32.901 | 0.797 | no | NA |
| PROV129 | CF66145 | Pre-Surgery | CRC | -4 | 1185798356 | 1184343675 | 170 | 33.976 | 0.955 | yes | 0.013 |
| PROV13 | CF72390 | Pre-Surgery | CRC | -9 | 1529855270 | 1528114260 | 168 | 43.617 | 0.950 | yes | 0.021 |
| PROV13 | CF72732 | Post-ACT-1 | CRC | 274 | 1156935578 | 1155698270 | 169 | 32.873 | 0.952 | no | NA |
| PROV13 | CF69955 | Post-Surgery | CRC | 29 | 1194392694 | 1192978949 | 170 | 34.570 | 0.911 | no | NA |
| PROV130 | CF69916 | Post-Surgery | CRC | 11 | 1553683582 | 1551951063 | 167 | 43.458 | 0.956 | no | NA |
| PROV130 | CF72364 | Pre-Surgery | CRC | -6 | 1250318668 | 1248921073 | 170 | 36.404 | 0.926 | yes | 0.086 |
| PROV130 | CF72725 | Post-ACT-1 | CRC | 169 | 1386689708 | 1385217151 | 166 | 38.568 | 0.954 | no | NA |
| PROV131 | CF72618 | Post-ACT-1 | CRC | 221 | 1283658872 | 1282057089 | 170 | 36.571 | 0.958 | no | NA |
| PROV131 | CF69911 | Post-Surgery | CRC | 19 | 1337841646 | 1336217292 | 163 | 35.355 | 0.935 | no | NA |
| PROV131 | CF72410 | Pre-Surgery | CRC | -1 | 1343034064 | 1341454680 | 168 | 37.825 | 0.954 | no | NA |
| PROV132 | CF72615 | Post-ACT-1 | CRC | 211 | 1155864720 | 1154427603 | 172 | 33.775 | 0.956 | no | NA |
| PROV132 | CF82876 | Post-ACT-2 | CRC | 336 | 1095940564 | 1094740662 | 170 | 31.125 | 0.948 | no | NA |
| PROV132 | CF69958 | Post-Surgery | CRC | 15 | 1090308324 | 1089007792 | 170 | 31.181 | 0.904 | no | NA |
| PROV132 | CF72387 | Pre-Surgery | CRC | -1 | 1197026818 | 1195634843 | 172 | 35.123 | 0.925 | no | NA |
| PROV133 | CF70072 | Post-Surgery | CRC | 13 | 1257031466 | 1255602873 | 168 | 35.620 | 0.954 | no | NA |
| PROV133 | CF72617 | Post-ACT-1 | CRC | 197 | 1009994490 | 1008830816 | 168 | 27.853 | 0.948 | no | NA |
| PROV133 | CF72393 | Pre-Surgery | CRC | 0 | 1314025800 | 1312619538 | 169 | 37.417 | 0.944 | yes | 0.723 |
| PROV134 | CF70093 | Post-Surgery | CRC | 25 | 1332482072 | 1330006466 | 169 | 37.542 | 0.957 | no | NA |
| PROV134 | CF72417 | Pre-Surgery | CRC | -7 | 1311693650 | 1310300581 | 171 | 37.536 | 0.949 | yes | 1.032 |
| PROV134 | CF72713 | Post-ACT-1 | CRC | 170 | 995690644 | 994619229 | 166 | 26.622 | 0.939 | no | NA |
| PROV135 | CF67905 | Post-Surgery | CRC | 11 | 1045347184 | 1042301197 | 165 | 28.195 | 0.927 | no | NA |
| PROV135 | CF66696 | Pre-Surgery | CRC | -24 | 1148629906 | 1147235522 | 168 | 32.813 | 0.950 | yes | 0.878 |
| PROV135 | CF72620 | Post-ACT-1 | CRC | 215 | 949548098 | 948547654 | 167 | 25.901 | 0.923 | no | NA |
| PROV136 | CF65131 | Pre-Surgery | CRC | 0 | 1321639988 | 1320004327 | 166 | 36.562 | 0.956 | yes | 0.012 |
| PROV136 | CF67906 | Post-Surgery | CRC | 3 | 1374819074 | 1373168876 | 164 | 36.559 | 0.955 | no | NA |
| PROV136 | CF72695 | Post-ACT-1 | CRC | 186 | 1330496070 | 1328944211 | 169 | 37.540 | 0.958 | no | NA |
| PROV137 | CF72369 | Pre-Surgery | CRC | -1 | 1427633572 | 1426144039 | 169 | 40.122 | 0.946 | yes | 0.028 |
| PROV137 | CF72688 | Post-ACT-1 | CRC | 211 | 880129422 | 879249111 | 170 | 24.769 | 0.931 | no | NA |
| PROV137 | CF69896 | Post-Surgery | CRC | 3 | 1344919974 | 1343413680 | 165 | 36.476 | 0.935 | no | NA |
| PROV138 | CF72671 | Post-ACT-1 | CRC | 207 | 1314507870 | 1313170945 | 171 | 37.757 | 0.955 | yes | 0.393 |
| PROV138 | CF65132 | Pre-Surgery | CRC | -13 | 1299401470 | 1297978972 | 171 | 37.672 | 0.955 | yes | 0.056 |
| PROV138 | CF67907 | Post-Surgery | CRC | 8 | 1391568318 | 1390103042 | 166 | 38.168 | 0.952 | no | NA |
| PROV139 | CF82263 | Post-ACT-3 | CRC | 574 | 896825950 | 895575461 | 168 | 24.625 | 0.940 | yes | 4.477 |
| PROV139 | CF72766 | Post-ACT-1 | CRC | 184 | 1394648588 | 1392934408 | 172 | 40.982 | 0.961 | no | NA |
| PROV139 | CF67908 | Post-Surgery | CRC | 4 | 1428230206 | 1426030228 | 166 | 39.171 | 0.955 | yes | 0.077 |
| PROV139 | CF82260 | Post-ACT-2 | CRC | 364 | 1070528238 | 1068848617 | 169 | 29.780 | 0.951 | yes | 0.108 |
| PROV139 | CF65133 | Pre-Surgery | CRC | 0 | 1246323094 | 1244675884 | 171 | 36.004 | 0.956 | yes | 0.171 |
| PROV14 | CF72391 | Pre-Surgery | CRC | -15 | 1359982594 | 1358553326 | 173 | 40.716 | 0.956 | yes | 0.012 |
| PROV14 | CF69941 | Post-Surgery | CRC | 13 | 1245911044 | 1244556528 | 166 | 34.456 | 0.933 | no | NA |
| PROV140 | CF67909 | Post-Surgery | CRC | 2 | 1351775356 | 1350323054 | 165 | 36.953 | 0.946 | no | NA |
| PROV140 | CF72697 | Post-ACT-1 | CRC | 203 | 948652698 | 947742487 | 168 | 26.185 | 0.946 | no | NA |
| PROV140 | CF82262 | Post-ACT-2 | CRC | 387 | 1056482196 | 1055368065 | 168 | 29.210 | 0.951 | no | NA |
| PROV140 | CF65134 | Pre-Surgery | CRC | -2 | 1425379488 | 1423688768 | 169 | 40.676 | 0.954 | yes | 0.006 |
| PROV141 | CF67910 | Post-Surgery | CRC | 12 | 1557758958 | 1556009137 | 166 | 42.668 | 0.955 | yes | 0.798 |
| PROV141 | CF65135 | Pre-Surgery | CRC | -1 | 1382560244 | 1380723650 | 169 | 39.211 | 0.955 | yes | 1.280 |
| PROV142 | CF67911 | Post-Surgery | CRC | 4 | 1086042584 | 1084794780 | 170 | 31.226 | 0.942 | no | NA |
| PROV142 | CF72604 | Post-ACT-1 | CRC | 174 | 790040544 | 789199791 | 172 | 22.590 | 0.937 | no | NA |
| PROV142 | CF65136 | Pre-Surgery | CRC | -7 | 1050095950 | 1048739415 | 167 | 28.913 | 0.946 | no | NA |
| PROV143 | CF72775 | Post-ACT-1 | CRC | 343 | 934425288 | 933222194 | 167 | 25.428 | 0.940 | no | NA |
| PROV143 | CF70399 | Post-Surgery | CRC | 4 | 1339215408 | 1338837608 | 150 | 32.913 | 0.949 | no | NA |
| PROV144 | CF72745 | Post-ACT-1 | CRC | 243 | 1405866466 | 1404325124 | 167 | 39.543 | 0.955 | no | NA |
| PROV144 | CF70104 | Post-Surgery | CRC | 4 | 1300451404 | 1299067056 | 165 | 35.020 | 0.948 | yes | 0.016 |
| PROV145 | CF72772 | Post-ACT-1 | CRC | 214 | 991386084 | 990381338 | 166 | 26.792 | 0.945 | no | NA |
| PROV145 | CF72332 | Pre-Surgery | CRC | 0 | 1509203390 | 1507612156 | 165 | 40.803 | 0.948 | yes | 1.251 |
| PROV145 | CF70105 | Post-Surgery | CRC | 4 | 1163247944 | 1162018139 | 164 | 31.457 | 0.940 | no | NA |
| PROV146 | CF70403 | Post-Surgery | CRC | 3 | 1421745356 | 1420064207 | 166 | 38.473 | 0.955 | no | NA |
| PROV147 | CF72652 | Post-ACT-1 | CRC | 148 | 997344768 | 996319957 | 164 | 25.973 | 0.938 | no | NA |
| PROV147 | CF67912 | Post-Surgery | CRC | 4 | 1028950014 | 1027825805 | 167 | 28.811 | 0.949 | no | NA |
| PROV147 | CF66146 | Pre-Surgery | CRC | 0 | 1116196264 | 1114891074 | 168 | 30.957 | 0.951 | yes | 0.047 |
| PROV148 | CF70107 | Post-Surgery | CRC | 19 | 1577330454 | 1575442521 | 163 | 41.505 | 0.948 | yes | 0.181 |
| PROV148 | CF72653 | Post-ACT-1 | CRC | 177 | 1420597970 | 1418970935 | 168 | 39.984 | 0.959 | yes | 0.404 |
| PROV148 | CF72371 | Pre-Surgery | CRC | -23 | 1521268028 | 1519271201 | 171 | 44.019 | 0.962 | yes | 0.247 |
| PROV149 | CF70108 | Post-Surgery | CRC | 3 | 1364245830 | 1362741725 | 166 | 37.307 | 0.945 | yes | 0.008 |
| PROV149 | CF72352 | Pre-Surgery | CRC | -7 | 1450887522 | 1449240725 | 169 | 41.780 | 0.938 | yes | 0.034 |
| PROV15 | CF72399 | Pre-Surgery | CRC | -6 | 1213973216 | 1212345460 | 167 | 33.139 | 0.950 | yes | 0.320 |
| PROV15 | CF69943 | Post-Surgery | CRC | 15 | 1265827300 | 1264327832 | 166 | 34.524 | 0.936 | no | NA |
| PROV15 | CF72660 | Post-ACT-1 | CRC | 195 | 1269550072 | 1268064119 | 167 | 34.746 | 0.951 | no | NA |
| PROV150 | CF70111 | Post-Surgery | CRC | 3 | 1352152780 | 1350573048 | 166 | 36.760 | 0.926 | yes | 0.013 |
| PROV150 | CF72751 | Post-ACT-1 | CRC | 185 | 1203425230 | 1202074384 | 170 | 34.878 | 0.940 | no | NA |
| PROV150 | CF72349 | Pre-Surgery | CRC | 0 | 1370986090 | 1369488245 | 169 | 39.947 | 0.956 | yes | 0.499 |
| PROV151 | CF72718 | Post-ACT-1 | CRC | 232 | 992726372 | 991565798 | 166 | 26.726 | 0.944 | no | NA |
| PROV151 | CF70120 | Post-Surgery | CRC | 3 | 1392655716 | 1390997056 | 165 | 37.759 | 0.955 | no | NA |
| PROV151 | CF72358 | Pre-Surgery | CRC | -1 | 1331812006 | 1330119403 | 168 | 37.285 | 0.916 | yes | 0.019 |
| PROV152 | CF72375 | Pre-Surgery | CRC | 0 | 1281398204 | 1280020885 | 171 | 36.975 | 0.955 | yes | 0.023 |
| PROV152 | CF69912 | Post-Surgery | CRC | 11 | 1298401712 | 1297002636 | 165 | 34.645 | 0.946 | no | NA |
| PROV152 | CF72793 | Post-ACT-1 | CRC | 189 | 909838458 | 908430743 | 166 | 24.885 | 0.939 | no | NA |
| PROV153 | CF69942 | Post-Surgery | CRC | 4 | 1340565110 | 1339172281 | 166 | 37.183 | 0.951 | no | NA |
| PROV153 | CF72402 | Pre-Surgery | CRC | 0 | 1369591574 | 1368180103 | 168 | 38.783 | 0.954 | yes | 0.027 |
| PROV153 | CF72659 | Post-ACT-1 | CRC | 182 | 845897660 | 845039020 | 167 | 23.182 | 0.939 | no | NA |
| PROV154 | CF66697 | Pre-Surgery | CRC | 0 | 1077990004 | 1076669196 | 168 | 30.089 | 0.950 | yes | 0.099 |
| PROV154 | CF67913 | Post-Surgery | CRC | 9 | 937126796 | 935972098 | 169 | 26.703 | 0.912 | no | NA |
| PROV154 | CF72731 | Post-ACT-1 | CRC | 219 | 1127409286 | 1126118334 | 169 | 31.625 | 0.952 | no | NA |
| PROV155 | CF72413 | Pre-Surgery | CRC | -1 | 1460115738 | 1458405078 | 171 | 42.585 | 0.959 | yes | 0.116 |
| PROV155 | CF70088 | Post-Surgery | CRC | 5 | 1375303920 | 1373606233 | 164 | 36.160 | 0.955 | yes | 0.094 |
| PROV156 | CF66698 | Pre-Surgery | CRC | -9 | 1219107828 | 1217625568 | 165 | 32.383 | 0.949 | yes | 0.024 |
| PROV156 | CF67914 | Post-Surgery | CRC | 4 | 1460779710 | 1459017712 | 166 | 39.876 | 0.933 | no | NA |
| PROV157 | CF66699 | Pre-Surgery | CRC | 0 | 1128665222 | 1127420283 | 166 | 30.833 | 0.947 | yes | 0.045 |
| PROV157 | CF67915 | Post-Surgery | CRC | 20 | 1375010064 | 1373498161 | 161 | 35.305 | 0.949 | no | NA |
| PROV158 | CF67916 | Post-Surgery | CRC | 6 | 1046918270 | 1045646409 | 166 | 28.574 | 0.936 | no | NA |
| PROV158 | CF72763 | Post-ACT-1 | CRC | 195 | 1185575674 | 1184218975 | 169 | 33.104 | 0.941 | no | NA |
| PROV159 | CF66148 | Pre-Surgery | CRC | -10 | 1105602038 | 1104137362 | 169 | 30.443 | 0.950 | yes | 0.277 |
| PROV159 | CF72783 | Post-ACT-1 | CRC | 184 | 1303560178 | 1301959585 | 167 | 35.552 | 0.956 | no | NA |
| PROV159 | CF67918 | Post-Surgery | CRC | 16 | 1060275492 | 1058964863 | 168 | 29.795 | 0.948 | no | NA |
| PROV16 | CF72407 | Pre-Surgery | CRC | -32 | 1327688266 | 1326178735 | 170 | 38.396 | 0.955 | yes | 0.035 |
| PROV16 | CF70085 | Post-Surgery | CRC | 18 | 1015331112 | 1014061254 | 171 | 29.197 | 0.947 | no | NA |
| PROV16 | CF72624 | Post-ACT-1 | CRC | 274 | 1100554504 | 1099370931 | 171 | 31.714 | 0.951 | no | NA |
| PROV160 | CF67919 | Post-Surgery | CRC | 20 | 1247474534 | 1245930566 | 163 | 33.054 | 0.930 | no | NA |
| PROV160 | CF72785 | Post-ACT-1 | CRC | 170 | 1195141506 | 1193703121 | 165 | 31.932 | 0.951 | no | NA |
| PROV160 | CF66149 | Pre-Surgery | CRC | -14 | 1144176890 | 1142782592 | 164 | 30.219 | 0.949 | yes | 0.008 |
| PROV161 | CF67920 | Post-Surgery | CRC | 13 | 1257290990 | 1255728715 | 169 | 35.425 | 0.950 | yes | 0.255 |
| PROV161 | CF66150 | Pre-Surgery | CRC | -48 | 1279951346 | 1278362850 | 169 | 35.818 | 0.956 | yes | 0.626 |
| PROV162 | CF72350 | Pre-Surgery | CRC | -21 | 1332097522 | 1330482240 | 171 | 38.315 | 0.936 | yes | 0.036 |
| PROV162 | CF72790 | Post-ACT-1 | CRC | 236 | 1157133816 | 1155815489 | 169 | 32.310 | 0.952 | no | NA |
| PROV162 | CF70109 | Post-Surgery | CRC | 14 | 1321378490 | 1319773994 | 169 | 38.000 | 0.950 | no | NA |
| PROV163 | CF72354 | Pre-Surgery | CRC | -6 | 1351272662 | 1349896001 | 169 | 37.978 | 0.950 | yes | 0.028 |
| PROV163 | CF72788 | Post-ACT-1 | CRC | 182 | 1209534396 | 1208382323 | 171 | 34.467 | 0.953 | no | NA |
| PROV163 | CF70112 | Post-Surgery | CRC | 8 | 1381823276 | 1380200671 | 167 | 38.246 | 0.954 | no | NA |
| PROV164 | CF69918 | Post-Surgery | CRC | 17 | 1247099020 | 1245642861 | 171 | 36.175 | 0.953 | no | NA |
| PROV164 | CF72759 | Post-ACT-1 | CRC | 182 | 1066082148 | 1064891006 | 171 | 30.692 | 0.950 | no | NA |
| PROV164 | CF72363 | Pre-Surgery | CRC | -27 | 1290324474 | 1288842137 | 172 | 37.952 | 0.950 | yes | 0.009 |
| PROV165 | CF69922 | Post-Surgery | CRC | 27 | 1321308402 | 1319762387 | 168 | 36.713 | 0.953 | no | NA |
| PROV165 | CF72795 | Post-ACT-1 | CRC | 187 | 1229960904 | 1228641482 | 168 | 33.860 | 0.943 | no | NA |
| PROV165 | CF72377 | Pre-Surgery | CRC | 0 | 1413559102 | 1411650406 | 169 | 39.910 | 0.956 | yes | 0.631 |
| PROV166 | CF72612 | Post-ACT-1 | CRC | 167 | 1106907994 | 1105739918 | 169 | 31.040 | 0.951 | no | NA |
| PROV166 | CF69900 | Post-Surgery | CRC | 12 | 1074874894 | 1073559201 | 172 | 31.575 | 0.941 | no | NA |
| PROV166 | CF72381 | Pre-Surgery | CRC | -35 | 1438162354 | 1436569251 | 170 | 41.383 | 0.957 | yes | 0.040 |
| PROV167 | CF69909 | Post-Surgery | CRC | 18 | 1391683136 | 1390181544 | 170 | 39.966 | 0.955 | no | NA |
| PROV167 | CF72405 | Pre-Surgery | CRC | -10 | 1388346178 | 1386949907 | 168 | 40.275 | 0.952 | yes | 14.734 |
| PROV168 | CF69935 | Post-Surgery | CRC | 14 | 1354764816 | 1353182219 | 171 | 39.606 | 0.946 | no | NA |
| PROV168 | CF72803 | Post-ACT-1 | CRC | 341 | 920722630 | 919799165 | 170 | 26.305 | 0.941 | no | NA |
| PROV169 | CF72804 | Post-ACT-1 | CRC | 343 | 995084308 | 993390780 | 169 | 27.709 | 0.937 | no | NA |
| PROV169 | CF69950 | Post-Surgery | CRC | 15 | 1274048472 | 1272365554 | 169 | 36.036 | 0.843 | no | NA |
| PROV169 | CF72425 | Pre-Surgery | CRC | -6 | 1612629438 | 1610019647 | 171 | 47.052 | 0.962 | yes | 0.028 |
| PROV17 | CF70413 | Post-Surgery | CRC | 20 | 1049948440 | 1048526943 | 168 | 29.000 | 0.940 | no | NA |
| PROV17 | CF72409 | Pre-Surgery | CRC | -8 | 1477501930 | 1475826934 | 169 | 42.223 | 0.957 | yes | 0.182 |
| PROV17 | CF72734 | Post-ACT-1 | CRC | 202 | 1350204778 | 1348778437 | 167 | 36.935 | 0.956 | yes | 0.007 |
| PROV17 | CF82877 | Post-ACT-2 | CRC | 349 | 1427859390 | 1426234702 | 168 | 40.616 | 0.957 | yes | 0.255 |
| PROV170 | CF72429 | Pre-Surgery | CRC | -25 | 1319246332 | 1317572936 | 167 | 37.173 | 0.958 | yes | 0.123 |
| PROV170 | CF70418 | Post-Surgery | CRC | 36 | 1232001620 | 1230325570 | 168 | 34.500 | 0.938 | no | NA |
| PROV170 | CF72630 | Post-ACT-1 | CRC | 205 | 1148277050 | 1147024582 | 167 | 31.350 | 0.938 | no | NA |
| PROV171 | CF66701 | Pre-Surgery | CRC | -7 | 1430134584 | 1428560829 | 168 | 40.111 | 0.954 | no | NA |
| PROV171 | CF72809 | Post-ACT-1 | CRC | 343 | 1312347654 | 1310958790 | 169 | 37.064 | 0.953 | no | NA |
| PROV171 | CF67921 | Post-Surgery | CRC | 21 | 1705522350 | 1703430265 | 168 | 48.140 | 0.952 | no | NA |
| PROV172 | CF72411 | Pre-Surgery | CRC | -3 | 1126779224 | 1125372931 | 173 | 33.577 | 0.937 | yes | 0.071 |
| PROV172 | CF70118 | Post-Surgery | CRC | 28 | 1284989154 | 1283077889 | 171 | 37.127 | 0.901 | no | NA |
| PROV173 | CF69949 | Post-Surgery | CRC | 49 | 1194691448 | 1193183974 | 170 | 34.245 | 0.801 | no | NA |
| PROV173 | CF72806 | Post-ACT-1 | CRC | 366 | 1165032304 | 1163821916 | 169 | 32.680 | 0.944 | no | NA |
| PROV174 | CF69963 | Post-Surgery | CRC | 12 | 1452608848 | 1450852617 | 168 | 41.407 | 0.922 | no | NA |
| PROV174 | CF72356 | Pre-Surgery | CRC | 0 | 1238091588 | 1236658307 | 170 | 35.950 | 0.949 | yes | 0.010 |
| PROV174 | CF72609 | Post-ACT-1 | CRC | 179 | 1264506386 | 1262858939 | 168 | 34.802 | 0.957 | no | NA |
| PROV175 | CF70127 | Post-Surgery | CRC | 8 | 1384990812 | 1383317610 | 165 | 37.457 | 0.955 | no | NA |
| PROV175 | CF72684 | Post-ACT-1 | CRC | 162 | 1169259098 | 1167941272 | 168 | 32.609 | 0.933 | no | NA |
| PROV176 | CF66702 | Pre-Surgery | CRC | 0 | 1102914006 | 1101630541 | 169 | 30.679 | 0.949 | yes | 0.042 |
| PROV176 | CF67922 | Post-Surgery | CRC | 11 | 1045180906 | 1043374404 | 170 | 29.834 | 0.917 | yes | 0.049 |
| PROV177 | CF70123 | Post-Surgery | CRC | 8 | 1649681340 | 1647872452 | 168 | 46.861 | 0.956 | no | NA |
| PROV178 | CF72687 | Post-ACT-1 | CRC | 154 | 1152856966 | 1151668015 | 169 | 32.269 | 0.938 | no | NA |
| PROV178 | CF70128 | Post-Surgery | CRC | 23 | 1671367836 | 1669460677 | 170 | 47.826 | 0.957 | no | NA |
| PROV179 | CF72355 | Pre-Surgery | CRC | -20 | 1229495516 | 1228341540 | 178 | 38.163 | 0.955 | yes | 0.011 |
| PROV179 | CF70125 | Post-Surgery | CRC | 65 | 1468537178 | 1466711795 | 172 | 43.221 | 0.961 | yes | 3.134 |
| PROV18 | CF72799 | Post-ACT-1 | CRC | 228 | 1067057920 | 1065891039 | 172 | 31.090 | 0.937 | no | NA |
| PROV18 | CF66685 | Pre-Surgery | CRC | -22 | 1171306662 | 1169847869 | 170 | 32.995 | 0.952 | yes | 0.020 |
| PROV18 | CF67869 | Post-Surgery | CRC | 20 | 1202920556 | 1201363100 | 168 | 33.050 | 0.950 | no | NA |
| PROV180 | CF70126 | Post-Surgery | CRC | 17 | 844159760 | 843194187 | 170 | 24.771 | 0.905 | no | NA |
| PROV180 | CF72724 | Post-ACT-1 | CRC | 193 | 898279034 | 897232435 | 172 | 26.730 | 0.943 | yes | 0.009 |
| PROV181 | CF69915 | Post-Surgery | CRC | 30 | 1135583102 | 1134069348 | 169 | 32.620 | 0.935 | no | NA |
| PROV181 | CF72361 | Pre-Surgery | CRC | -7 | 1367186230 | 1365588872 | 169 | 38.957 | 0.949 | yes | 0.019 |
| PROV181 | CF72726 | Post-ACT-1 | CRC | 198 | 1132748532 | 1131464383 | 172 | 32.587 | 0.954 | no | NA |
| PROV182 | CF72666 | Post-ACT-1 | CRC | 336 | 948513480 | 947529840 | 171 | 27.282 | 0.947 | no | NA |
| PROV182 | CF72389 | Pre-Surgery | CRC | -22 | 1324145300 | 1322734500 | 171 | 38.785 | 0.955 | yes | 0.019 |
| PROV182 | CF69947 | Post-Surgery | CRC | 14 | 1390873474 | 1388890799 | 167 | 38.512 | 0.951 | no | NA |
| PROV183 | CF72396 | Pre-Surgery | CRC | -20 | 1372359558 | 1370844291 | 168 | 38.872 | 0.955 | yes | 0.142 |
| PROV183 | CF69903 | Post-Surgery | CRC | 10 | 1501919066 | 1500224337 | 166 | 41.312 | 0.946 | no | NA |
| PROV184 | CF72398 | Pre-Surgery | CRC | -29 | 1426090192 | 1424598843 | 172 | 42.540 | 0.956 | yes | 0.039 |
| PROV184 | CF70131 | Post-Surgery | CRC | 29 | 1399100490 | 1397405153 | 169 | 39.582 | 0.955 | yes | 0.097 |
| PROV185 | CF72658 | Post-ACT-1 | CRC | 169 | 1083730986 | 1082381736 | 169 | 30.334 | 0.949 | no | NA |
| PROV185 | CF69907 | Post-Surgery | CRC | 14 | 1301483794 | 1299782910 | 166 | 36.000 | 0.930 | no | NA |
| PROV185 | CF72400 | Pre-Surgery | CRC | -20 | 1409501808 | 1407882043 | 169 | 40.720 | 0.958 | yes | 0.053 |
| PROV186 | CF70414 | Post-Surgery | CRC | 21 | 1419291450 | 1417535609 | 164 | 37.756 | 0.944 | no | NA |
| PROV186 | CF72412 | Pre-Surgery | CRC | -22 | 1450159180 | 1448138173 | 166 | 40.229 | 0.956 | yes | 0.151 |
| PROV186 | CF72636 | Post-ACT-1 | CRC | 201 | 1035552250 | 1034237905 | 166 | 28.231 | 0.941 | no | NA |
| PROV187 | CF65137 | Pre-Surgery | CRC | 0 | 920014300 | 918670315 | 171 | 25.911 | 0.938 | yes | 0.022 |
| PROV187 | CF67923 | Post-Surgery | CRC | 15 | 1054640730 | 1053325277 | 173 | 31.076 | 0.949 | no | NA |
| PROV187 | CF72668 | Post-ACT-1 | CRC | 176 | 984042902 | 982849421 | 172 | 28.415 | 0.948 | no | NA |
| PROV188 | CF72674 | Post-ACT-1 | CRC | 209 | 1362197946 | 1360552254 | 167 | 37.790 | 0.957 | no | NA |
| PROV188 | CF67925 | Post-Surgery | CRC | 36 | 1206964788 | 1205502664 | 168 | 33.772 | 0.951 | no | NA |
| PROV188 | CF65139 | Pre-Surgery | CRC | -67 | 1117923052 | 1116559635 | 170 | 31.250 | 0.951 | yes | 0.029 |
| PROV189 | CF72767 | Post-ACT-1 | CRC | 214 | 1108065514 | 1106725270 | 173 | 32.023 | 0.954 | yes | 0.004 |
| PROV189 | CF65140 | Pre-Surgery | CRC | 0 | 1110738566 | 1109305529 | 169 | 30.568 | 0.949 | yes | 0.076 |
| PROV189 | CF67926 | Post-Surgery | CRC | 36 | 812508948 | 811328905 | 171 | 22.944 | 0.915 | no | NA |
| PROV19 | CF72626 | Post-ACT-1 | CRC | 240 | 1275373952 | 1274083118 | 173 | 37.474 | 0.955 | no | NA |
| PROV19 | CF70417 | Post-Surgery | CRC | 16 | 1204583922 | 1203207072 | 169 | 33.888 | 0.927 | no | NA |
| PROV19 | CF72421 | Pre-Surgery | CRC | -12 | 1260413042 | 1259094143 | 168 | 36.477 | 0.953 | no | NA |
| PROV190 | CF72670 | Post-ACT-1 | CRC | 175 | 1031911558 | 1030849404 | 169 | 29.053 | 0.950 | no | NA |
| PROV190 | CF69897 | Post-Surgery | CRC | 20 | 1296842266 | 1295430370 | 169 | 36.768 | 0.954 | no | NA |
| PROV191 | CF72673 | Post-ACT-1 | CRC | 186 | 1239822540 | 1238546000 | 168 | 34.465 | 0.954 | no | NA |
| PROV191 | CF67927 | Post-Surgery | CRC | 15 | 1060917290 | 1059575338 | 167 | 29.297 | 0.929 | no | NA |
| PROV191 | CF65141 | Pre-Surgery | CRC | -45 | 1067662206 | 1066495083 | 167 | 29.172 | 0.943 | yes | 0.092 |
| PROV192 | CF65142 | Pre-Surgery | CRC | -14 | 1052433806 | 1051255104 | 168 | 29.227 | 0.944 | no | NA |
| PROV192 | CF67928 | Post-Surgery | CRC | 15 | 1244485892 | 1243105685 | 168 | 34.811 | 0.922 | no | NA |
| PROV192 | CF72600 | Post-ACT-1 | CRC | 222 | 1185068984 | 1183768174 | 168 | 33.329 | 0.953 | no | NA |
| PROV193 | CF72747 | Post-ACT-1 | CRC | 356 | 1003538376 | 1002328319 | 172 | 29.027 | 0.947 | no | NA |
| PROV193 | CF69934 | Post-Surgery | CRC | 15 | 1365687400 | 1363913834 | 169 | 39.000 | 0.950 | no | NA |
| PROV194 | CF72646 | Post-ACT-1 | CRC | 393 | 1063232862 | 1061981513 | 168 | 29.576 | 0.945 | no | NA |
| PROV194 | CF70145 | Post-Surgery | CRC | 32 | 1391615282 | 1389881303 | 170 | 40.000 | 0.960 | no | NA |
| PROV195 | CF65145 | Pre-Surgery | CRC | -5 | 1047350050 | 1045985942 | 167 | 29.072 | 0.943 | yes | 0.007 |
| PROV195 | CF72598 | Post-ACT-1 | CRC | 168 | 1291881978 | 1290159995 | 171 | 37.874 | 0.959 | no | NA |
| PROV195 | CF67929 | Post-Surgery | CRC | 37 | 1197379758 | 1195933196 | 168 | 33.884 | 0.947 | no | NA |
| PROV196 | CF67930 | Post-Surgery | CRC | 4 | 1266365664 | 1264910343 | 165 | 33.922 | 0.949 | yes | 0.010 |
| PROV196 | CF65146 | Pre-Surgery | CRC | -49 | 1025746798 | 1024551054 | 170 | 28.814 | 0.945 | yes | 0.206 |
| PROV196 | CF72603 | Post-ACT-1 | CRC | 141 | 906278472 | 905155229 | 171 | 26.377 | 0.943 | yes | 0.076 |
| PROV197 | CF70095 | Post-Surgery | CRC | 29 | 1244647390 | 1243222410 | 169 | 35.457 | 0.949 | no | NA |
| PROV197 | CF72744 | Post-ACT-1 | CRC | 204 | 1353117332 | 1351616301 | 170 | 39.125 | 0.958 | no | NA |
| PROV198 | CF72709 | Post-ACT-1 | CRC | 210 | 1539870398 | 1538150007 | 168 | 43.098 | 0.957 | no | NA |
| PROV198 | CF70074 | Post-Surgery | CRC | 14 | 1345333210 | 1343821216 | 171 | 38.670 | 0.956 | no | NA |
| PROV199 | CF72774 | Post-ACT-1 | CRC | 191 | 1364847256 | 1363265816 | 170 | 39.372 | 0.960 | no | NA |
| PROV199 | CF69924 | Post-Surgery | CRC | 9 | 1258093906 | 1256310510 | 166 | 34.383 | 0.943 | no | NA |
| PROV2 | CF67864 | Post-Surgery | CRC | 15 | 1062938834 | 1061775482 | 170 | 29.813 | 0.948 | no | NA |
| PROV2 | CF65107 | Pre-Surgery | CRC | -5 | 1164677456 | 1163138994 | 170 | 33.209 | 0.951 | yes | 0.018 |
| PROV2 | CF72692 | Post-ACT-1 | CRC | 149 | 1266005578 | 1264653141 | 170 | 35.867 | 0.954 | no | NA |
| PROV20 | CF67870 | Post-Surgery | CRC | 17 | 1130753564 | 1129481619 | 167 | 30.975 | 0.945 | no | NA |
| PROV20 | CF72810 | Post-ACT-1 | CRC | 424 | 1300463414 | 1299138092 | 172 | 38.141 | 0.952 | no | NA |
| PROV20 | CF66686 | Pre-Surgery | CRC | -18 | 1159506278 | 1158139738 | 169 | 32.871 | 0.952 | yes | 0.051 |
| PROV200 | CF72683 | Post-ACT-1 | CRC | 236 | 1301108990 | 1299684658 | 174 | 38.968 | 0.956 | no | NA |
| PROV200 | CF70119 | Post-Surgery | CRC | 71 | 1199994652 | 1198665501 | 173 | 35.678 | 0.954 | no | NA |
| PROV201 | CF72685 | Post-ACT-1 | CRC | 223 | 1131886874 | 1130567109 | 172 | 33.431 | 0.936 | no | NA |
| PROV201 | CF70148 | Post-Surgery | CRC | 42 | 1291605774 | 1289960009 | 169 | 36.175 | 0.955 | no | NA |
| PROV202 | CF70409 | Post-Surgery | CRC | 5 | 1408017160 | 1406334066 | 165 | 37.769 | 0.943 | no | NA |
| PROV202 | CF72761 | Post-ACT-1 | CRC | 224 | 1341092188 | 1339519835 | 169 | 38.260 | 0.955 | no | NA |
| PROV203 | CF72613 | Post-ACT-1 | CRC | 192 | 1374472940 | 1372827916 | 164 | 36.663 | 0.955 | no | NA |
| PROV203 | CF72385 | Pre-Surgery | CRC | -1 | 1418454320 | 1416858687 | 167 | 40.430 | 0.954 | yes | 0.018 |
| PROV203 | CF69960 | Post-Surgery | CRC | 17 | 1328771420 | 1327089342 | 160 | 34.880 | 0.927 | no | NA |
| PROV204 | CF72408 | Pre-Surgery | CRC | -1 | 1436044562 | 1434406725 | 169 | 41.357 | 0.956 | yes | 1.370 |
| PROV204 | CF72632 | Post-ACT-1 | CRC | 377 | 1283910576 | 1282520815 | 172 | 37.984 | 0.954 | no | NA |
| PROV204 | CF70412 | Post-Surgery | CRC | 29 | 1559860766 | 1554485876 | 168 | 43.716 | 0.956 | no | NA |
| PROV205 | CF70090 | Post-Surgery | CRC | 40 | 1435753024 | 1433976854 | 169 | 40.756 | 0.959 | no | NA |
| PROV205 | CF72661 | Post-ACT-1 | CRC | 182 | 1334807766 | 1333033491 | 165 | 36.433 | 0.954 | yes | 0.010 |
| PROV205 | CF82878 | Post-ACT-2 | CRC | 374 | 1102907980 | 1101609437 | 170 | 31.380 | 0.951 | yes | 1.322 |
| PROV206 | CF72808 | Post-ACT-1 | CRC | 484 | 1183819738 | 1182315385 | 174 | 35.458 | 0.950 | no | NA |
| PROV206 | CF70087 | Post-Surgery | CRC | 21 | 1342886136 | 1341162257 | 168 | 38.267 | 0.955 | no | NA |
| PROV207 | CF70401 | Post-Surgery | CRC | 48 | 1259844690 | 1258383300 | 165 | 33.621 | 0.938 | no | NA |
| PROV207 | CF72677 | Post-ACT-1 | CRC | 252 | 1241575878 | 1239911956 | 170 | 34.737 | 0.955 | no | NA |
| PROV208 | CF70110 | Post-Surgery | CRC | 3 | 1512893808 | 1511250001 | 168 | 41.886 | 0.953 | no | NA |
| PROV208 | CF72787 | Post-ACT-1 | CRC | 196 | 1252249446 | 1250982531 | 172 | 36.563 | 0.955 | yes | 0.008 |
| PROV209 | CF72794 | Post-ACT-1 | CRC | 186 | 1283792704 | 1282216343 | 168 | 36.777 | 0.954 | no | NA |
| PROV209 | CF72374 | Pre-Surgery | CRC | -3 | 1468882758 | 1467329843 | 170 | 42.797 | 0.957 | yes | 0.100 |
| PROV209 | CF69920 | Post-Surgery | CRC | 3 | 1443184144 | 1441584793 | 165 | 39.705 | 0.953 | yes | 0.007 |
| PROV21 | CF72357 | Pre-Surgery | CRC | -8 | 1140261186 | 1138361859 | 171 | 33.178 | 0.951 | yes | 0.233 |
| PROV21 | CF72717 | Post-ACT-1 | CRC | 172 | 1365569280 | 1364170067 | 168 | 38.510 | 0.955 | no | NA |
| PROV21 | CF70121 | Post-Surgery | CRC | 13 | 1300095996 | 1298491657 | 162 | 34.519 | 0.950 | no | NA |
| PROV22 | CF72801 | Post-ACT-1 | CRC | 370 | 1176757286 | 1175499737 | 167 | 32.479 | 0.951 | no | NA |
| PROV22 | CF67871 | Post-Surgery | CRC | 14 | 1259293954 | 1257886196 | 163 | 32.950 | 0.940 | no | NA |
| PROV22 | CF66687 | Pre-Surgery | CRC | -12 | 1276818800 | 1275441352 | 164 | 34.330 | 0.951 | yes | 0.020 |
| PROV23 | CF69953 | Post-Surgery | CRC | 13 | 1323711538 | 1322269716 | 166 | 36.132 | 0.949 | no | NA |
| PROV23 | CF72665 | Post-ACT-1 | CRC | 158 | 1269087564 | 1267407337 | 168 | 35.734 | 0.953 | no | NA |
| PROV23 | CF72422 | Pre-Surgery | CRC | -48 | 1428597654 | 1427073836 | 170 | 41.981 | 0.956 | yes | 0.011 |
| PROV24 | CF72426 | Pre-Surgery | CRC | -4 | 1469856820 | 1468192019 | 168 | 41.981 | 0.959 | yes | 0.048 |
| PROV24 | CF72621 | Post-ACT-1 | CRC | 203 | 1120084194 | 1118883197 | 170 | 32.072 | 0.949 | no | NA |
| PROV24 | CF69948 | Post-Surgery | CRC | 11 | 958236390 | 957006573 | 167 | 26.511 | 0.766 | no | NA |
| PROV25 | CF72427 | Pre-Surgery | CRC | -9 | 1248268030 | 1246829460 | 170 | 35.911 | 0.957 | no | NA |
| PROV25 | CF70134 | Post-Surgery | CRC | 9 | 1389372776 | 1387805586 | 168 | 38.500 | 0.956 | no | NA |
| PROV25 | CF72623 | Post-ACT-1 | CRC | 183 | 1313513910 | 1312087575 | 168 | 36.253 | 0.956 | no | NA |
| PROV26 | CF72432 | Pre-Surgery | CRC | 0 | 1112433808 | 1110976924 | 168 | 32.153 | 0.952 | yes | 0.198 |
| PROV26 | CF70419 | Post-Surgery | CRC | 15 | 1088386310 | 1087015269 | 168 | 30.000 | 0.940 | no | NA |
| PROV27 | CF72802 | Post-ACT-1 | CRC | 568 | 1219839246 | 1218423339 | 170 | 34.775 | 0.953 | no | NA |
| PROV27 | CF69919 | Post-Surgery | CRC | 4 | 1359246982 | 1357626885 | 165 | 37.000 | 0.960 | no | NA |
| PROV27 | CF72376 | Pre-Surgery | CRC | -2 | 1356961724 | 1355025954 | 173 | 39.884 | 0.961 | yes | 0.310 |
| PROV28 | CF72378 | Pre-Surgery | CRC | -2 | 1248569714 | 1246960821 | 172 | 37.134 | 0.959 | yes | 0.104 |
| PROV28 | CF69921 | Post-Surgery | CRC | 7 | 1348820396 | 1347231710 | 166 | 36.694 | 0.950 | no | NA |
| PROV28 | CF72760 | Post-ACT-1 | CRC | 187 | 1225966972 | 1224629288 | 169 | 34.553 | 0.952 | yes | 0.015 |
| PROV29 | CF69964 | Post-Surgery | CRC | 5 | 1267554274 | 1266168156 | 163 | 34.013 | 0.918 | no | NA |
| PROV29 | CF72797 | Post-ACT-1 | CRC | 176 | 1222911404 | 1221680982 | 166 | 33.478 | 0.950 | no | NA |
| PROV29 | CF72382 | Pre-Surgery | CRC | -2 | 1584036812 | 1581716248 | 170 | 45.907 | 0.958 | yes | 0.077 |
| PROV3 | CF65108 | Pre-Surgery | CRC | -3 | 1216974038 | 1215498052 | 169 | 34.110 | 0.952 | yes | 0.083 |
| PROV3 | CF72694 | Post-ACT-1 | CRC | 101 | 1425330278 | 1423900233 | 167 | 39.790 | 0.955 | yes | 0.008 |
| PROV3 | CF67865 | Post-Surgery | CRC | 15 | 1125767240 | 1124486276 | 167 | 31.502 | 0.932 | no | NA |
| PROV30 | CF70150 | Post-Surgery | CRC | 11 | 1090978064 | 1089808382 | 167 | 30.000 | 0.940 | no | NA |
| PROV30 | CF72416 | Pre-Surgery | CRC | -2 | 1388939806 | 1387491376 | 171 | 41.262 | 0.954 | yes | 0.269 |
| PROV30 | CF72733 | Post-ACT-1 | CRC | 182 | 1168929048 | 1167788100 | 173 | 33.878 | 0.952 | no | NA |
| PROV31 | CF65111 | Pre-Surgery | CRC | 0 | 1192937934 | 1191456089 | 168 | 32.744 | 0.951 | yes | 0.034 |
| PROV31 | CF67872 | Post-Surgery | CRC | 9 | 1352994588 | 1351441772 | 164 | 35.495 | 0.954 | no | NA |
| PROV31 | CF72764 | Post-ACT-1 | CRC | 173 | 1399228806 | 1397679537 | 166 | 37.585 | 0.957 | no | NA |
| PROV32 | CF69895 | Post-Surgery | CRC | 13 | 1525617720 | 1523868300 | 167 | 42.482 | 0.954 | no | NA |
| PROV33 | CF70133 | Post-Surgery | CRC | 16 | 1611383086 | 1609552083 | 169 | 45.571 | 0.957 | no | NA |
| PROV33 | CF72748 | Post-ACT-1 | CRC | 364 | 1025706060 | 1024683757 | 168 | 28.779 | 0.941 | yes | 0.011 |
| PROV34 | CF72324 | Pre-Surgery | CRC | 0 | 1331699458 | 1330167449 | 168 | 37.750 | 0.948 | no | NA |
| PROV34 | CF72743 | Post-ACT-1 | CRC | 245 | 1211921082 | 1210606284 | 168 | 33.388 | 0.952 | yes | 0.009 |
| PROV34 | CF69939 | Post-Surgery | CRC | 4 | 1792745690 | 1790759700 | 168 | 49.576 | 0.957 | no | NA |
| PROV35 | CF70097 | Post-Surgery | CRC | 27 | 1664132530 | 1662109441 | 166 | 45.659 | 0.956 | yes | 0.009 |
| PROV36 | CF72633 | Post-ACT-1 | CRC | 364 | 1115561182 | 1114329149 | 169 | 31.523 | 0.951 | yes | 0.006 |
| PROV36 | CF70086 | Post-Surgery | CRC | 16 | 1510336622 | 1508516930 | 166 | 42.000 | 0.960 | no | NA |
| PROV36 | CF72415 | Pre-Surgery | CRC | 0 | 1131637154 | 1130305169 | 169 | 32.567 | 0.907 | no | NA |
| PROV37 | CF70116 | Post-Surgery | CRC | 16 | 1455053582 | 1453299923 | 164 | 38.643 | 0.928 | no | NA |
| PROV37 | CF72800 | Post-ACT-1 | CRC | 358 | 1111501290 | 1110279773 | 168 | 30.863 | 0.945 | no | NA |
| PROV38 | CF67873 | Post-Surgery | CRC | 30 | 955166272 | 953797636 | 168 | 26.869 | 0.892 | no | NA |
| PROV38 | CF66688 | Pre-Surgery | CRC | 0 | 1163681622 | 1162396265 | 169 | 33.031 | 0.950 | yes | 0.031 |
| PROV38 | CF72805 | Post-ACT-1 | CRC | 546 | 1049389652 | 1048232747 | 169 | 29.797 | 0.947 | no | NA |
| PROV39 | CF72796 | Post-ACT-1 | CRC | 211 | 1100498296 | 1098828361 | 170 | 31.440 | 0.949 | no | NA |
| PROV39 | CF70411 | Post-Surgery | CRC | 22 | 1383530388 | 1381993445 | 171 | 40.000 | 0.950 | no | NA |
| PROV4 | CF65110 | Pre-Surgery | CRC | -15 | 1134914726 | 1133312145 | 171 | 32.898 | 0.953 | yes | 0.029 |
| PROV4 | CF67867 | Post-Surgery | CRC | 17 | 1009018682 | 1007753962 | 170 | 28.669 | 0.929 | no | NA |
| PROV4 | CF72705 | Post-ACT-1 | CRC | 287 | 1217931952 | 1216501006 | 172 | 35.586 | 0.956 | no | NA |
| PROV40 | CF67874 | Post-Surgery | CRC | 25 | 1950747542 | 1947845248 | 167 | 54.192 | 0.962 | no | NA |
| PROV40 | CF72663 | Post-ACT-1 | CRC | 202 | 1264966118 | 1263564676 | 169 | 35.738 | 0.950 | no | NA |
| PROV40 | CF66689 | Pre-Surgery | CRC | -16 | 1166051668 | 1164656326 | 168 | 32.222 | 0.951 | yes | 0.009 |
| PROV41 | CF72756 | Post-ACT-1 | CRC | 190 | 1190259134 | 1188953409 | 167 | 32.928 | 0.947 | no | NA |
| PROV41 | CF66690 | Pre-Surgery | CRC | -7 | 1281244130 | 1279711714 | 168 | 35.634 | 0.955 | yes | 0.017 |
| PROV41 | CF67875 | Post-Surgery | CRC | 12 | 1407948826 | 1405911681 | 167 | 38.707 | 0.924 | no | NA |
| PROV41 | CF82879 | Post-ACT-2 | CRC | 365 | 1277922424 | 1276324635 | 168 | 35.395 | 0.956 | yes | 0.019 |
| PROV42 | CF69893 | Post-Surgery | CRC | 3 | 1677976624 | 1676009655 | 165 | 45.535 | 0.961 | no | NA |
| PROV43 | CF65114 | Pre-Surgery | CRC | 0 | 1254688582 | 1253183089 | 166 | 33.727 | 0.932 | yes | 0.050 |
| PROV43 | CF67876 | Post-Surgery | CRC | 3 | 1075194424 | 1073229608 | 164 | 28.283 | 0.940 | yes | 0.008 |
| PROV43 | CF72643 | Post-ACT-1 | CRC | 115 | 1343118940 | 1341674102 | 165 | 36.659 | 0.947 | no | NA |
| PROV44 | CF67877 | Post-Surgery | CRC | 13 | 1505640848 | 1503650650 | 168 | 42.060 | 0.958 | no | NA |
| PROV44 | CF65115 | Pre-Surgery | CRC | 0 | 1266857140 | 1265289324 | 168 | 34.820 | 0.947 | yes | 0.046 |
| PROV45 | CF66222 | Pre-Surgery | CRC | 0 | 1358293018 | 1356821973 | 166 | 37.139 | 0.954 | yes | 0.414 |
| PROV45 | CF67878 | Post-Surgery | CRC | 3 | 1568374602 | 1566627282 | 164 | 41.618 | 0.950 | no | NA |
| PROV46 | CF70406 | Post-Surgery | CRC | 3 | 1134525386 | 1133328246 | 164 | 30.000 | 0.930 | no | NA |
| PROV46 | CF72372 | Pre-Surgery | CRC | 0 | 1565123684 | 1563290236 | 169 | 44.262 | 0.958 | yes | 0.087 |
| PROV47 | CF72392 | Pre-Surgery | CRC | 0 | 1401231922 | 1399609055 | 169 | 39.688 | 0.955 | yes | 0.149 |
| PROV47 | CF69902 | Post-Surgery | CRC | 3 | 1290962032 | 1289468146 | 165 | 34.185 | 0.949 | no | NA |
| PROV48 | CF70410 | Post-Surgery | CRC | 13 | 1216042374 | 1214660791 | 165 | 33.229 | 0.948 | yes | 0.007 |
| PROV48 | CF72792 | Post-ACT-1 | CRC | 211 | 1297560650 | 1296163540 | 173 | 38.359 | 0.957 | no | NA |
| PROV48 | CF72362 | Pre-Surgery | CRC | -15 | 968262748 | 967276275 | 172 | 28.858 | 0.915 | no | NA |
| PROV49 | CF72366 | Pre-Surgery | CRC | -15 | 1158608994 | 1157297567 | 169 | 33.295 | 0.897 | yes | 0.010 |
| PROV49 | CF69917 | Post-Surgery | CRC | 8 | 1199363712 | 1197934619 | 167 | 33.000 | 0.950 | no | NA |
| PROV49 | CF72727 | Post-ACT-1 | CRC | 149 | 1399122596 | 1397206424 | 166 | 38.871 | 0.958 | no | NA |
| PROV5 | CF69894 | Post-Surgery | CRC | 21 | 1266963616 | 1265566964 | 168 | 35.495 | 0.952 | no | NA |
| PROV5 | CF72669 | Post-ACT-1 | CRC | 225 | 1360218178 | 1358853416 | 168 | 37.690 | 0.955 | no | NA |
| PROV50 | CF72614 | Post-ACT-1 | CRC | 239 | 1568349222 | 1566661142 | 171 | 46.117 | 0.958 | no | NA |
| PROV50 | CF72367 | Pre-Surgery | CRC | -11 | 1080925376 | 1079757108 | 168 | 30.371 | 0.896 | yes | 1.171 |
| PROV50 | CF69923 | Post-Surgery | CRC | 7 | 1223488964 | 1222127276 | 166 | 34.000 | 0.940 | no | NA |
| PROV51 | CF72383 | Pre-Surgery | CRC | -8 | 1059135222 | 1058003426 | 168 | 29.774 | 0.945 | yes | 1.713 |
| PROV51 | CF72616 | Post-ACT-1 | CRC | 215 | 1373865300 | 1372417977 | 169 | 38.936 | 0.955 | no | NA |
| PROV51 | CF69980 | Post-Surgery | CRC | 22 | 1478276248 | 1476717235 | 166 | 41.119 | 0.956 | no | NA |
| PROV52 | CF72664 | Post-ACT-1 | CRC | 260 | 1369632630 | 1368045555 | 172 | 40.361 | 0.959 | yes | 0.050 |
| PROV52 | CF69901 | Post-Surgery | CRC | 17 | 1150057332 | 1148357999 | 164 | 30.627 | 0.923 | yes | 0.023 |
| PROV52 | CF72384 | Pre-Surgery | CRC | -25 | 1467012578 | 1465347568 | 172 | 43.675 | 0.956 | no | NA |
| PROV53 | CF69944 | Post-Surgery | CRC | 31 | 1186690196 | 1185328269 | 162 | 31.011 | 0.922 | no | NA |
| PROV53 | CF72655 | Post-ACT-1 | CRC | 157 | 1415526242 | 1414053276 | 167 | 40.428 | 0.955 | yes | 20.843 |
| PROV54 | CF72637 | Post-ACT-1 | CRC | 208 | 1265059696 | 1263577825 | 172 | 38.652 | 0.943 | no | NA |
| PROV54 | CF70092 | Post-Surgery | CRC | 8 | 1360230036 | 1358552339 | 165 | 36.697 | 0.955 | no | NA |
| PROV55 | CF72368 | Pre-Surgery | CRC | -8 | 1503489640 | 1501947447 | 172 | 44.159 | 0.955 | yes | 0.212 |
| PROV55 | CF69892 | Post-Surgery | CRC | 20 | 1196645246 | 1195287017 | 169 | 33.856 | 0.950 | yes | 0.013 |
| PROV56 | CF65116 | Pre-Surgery | CRC | -7 | 1069331764 | 1068017946 | 168 | 29.709 | 0.902 | yes | 0.106 |
| PROV56 | CF67880 | Post-Surgery | CRC | 26 | 1485778772 | 1483875369 | 167 | 41.329 | 0.958 | no | NA |
| PROV57 | CF69969 | Post-Surgery | CRC | 8 | 1123889760 | 1122677201 | 167 | 30.837 | 0.948 | no | NA |
| PROV57 | CF72776 | Post-ACT-1 | CRC | 237 | 1371652610 | 1370077078 | 169 | 39.990 | 0.956 | no | NA |
| PROV58 | CF72341 | Pre-Surgery | CRC | 0 | 1146974832 | 1145764148 | 171 | 33.489 | 0.949 | yes | 0.235 |
| PROV58 | CF69927 | Post-Surgery | CRC | 3 | 1151215854 | 1149826443 | 165 | 31.010 | 0.945 | no | NA |
| PROV58 | CF72679 | Post-ACT-1 | CRC | 195 | 1359410512 | 1358027797 | 172 | 40.339 | 0.956 | no | NA |
| PROV59 | CF72696 | Post-ACT-1 | CRC | 146 | 1308841020 | 1307326653 | 169 | 36.921 | 0.957 | no | NA |
| PROV59 | CF67881 | Post-Surgery | CRC | 20 | 1020978240 | 1019707303 | 172 | 29.709 | 0.933 | no | NA |
| PROV59 | CF65117 | Pre-Surgery | CRC | 0 | 1150886268 | 1149554457 | 171 | 32.925 | 0.951 | yes | 0.047 |
| PROV6 | CF70077 | Post-Surgery | CRC | 49 | 1289543000 | 1288024088 | 170 | 36.234 | 0.956 | no | NA |
| PROV60 | CF67882 | Post-Surgery | CRC | 20 | 996931306 | 995519624 | 169 | 28.112 | 0.931 | yes | 0.045 |
| PROV60 | CF65118 | Pre-Surgery | CRC | -2 | 1157849942 | 1156437607 | 169 | 32.427 | 0.950 | yes | 0.162 |
| PROV61 | CF70102 | Post-Surgery | CRC | 27 | 1188032468 | 1186686839 | 172 | 34.582 | 0.943 | no | NA |
| PROV61 | CF72605 | Post-ACT-1 | CRC | 116 | 1302670872 | 1301058528 | 171 | 38.193 | 0.959 | no | NA |
| PROV62 | CF70080 | Post-Surgery | CRC | 31 | 1190459570 | 1189026155 | 172 | 34.673 | 0.955 | no | NA |
| PROV63 | CF70075 | Post-Surgery | CRC | 35 | 1396675742 | 1394835875 | 171 | 40.092 | 0.956 | no | NA |
| PROV63 | CF72707 | Post-ACT-1 | CRC | 149 | 1435359584 | 1433662158 | 168 | 40.916 | 0.956 | no | NA |
| PROV64 | CF69970 | Post-Surgery | CRC | 15 | 1193583778 | 1192204733 | 167 | 33.091 | 0.951 | no | NA |
| PROV64 | CF72710 | Post-ACT-1 | CRC | 202 | 1435743626 | 1433995732 | 169 | 41.605 | 0.957 | no | NA |
| PROV65 | CF66138 | Pre-Surgery | CRC | 0 | 1319344780 | 1317661700 | 168 | 36.711 | 0.958 | yes | 0.017 |
| PROV65 | CF72779 | Post-ACT-1 | CRC | 168 | 1396943002 | 1395368783 | 162 | 36.155 | 0.955 | yes | 0.006 |
| PROV65 | CF67883 | Post-Surgery | CRC | 20 | 947204802 | 945921902 | 168 | 26.476 | 0.769 | no | NA |
| PROV66 | CF66139 | Pre-Surgery | CRC | -1 | 1183266368 | 1181969799 | 169 | 33.724 | 0.953 | yes | 0.016 |
| PROV66 | CF72651 | Post-ACT-1 | CRC | 140 | 1394156302 | 1392631382 | 171 | 41.120 | 0.957 | no | NA |
| PROV66 | CF67884 | Post-Surgery | CRC | 22 | 1211054738 | 1209674486 | 174 | 36.190 | 0.937 | no | NA |
| PROV67 | CF67885 | Post-Surgery | CRC | 27 | 995330656 | 993971947 | 170 | 28.715 | 0.862 | no | NA |
| PROV67 | CF66140 | Pre-Surgery | CRC | 0 | 1659086332 | 1657081962 | 169 | 47.006 | 0.957 | yes | 0.381 |
| PROV67 | CF72782 | Post-ACT-1 | CRC | 181 | 1298561816 | 1297202889 | 170 | 36.842 | 0.955 | no | NA |
| PROV68 | CF67886 | Post-Surgery | CRC | 35 | 1287355126 | 1285756211 | 169 | 36.899 | 0.952 | yes | 1.251 |
| PROV68 | CF72752 | Post-ACT-1 | CRC | 224 | 1409600578 | 1408162887 | 170 | 40.969 | 0.956 | yes | 0.306 |
| PROV68 | CF66141 | Pre-Surgery | CRC | 0 | 909335638 | 908293093 | 167 | 24.912 | 0.944 | yes | 0.924 |
| PROV68 | CF82875 | Post-ACT-2 | CRC | 434 | 1379509742 | 1377916585 | 170 | 39.702 | 0.956 | yes | 0.879 |
| PROV69 | CF72348 | Pre-Surgery | CRC | 0 | 1090955936 | 1089684097 | 169 | 31.047 | 0.936 | yes | 0.010 |
| PROV69 | CF72719 | Post-ACT-1 | CRC | 417 | 1344551860 | 1342965791 | 172 | 39.860 | 0.960 | no | NA |
| PROV69 | CF70407 | Post-Surgery | CRC | 18 | 1491003304 | 1488980623 | 168 | 41.423 | 0.959 | no | NA |
| PROV7 | CF66684 | Pre-Surgery | CRC | 0 | 1124975124 | 1123695735 | 171 | 32.160 | 0.947 | yes | 0.206 |
| PROV7 | CF67868 | Post-Surgery | CRC | 14 | 1115113126 | 1113854359 | 169 | 31.006 | 0.945 | no | NA |
| PROV70 | CF72639 | Post-ACT-1 | CRC | 275 | 1232756686 | 1231355517 | 171 | 35.626 | 0.951 | no | NA |
| PROV70 | CF70132 | Post-Surgery | CRC | 37 | 1218273118 | 1216706545 | 169 | 34.453 | 0.951 | no | NA |
| PROV70 | CF72403 | Pre-Surgery | CRC | -1 | 1256237106 | 1254932365 | 173 | 37.673 | 0.952 | yes | 0.141 |
| PROV71 | CF69956 | Post-Surgery | CRC | 3 | 1468953948 | 1467243783 | 162 | 38.368 | 0.947 | no | NA |
| PROV72 | CF72342 | Pre-Surgery | CRC | -1 | 1368609130 | 1367095579 | 170 | 39.557 | 0.954 | yes | 0.050 |
| PROV72 | CF70404 | Post-Surgery | CRC | 3 | 1279784610 | 1278236279 | 162 | 32.984 | 0.944 | yes | 0.016 |
| PROV72 | CF72681 | Post-ACT-1 | CRC | 352 | 1400941662 | 1399416819 | 167 | 39.142 | 0.958 | no | NA |
| PROV73 | CF72359 | Pre-Surgery | CRC | -1 | 1418983958 | 1417063572 | 167 | 38.731 | 0.949 | yes | 0.018 |
| PROV73 | CF70122 | Post-Surgery | CRC | 25 | 1340276104 | 1338508150 | 166 | 36.239 | 0.955 | no | NA |
| PROV73 | CF72720 | Post-ACT-1 | CRC | 216 | 1619033118 | 1617031887 | 164 | 43.022 | 0.960 | no | NA |
| PROV74 | CF70094 | Post-Surgery | CRC | 17 | 1251088840 | 1249571499 | 167 | 34.820 | 0.953 | no | NA |
| PROV74 | CF72418 | Pre-Surgery | CRC | -1 | 1397275972 | 1395648901 | 169 | 40.012 | 0.945 | yes | 0.138 |
| PROV74 | CF72634 | Post-ACT-1 | CRC | 200 | 1394667418 | 1393136843 | 166 | 38.065 | 0.953 | no | NA |
| PROV75 | CF69936 | Post-Surgery | CRC | 27 | 1089424040 | 1088116437 | 168 | 30.343 | 0.923 | no | NA |
| PROV75 | CF72631 | Post-ACT-1 | CRC | 236 | 1167960496 | 1166710611 | 172 | 33.924 | 0.946 | no | NA |
| PROV75 | CF72428 | Pre-Surgery | CRC | -2 | 1443337794 | 1441689865 | 170 | 42.080 | 0.960 | no | NA |
| PROV76 | CF69946 | Post-Surgery | CRC | 3 | 1328761546 | 1327202341 | 164 | 35.009 | 0.935 | no | NA |
| PROV76 | CF72430 | Pre-Surgery | CRC | -2 | 1558682466 | 1556650558 | 170 | 44.511 | 0.962 | yes | 0.011 |
| PROV76 | CF72625 | Post-ACT-1 | CRC | 156 | 1176057422 | 1174706577 | 166 | 32.276 | 0.952 | no | NA |
| PROV77 | CF72648 | Post-ACT-1 | CRC | 523 | 1494917266 | 1493275933 | 171 | 44.041 | 0.961 | yes | 0.015 |
| PROV77 | CF65119 | Pre-Surgery | CRC | -1 | 1080614364 | 1079230567 | 169 | 30.538 | 0.943 | yes | 0.141 |
| PROV77 | CF67887 | Post-Surgery | CRC | 3 | 1293776286 | 1292242475 | 165 | 34.483 | 0.947 | yes | 0.042 |
| PROV78 | CF67888 | Post-Surgery | CRC | 3 | 1369383600 | 1367880314 | 163 | 35.881 | 0.949 | no | NA |
| PROV78 | CF65120 | Pre-Surgery | CRC | -1 | 1367911444 | 1366155802 | 165 | 37.115 | 0.953 | yes | 0.011 |
| PROV79 | CF72704 | Post-ACT-1 | CRC | 408 | 1178354192 | 1177190221 | 174 | 34.711 | 0.954 | no | NA |
| PROV79 | CF72323 | Pre-Surgery | CRC | -1 | 1299893336 | 1298474319 | 173 | 38.474 | 0.955 | yes | 0.041 |
| PROV79 | CF70138 | Post-Surgery | CRC | 3 | 1184335012 | 1183013280 | 167 | 32.381 | 0.942 | no | NA |
| PROV8 | CF72714 | Post-ACT-1 | CRC | 395 | 1302918282 | 1301624619 | 171 | 38.454 | 0.956 | no | NA |
| PROV8 | CF69913 | Post-Surgery | CRC | 26 | 1379764406 | 1378202814 | 168 | 39.000 | 0.940 | yes | 0.010 |
| PROV8 | CF72365 | Pre-Surgery | CRC | 0 | 1066126202 | 1065029096 | 167 | 30.145 | 0.918 | yes | 0.461 |
| PROV80 | CF72738 | Post-ACT-1 | CRC | 362 | 1173762756 | 1172583916 | 170 | 33.753 | 0.945 | no | NA |
| PROV80 | CF69962 | Post-Surgery | CRC | 3 | 1290982428 | 1289541332 | 165 | 34.713 | 0.854 | no | NA |
| PROV81 | CF67889 | Post-Surgery | CRC | 12 | 1405766484 | 1404141163 | 166 | 38.196 | 0.954 | no | NA |
| PROV81 | CF72682 | Post-ACT-1 | CRC | 736 | 1177486958 | 1176324542 | 173 | 34.776 | 0.953 | no | NA |
| PROV81 | CF65122 | Pre-Surgery | CRC | -1 | 1180802520 | 1179384044 | 171 | 34.234 | 0.953 | yes | 0.273 |
| PROV82 | CF65123 | Pre-Surgery | CRC | -1 | 1188140598 | 1186634015 | 171 | 34.472 | 0.952 | yes | 0.048 |
| PROV82 | CF67890 | Post-Surgery | CRC | 3 | 1132145160 | 1130567145 | 167 | 31.529 | 0.945 | no | NA |
| PROV82 | CF72691 | Post-ACT-1 | CRC | 616 | 997295864 | 996196266 | 171 | 29.033 | 0.944 | no | NA |
| PROV83 | CF72784 | Post-ACT-1 | CRC | 485 | 1144576872 | 1143260512 | 167 | 31.776 | 0.952 | yes | 0.007 |
| PROV83 | CF70141 | Post-Surgery | CRC | 3 | 1309980478 | 1308532127 | 163 | 34.733 | 0.950 | no | NA |
| PROV83 | CF72327 | Pre-Surgery | CRC | -1 | 1303884178 | 1302396358 | 168 | 37.012 | 0.949 | yes | 0.211 |
| PROV84 | CF70142 | Post-Surgery | CRC | 3 | 1356035972 | 1354272600 | 165 | 36.294 | 0.936 | no | NA |
| PROV84 | CF72328 | Pre-Surgery | CRC | -1 | 1429766876 | 1428065793 | 170 | 41.082 | 0.957 | yes | 0.039 |
| PROV84 | CF82266 | Post-ACT-1 | CRC | 560 | 1269496200 | 1267885352 | 167 | 34.443 | 0.955 | yes | 3.244 |
| PROV84 | CF72645 | Post-ACT-2 | CRC | 366 | 1183812824 | 1182498912 | 170 | 33.785 | 0.954 | yes | 0.095 |
| PROV85 | CF72706 | Post-ACT-1 | CRC | 190 | 1345273300 | 1320876126 | 169 | 37.831 | 0.955 | no | NA |
| PROV85 | CF72330 | Pre-Surgery | CRC | -1 | 1366994956 | 1365543452 | 168 | 38.154 | 0.948 | yes | 0.366 |
| PROV85 | CF70096 | Post-Surgery | CRC | 3 | 1184936858 | 1183610504 | 164 | 31.604 | 0.937 | no | NA |
| PROV86 | CF70400 | Post-Surgery | CRC | 41 | 1465763552 | 1464181975 | 162 | 38.405 | 0.942 | no | NA |
| PROV86 | CF72723 | Post-ACT-1 | CRC | 757 | 1443236168 | 1441724457 | 169 | 41.556 | 0.956 | no | NA |
| PROV87 | CF82265 | Post-ACT-2 | CRC | 397 | 1088187222 | 1086920906 | 169 | 30.275 | 0.951 | yes | 0.009 |
| PROV87 | CF69974 | Post-Surgery | CRC | 3 | 1090176812 | 1088990496 | 168 | 30.853 | 0.950 | no | NA |
| PROV87 | CF72335 | Pre-Surgery | CRC | -1 | 1183049778 | 1181599320 | 172 | 36.728 | 0.953 | yes | 0.040 |
| PROV87 | CF82268 | Post-ACT-3 | CRC | 543 | 1550289458 | 1548582166 | 170 | 44.228 | 0.957 | yes | 0.014 |
| PROV87 | CF72746 | Post-ACT-1 | CRC | 172 | 1313987086 | 1312672250 | 172 | 39.149 | 0.953 | no | NA |
| PROV87 | CF82273 | Post-ACT-4 | CRC | 752 | 1496948482 | 1495061797 | 167 | 42.070 | 0.957 | yes | 7.425 |
| PROV88 | CF72339 | Pre-Surgery | CRC | -1 | 1304263440 | 1302660426 | 173 | 38.983 | 0.955 | yes | 0.039 |
| PROV88 | CF72650 | Post-ACT-1 | CRC | 169 | 1502699406 | 1500846111 | 170 | 43.540 | 0.961 | no | NA |
| PROV88 | CF69925 | Post-Surgery | CRC | 3 | 987207178 | 986050917 | 166 | 27.182 | 0.919 | yes | 0.027 |
| PROV89 | CF72340 | Pre-Surgery | CRC | -1 | 1416250046 | 1414500757 | 172 | 42.230 | 0.956 | yes | 0.029 |
| PROV89 | CF82272 | Post-ACT-3 | CRC | 532 | 1354774704 | 1353204909 | 169 | 38.671 | 0.956 | yes | 0.513 |
| PROV89 | CF69930 | Post-Surgery | CRC | 3 | 1472991446 | 1471357568 | 164 | 39.207 | 0.948 | no | NA |
| PROV89 | CF72780 | Post-ACT-1 | CRC | 187 | 1394616256 | 1393157494 | 168 | 39.745 | 0.956 | no | NA |
| PROV89 | CF82267 | Post-ACT-2 | CRC | 369 | 1158322642 | 1157086144 | 167 | 31.755 | 0.952 | yes | 0.007 |
| PROV9 | CF69904 | Post-Surgery | CRC | 41 | 1228505016 | 1226963550 | 172 | 35.507 | 0.922 | no | NA |
| PROV9 | CF72395 | Pre-Surgery | CRC | 0 | 1301877994 | 1300233631 | 176 | 39.781 | 0.959 | yes | 0.021 |
| PROV90 | CF72344 | Pre-Surgery | CRC | -1 | 1355197538 | 1353679549 | 170 | 39.371 | 0.951 | yes | 0.091 |
| PROV90 | CF72791 | Post-ACT-1 | CRC | 348 | 1346257922 | 1344911777 | 170 | 38.593 | 0.956 | no | NA |
| PROV90 | CF70082 | Post-Surgery | CRC | 3 | 1230337630 | 1229058655 | 163 | 32.624 | 0.951 | no | NA |
| PROV91 | CF72750 | Post-ACT-1 | CRC | 217 | 1356046146 | 1354385996 | 173 | 39.842 | 0.958 | no | NA |
| PROV91 | CF70078 | Post-Surgery | CRC | 3 | 953668240 | 952541182 | 166 | 26.089 | 0.938 | no | NA |
| PROV92 | CF69929 | Post-Surgery | CRC | 3 | 883857106 | 882845612 | 166 | 24.129 | 0.915 | no | NA |
| PROV92 | CF72690 | Post-ACT-1 | CRC | 223 | 1341037312 | 1339544304 | 173 | 39.302 | 0.958 | no | NA |
| PROV93 | CF82276 | Post-ACT-4 | CRC | 723 | 1451881820 | 1449983099 | 169 | 41.608 | 0.961 | no | NA |
| PROV93 | CF72346 | Pre-Surgery | CRC | -1 | 1233464082 | 1231903274 | 170 | 35.491 | 0.927 | no | NA |
| PROV93 | CF82269 | Post-ACT-2 | CRC | 348 | 1383579080 | 1381599318 | 169 | 39.183 | 0.960 | no | NA |
| PROV93 | CF70083 | Post-Surgery | CRC | 3 | 1278276128 | 1276730750 | 164 | 33.731 | 0.954 | no | NA |
| PROV93 | CF82274 | Post-ACT-3 | CRC | 562 | 1389777708 | 1387849103 | 168 | 39.510 | 0.960 | no | NA |
| PROV93 | CF72786 | Post-ACT-1 | CRC | 181 | 1396713592 | 1395123951 | 166 | 38.768 | 0.958 | no | NA |
| PROV94 | CF72721 | Post-ACT-1 | CRC | 399 | 1334288162 | 1332896872 | 169 | 38.320 | 0.955 | no | NA |
| PROV94 | CF70114 | Post-Surgery | CRC | 3 | 1023192132 | 1022071102 | 165 | 27.469 | 0.839 | no | NA |
| PROV95 | CF67891 | Post-Surgery | CRC | 10 | 1174882684 | 1173276787 | 169 | 32.755 | 0.850 | no | NA |
| PROV95 | CF66692 | Pre-Surgery | CRC | -3 | 1334395090 | 1332765047 | 167 | 36.710 | 0.953 | yes | 0.005 |
| PROV95 | CF72662 | Post-ACT-1 | CRC | 181 | 1314079578 | 1312565501 | 169 | 37.428 | 0.956 | no | NA |
| PROV96 | CF67894 | Post-Surgery | CRC | 11 | 1250817116 | 1248873236 | 170 | 35.594 | 0.951 | no | NA |
| PROV96 | CF72798 | Post-ACT-1 | CRC | 174 | 1346839482 | 1345368235 | 172 | 39.999 | 0.955 | no | NA |
| PROV97 | CF67895 | Post-Surgery | CRC | 3 | 1268641162 | 1267017074 | 164 | 33.662 | 0.946 | no | NA |
| PROV97 | CF65124 | Pre-Surgery | CRC | -1 | 1043569850 | 1042165769 | 169 | 29.741 | 0.945 | yes | 0.025 |
| PROV98 | CF65125 | Pre-Surgery | CRC | 0 | 1308439684 | 1306931268 | 169 | 37.288 | 0.955 | yes | 1.511 |
| PROV98 | CF67896 | Post-Surgery | CRC | 3 | 1355109130 | 1353581648 | 165 | 36.358 | 0.942 | no | NA |
| PROV98 | CF72672 | Post-ACT-1 | CRC | 165 | 1392716206 | 1391132083 | 167 | 38.034 | 0.957 | no | NA |
| PROV99 | CF82264 | Post-ACT-3 | CRC | 541 | 1011832892 | 1010526921 | 169 | 28.127 | 0.947 | yes | 0.108 |
| PROV99 | CF67897 | Post-Surgery | CRC | 3 | 1179374882 | 1177932483 | 166 | 32.302 | 0.948 | yes | 1.029 |
| PROV99 | CF82261 | Post-ACT-2 | CRC | 357 | 1092075652 | 1090653880 | 171 | 30.848 | 0.952 | yes | 0.040 |
| PROV99 | CF65126 | Pre-Surgery | CRC | -25 | 1237897924 | 1236315027 | 171 | 36.009 | 0.956 | yes | 3.360 |
| PROV99 | CF72699 | Post-ACT-1 | CRC | 273 | 1336409112 | 1334922244 | 171 | 39.001 | 0.959 | no | NA |

**Supplementary Table 6.** Univariable cox regression analyses for Post-surgery ctDNA status, pathological risk factors and MSI status.

| **Risk variable** | **Groups** | **% Patients** | **HR** | **95% CI** | **p.value** |
| --- | --- | --- | --- | --- | --- |
| **Post-surgery ctDNA status** | ctDNA-negative | 181 |  |  |  |
|  | ctDNA-positive | 28 | 6.17 | 3.4-11.2 | 1.64E-09 |
| **Pathological risk** | Low risk | 127 |  |  |  |
|  | High risk | 82 | 3.54 | 1.9-6.5 | 4.12E-05 |
| **T status** | T1-3 | 158 |  |  |  |
|  | T4 | 51 | 2.99 | 1.7-5.3 | 1.93E-04 |
| **N status** | N1 | 154 |  |  |  |
|  | N2 | 55 | 3.17 | 1.8-5.6 | 8.04E-05 |
| **MSI status** | Stable (MSS) | 179 |  |  |  |
|  | Unstable (MSI) | 30 | 0.37 | 0.1-1.2 | 0.094 |

ctDNA, circulating tumour DNA; T, Tumour; N, Node; MSI, microsatellite instability; MSS, Microsatellite stability

**Supplementary Table 7**. Univariable cox regression analyses per individual pathological risk variable and MSI status, stratified for ctDNA status.

| **Risk variable** | **Groups** | **ctDNA status** | **HR** | **95% CI** | **p.value** |
| --- | --- | --- | --- | --- | --- |
| **Pathological risk + Post-surgery ctDNA status** | Low risk | ctDNA-negative | Reference |  |  |
|  | Low risk | ctDNA-positive | 11.74 | 4.4-31.6 | 1.06E-06 |
|  | High risk | ctDNA-negative | 5.65 | 2.4-13.2 | 6.70E-05 |
|  | High risk | ctDNA-positive | 28.45 | 10.5-77.2 | 4.84E-11 |
| **T status + Post-surgery ctDNA status** | T1-3 | ctDNA-negative | Reference |  |  |
|  | T1-3 | ctDNA-positive | 6.43 | 3.0-14.0 | 2.85E-06 |
|  | T4 | ctDNA-negative | 3.28 | 1.6-6.8 | 0.001 |
|  | T4 | ctDNA-positive | 39.10 | 15.1-101.2 | 4.16E-14 |
| **N status + Post-surgery ctDNA status** | N1 | ctDNA-negative | Reference |  |  |
|  | N1 | ctDNA-positive | 8.70 | 3.9-19.4 | 1.36E-07 |
|  | N2 | ctDNA-negative | 4.14 | 2.0-8.6 | 1.43E-04 |
|  | N2 | ctDNA-positive | 16.79 | 6.66-42.5 | 2.69E-09 |
| **MSI status + Post-surgery ctDNA status** | Unstable (MSI) | ctDNA-negative | Reference |  |  |
|  | Unstable (MSI) | ctDNA-positive | 7.11 | 0.4-78.4 | 0.109 |
|  | Stable (MSS) | ctDNA-negative | 2.59 | 0.6-10.9 | 0.192 |
|  | Stable (MSS) | ctDNA-positive | 15.42 | 3.5-66.9 | 2.60E-04 |

"ctDNA status" corresponds to the post-surgery time point. T, Tumour; N, Node; MSI, microsatellite instability; MSS, Microsatellite stablility

**Supplementary Table 8**. Likelihood Ratio Test for model goodness-of-fit assessment.

Multivariable Cox regression models were fitted including different pathological variables and MSI status. Four likelihood ratio tests were performed to assess the added value of ctDNA status in each model.

|  | **Predictor combinations in cox regression model** | **P-value** | **Better model** |
| --- | --- | --- | --- |
| **LRT 1** | Pathological_Risk | 1.71E-08 | Pathological_Risk + **ctDNA status** |
|  | Pathological_Risk + **ctDNA status** |  |  |
| **LRT 2** | Pathological_Risk + MSI status | 3.91E-08 | Pathological_Risk + MSI + **ctDNA status** |
|  | Pathological_Risk + MSI + **ctDNA status** |  |  |
| **LRT 3** | T status + N status | 6.34E-09 | T status + N status + **ctDNA status** |
|  | T status + N status + **ctDNA status** |  |  |
| **LRT 4** | T status + N status + MSI status | 1.06E-08 | T status + N status + MSI status + **ctDNA status** |
|  | T status + N status + MSI status + **ctDNA status** |  |  |

"ctDNA status" corresponds to the post-surgery timepoint. LRT, Likelihood Ratio Test; T, Tumour; N, Node; MSI, microsatellite instability

**Supplementary Table 9.** Multivariable cox regression model analysis with hazard ratio per variable inluded.

| **Model** | **Covariate** | **Reference** | **Hazard Ratio** | **95% CI** | **p-value** |
| --- | --- | --- | --- | --- | --- |
| **Pathological_Risk + MSI status +  ctDNA status** | Pathologcal risk: High-risk | Low-risk | 3.79 | 2.1-7.0 | 1.78E-05 |
|  | MSI status: Stable | Instable | 0.73 | 0.6-6.7 | 0.225 |
|  | ctDNA status: ctDNA-positive | ctDNA-negative | 6.55 | 3.6-11.9 | 7.61E-10 |
| **T status + N status +  MSI status + ctDNA status** | T status: T4 | T1-3 | 3.22 | 1.7-6.1 | 2.60E-04 |
|  | N status: N2 | N1 | 2.73 | 1.2-4.0 | 0.013 |
|  | MSI status: Stable | Instable | 1.99 | 0.6-6.6 | 0.253 |
|  | ctDNA status: ctDNA-positive | ctDNA-negative | 7.53 | 4.3-14.5 | 2.35E-10 |

ctDNA status corresponds to the post-surgery time point. T, Tumour; N, Node; MSI, microsatellite instability; MSS, Microsatellite stability

**References**

1. Arora, K., et al., *Deep whole-genome sequencing of 3 cancer cell lines on 2 sequencing platforms.* Sci Rep, 2019. **9**(1): p. 19123.

2. Watkins, T.N., et al., *Abstract 5597: Enhanced detection and classification of cell-free DNA alterations through matched normal analyses with PGDx elioTM plasma complete.* Cancer Research, 2023. **83**(7_Supplement): p. 5597-5597.

3. Wood, D.E., et al., *A machine learning approach for somatic mutation discovery.* Sci Transl Med, 2018. **10**(457).

4. Keefer, L.A., et al., *Automated next-generation profiling of genomic alterations in human cancers.* Nat Commun, 2022. **13**(1): p. 2830.

5. Sjoblom, T., et al., *The consensus coding sequences of human breast and colorectal cancers.* Science, 2006. **314**(5797): p. 268-74.

6. Wang, T.L., et al., *Prevalence of somatic alterations in the colorectal cancer cell genome.* Proc Natl Acad Sci U S A, 2002. **99**(5): p. 3076-80
